# Combining Atomic Layer Deposition with Surface Organometallic Chemistry to Enhance Atomic-Scale Interactions and Improve the Activity and Selectivity of Cu–Zn/SiO_2_ Catalysts for the Hydrogenation of CO_2_ to Methanol

**DOI:** 10.1021/jacsau.3c00319

**Published:** 2023-08-23

**Authors:** Hui Zhou, Scott R. Docherty, Nat Phongprueksathat, Zixuan Chen, Andrey V. Bukhtiyarov, Igor P. Prosvirin, Olga V. Safonova, Atsushi Urakawa, Christophe Copéret, Christoph R. Müller, Alexey Fedorov

**Affiliations:** † Department of Mechanical and Process Engineering, ETH Zürich, CH-8092 Zürich, Switzerland; ‡ Department of Energy and Power Engineering, Tsinghua University, 100084 Beijing, China; § Department of Chemistry and Applied Biosciences, ETH Zürich, CH-8093 Zürich, Switzerland; ∥ Department of Chemical Engineering, Delft University of Technology, 2629 HZ Delft, The Netherlands; ⊥ Synchrotron Radiation Facility SKIF, Boreskov Institute of Catalysis SB RAS, 630559 Kol’tsovo, Russia; # Boreskov Institute of Catalysis, SB RAS, 630090 Novosibirsk, Russia; ¶ Paul Scherrer Institute, CH-5232 Villingen, Switzerland

**Keywords:** CO_2_ hydrogenation, CuZn alloy, dealloying, SOMC, ALD, operando DRIFTS

## Abstract

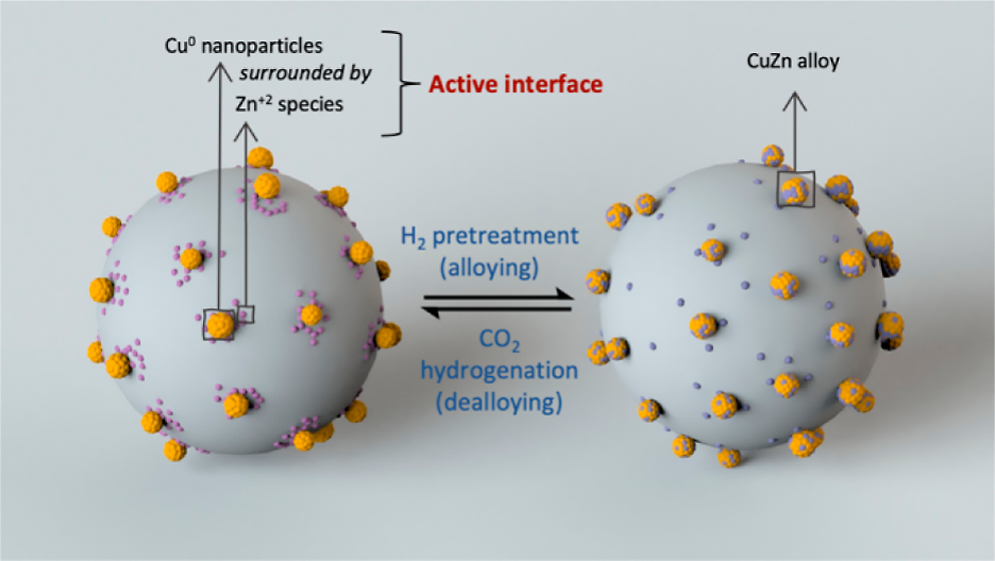

The direct synthesis of methanol via the hydrogenation of CO_2_, if performed efficiently and selectively, is potentially a powerful technology for CO_2_ mitigation. Here, we develop an active and selective Cu–Zn/SiO_2_ catalyst for the hydrogenation of CO_2_ by introducing copper and zinc onto dehydroxylated silica via surface organometallic chemistry and atomic layer deposition, respectively. At 230 °C and 25 bar, the optimized catalyst shows an intrinsic methanol formation rate of 4.3 g h^–1^ g_Cu_^–1^ and selectivity to methanol of 83%, with a space-time yield of 0.073 g h^–1^ g_cat_^–1^ at a contact time of 0.06 s g mL^–1^. X-ray absorption spectroscopy at the Cu and Zn K-edges and X-ray photoelectron spectroscopy studies reveal that the CuZn alloy displays reactive metal support interactions; that is, it is stable under H_2_ atmosphere and unstable under conditions of CO_2_ hydrogenation, indicating that the dealloyed structure contains the sites promoting methanol synthesis. While solid-state nuclear magnetic resonance studies identify methoxy species as the main stable surface adsorbate, transient operando diffuse reflectance infrared Fourier transform spectroscopy indicates that μ-HCOO*(ZnO_*x*_) species that form on the Cu–Zn/SiO_2_ catalyst are hydrogenated to methanol faster than the μ-HCOO*(Cu) species that are found in the Zn-free Cu/SiO_2_ catalyst, supporting the role of Zn in providing a higher activity in the Cu–Zn system.

## Introduction

The conversion of CO_2_ into methanol or other bulk chemicals is a promising approach to mitigate anthropogenic CO_2_ emissions.^1^ Among various CO_2_-derived products, methanol, a liquid platform chemical and fuel, is often considered as a chemical of choice to store H_2_ obtained through renewable (green) electricity.^2^ In general, Cu-based catalysts show superior activity in the hydrogenation of CO_2_ to methanol as compared to catalysts based on other nonprecious transition metals.^3–7^ In this context, the Cu–ZnO–Al_2_O_3_ catalyst is one of the most widely studied catalysts for the CO_2_ hydrogenation. The interplay of Cu and Zn species in this system is believed to be important for its catalytic function.^8–12^ Yet the Cu–ZnO–Al_2_O_3_ catalyst still suffers, especially at relatively low reaction pressures (20–50 bar) from its modest activity and low selectivity to methanol, owing to the competing formation of CO and steam, presumably via the reverse water–gas shift (RWGS) reaction; further, the high concentration of H_2_O affects negatively the long-term stability of this catalyst.^2,13,14^ In addition to the RWGS reaction, it was recently shown that also methanol decomposition to CO influences notably the loss of methanol selectivity, which is typically observed for an increasing conversion of CO_2_.^15^ Furthermore, the complexity and heterogeneity of the industrial Cu–ZnO–Al_2_O_3_ catalyst make in-depth mechanistic studies challenging.^10,16,17^ For instance, the role of zinc in this catalyst formulation, specifically the alloying and dealloying of Zn with Cu and the nature of interfacial Cu–ZnO sites, is not fully understood and is highly debated.^2,8,10,17–21^

In this context, the use of model catalysts, such as Cu–Zn/SiO_2_, allows to partially alleviate the complexity of the Cu–ZnO–Al_2_O_3_ formulation and may facilitate the performance of structure–activity studies.^7,19,22–24^ In particular, the elucidation of the promoting role of Zn in the hydrogenation of CO_2_ can be a challenge since only a fraction of all Zn species may contribute to the promoting effect.^25^ Therefore, the development of approaches to minimize spectator Zn species in model Cu–Zn catalysts through, e.g., the optimization of the Zn loading and maximizing the atomic-scale interaction of Zn and Cu is vital to reveal the state of promoting Zn species in active Cu-based catalysts. For instance, it has been reported that in a stream of CO_2_-rich syngas, the speciation of an active Zn promoter in a Cu–Zn/SiO_2_ catalyst features mostly oxidized ZnO_*x*_ species with only a small fraction of metallic Zn,^19^ consistent with other reports.^23,24^ In contrast, a recent study of a Cu–Zn/SiO_2_ catalyst reported that Zn is mostly metallic (average oxidation state of +0.8) even at 20 bar of H_2_/CO_2_ (3:1) at 260 °C.^22^ Hence, additional studies to clarify the role and speciation of the active Zn promoter in a working Cu–Zn-based CO_2_ hydrogenation catalyst remain pertinent.

Surface organometallic chemistry (SOMC) is an approach to yield catalysts featuring uniform, size-controlled, and highly dispersed nanoparticles (NPs).^26^ SOMC is based on the grafting of molecular precursors onto a support with a controlled density of reactive surface sites (typically hydroxyl groups, with their surface density controlled by the dehydroxylation temperature).^27–29^ The grafted species are highly dispersed on the support, and a subsequent H_2_ pretreatment yields uniformly sized and well-distributed supported NPs, such as 2–4 nm Cu NPs on silica.^5,28,29^ However, Cu/SiO_2_ catalysts prepared via SOMC display only low activity and high CO selectivity when tested under CO_2_ hydrogenation conditions, but both their activity and methanol selectivity can be improved in the presence of promoter species (such as those formed in the presence of Zr^4+^, Ga^3+^, or Zn^2+^ sites bound to silica).^5,7,30^

Further, a controlled engineering of the metal/support interface will benefit the elucidation of robust structure–activity relationships.^31^ In this context, atomic layer deposition (ALD) can be a method of choice and has been used in the preparation of heterogeneous catalysis, e.g., by forming an overcoat of controlled thickness (e.g., a metal oxide) onto a support or a catalyst.^32,33^ In particular, ALD enables a high conformality of the grown films and their tunable composition and thickness down to the sub-nm scale.^34,35^ In addition, ALD allows to manipulate in a controlled fashion the relative abundance and distribution of Brønsted and Lewis (strong and weak) acid sites.^36–38^ Yet, the use of ALD to create heterogeneous catalysts with well-defined metal/support interfaces remains relatively underexplored.^39–41^

Here, we report on the use of a combined SOMC-ALD approach to prepare active and selective Cu–Zn/SiO_2_ catalysts for the hydrogenation of CO_2_ to methanol. More specifically, a highly active and selective catalyst was prepared by exposing dehydroxylated silica that contains grafted mesityl (Mes, 2,4,6-trimethylphenyl) copper species to ALD pulses of diethylzinc (Et_2_Zn), followed by H_2_ pretreatment (500 °C, 2 h). This synthetic approach gives a catalyst with an intrinsic methanol formation rate of 4.3 g h^–1^ g_Cu_^–1^ at 83% selectivity to methanol at 230 °C and 25 bar, which is higher than the methanol formation rates displayed by other related Cu-based catalysts evaluated under similar conditions (vide infra). X-ray absorption spectroscopy (XAS) and X-ray photoelectron spectroscopy (XPS) reveal that the high activity and selectivity to methanol of the prepared catalyst are due to a facile dealloying of the as-prepared CuZn phase under reactive conditions, yielding an active Cu–Zn^2+^ interface. Transient operando diffuse reflectance infrared Fourier transform spectroscopy (DRIFTS) demonstrates that μ-HCOO*(Zn^2+^) are key intermediate species on the active and selective Cu–Zn/SiO_2_ catalyst and are converted faster into methanol than μ-HCOO*(Cu) species that are found on the reference Cu/SiO_2_ catalyst, providing an explanation for the superior catalytic activity of the Cu–Zn over the Cu system.

## Results and Discussion

### Synthesis and Characterization of Cu–Zn/SiO_2_

**Figure 1:**
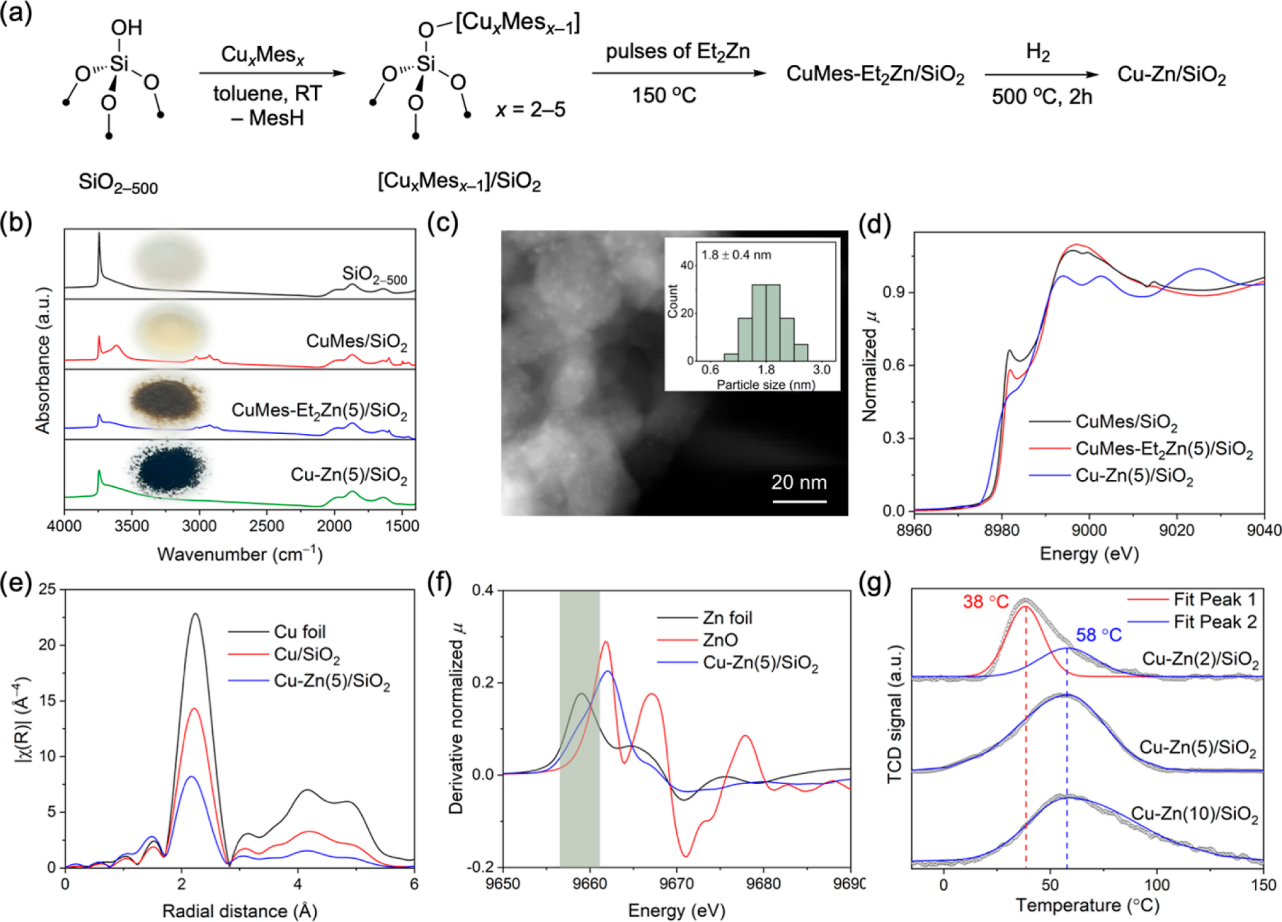
(a) Schematic of the synthesis of Cu–Zn/SiO_2_. (b) Infrared spectra of the prepared materials. (c) HAADF-STEM of Cu–Zn(5)/SiO_2_. Inset: Cu particle size distribution. (d) Cu K-edge XANES spectra of CuMes/SiO_2_, CuMes-Et_2_Zn(5)/SiO_2_, and Cu–Zn(5)/SiO_2_ (there is a glitch for CuMes/SiO_2_ at about 9014 eV). (e) Fourier transform of k^3^-weighted Cu K-edge EXAFS. (f) First derivative of the XANES spectra at the Zn K-edge. (g) H_2_ TPD after saturation in 5% H_2_/Ar. Except for panel (c), the characterization data for Cu–Zn/SiO_2_ and Cu/SiO_2_ were obtained from pristine materials after pretreatment under a flow of undiluted H_2_ at 500 °C for 2 h.

[Cu_*x*_Mes_*x*_] clusters, where *x* = 2, 4, 5,^42^ were grafted onto SiO_2–500_ (Aerosil-300, dehydroxylated at 500 °C, 296 m^2^ g^–1^ with ca. 1.2 OH nm^–2^ according to titration with benzyl magnesium bromide) to yield CuMes/SiO_2_, as described previously (Figure 1a).^26,29,30,43^ The transmission infrared spectrum of CuMes/SiO_2_ contains a band due to ≡SiOH at 3744 cm^–1^, a broad band at 3616 cm^–1^ (silanols interacting with CuMes and vicinal silanols), and bands at 3024, 2925, 2870, and 1598 cm^–1^ due to the grafted CuMes (Figure 1b).^43^ Exposure of CuMes/SiO_2_ in an ALD chamber to five pulses of Et_2_Zn at 150 °C (pulse duration of 0.1 s) yielded the material CuMes-Et_2_Zn(5)/SiO_2_. CuMes-Et_2_Zn(5)/SiO_2_ features decreased intensities of the bands at 3744 and 3616 cm^–1^ (Figure 1b). This can be explained by the reaction of Et_2_Zn with residual ≡SiOH sites. However, while CuMes/SiO_2_ is a pale yellow material, CuMes-Et_2_Zn(5)/SiO_2_ is dark brown and a material prepared by the same Zn deposition method, but using SiO_2–500_ instead of CuMes/SiO_2_ is colorless [denoted Et_2_Zn(5)/SiO_2_, Figure S17]. This observation is consistent with a reduction of the grafted CuMes sites by Et_2_Zn at 150 °C. Indeed, Cu NPs of ca. 1.8 ± 0.4 nm in diameter are observed in CuMes-Et_2_Zn(5)/SiO_2_ by high-angle annular dark-field scanning transmission electron microscopy (HAADF-STEM) (passivated in 1% O_2_/N_2_ and then exposed to air before the transfer to the microscope, vide infra, Figure S18). STEM–EDX analysis further shows that while Zn is distributed rather homogeneously, the Zn contrast is slightly higher at and around the Cu NPs (Figure S19). That being said, no NPs are observed by HAADF-STEM in CuMes/SiO_2_ before the deposition of Et_2_Zn (Figures S20 and S21), indicating that the interaction between the grafted Cu precursor and Et_2_Zn at 150 °C is required to form such NPs. Treatment under undiluted H_2_ at 500 °C for 2 h leads to the complete disappearance of the C–H stretching bands; however, the isolated silanols are only partially restored (Figure 1b). This is different from what was observed for Cu/SiO_2_ prepared by SOMC^29,43^ and indicates an interaction between silanols and Zn species (likely amorphous zinc oxide ZnO_*x*_).

The specific surface areas of Cu–Zn(5)/SiO_2_, Cu/SiO_2_, and Zn(5)/SiO_2_ are 247, 268, and 291 m^2^ g^–1^, respectively, i.e., slightly lower than that of the SiO_2_ support (296 m^2^ g^–1^, Table S1). The Cu loadings, determined by inductively coupled plasma optical emission spectroscopy (ICP-OES), in Cu–Zn(5)/SiO_2_ and Cu/SiO_2_ are similar, i.e., 2.1 and 2.0 wt %, respectively. The loadings of Zn in CuMes-Et_2_Zn(5)/SiO_2_ and Cu–Zn(5)/SiO_2_ are 0.5 and 0.4 wt %, respectively (Table S2), indicating a minor loss of Zn during H_2_ treatment owing to the formation of volatile Zn species during high-temperature H_2_ treatment. A more notable loss of Zn, from 0.8 to 0.2 wt %, is observed during the H_2_ treatment at 500 °C for 2 h of Et_2_Zn(5)/SiO_2_. On the contrary, no Zn loss occurs during the H_2_ treatment of CuMes-Et_2_Zn(5)/SiO_2_ at 300 °C for 2 h (Table S2).

In addition to Cu–Zn(5)/SiO_2_, two reference catalysts were prepared utilizing two or ten pulses of Et_2_Zn onto CuMes/SiO_2_. These Cu–Zn(2)/SiO_2_ and Cu–Zn(10)/SiO_2_ catalysts contain Zn with loadings of 0.1 and 0.5 wt %, respectively. The Cu loadings in both materials are 2.1 wt % (Figure S22).

HAADF-STEM imaging of Cu–Zn(5)/SiO_2_ shows small Cu NPs with a narrow size distribution of 1.8 ± 0.4 nm in diameter (Figures 1c and S23), that is, of a similar size as observed in as-prepared CuMes-Et_2_Zn(5)/SiO_2_ and in Cu–Zn(5)/SiO_2_ after treatment in H_2_ at 300 °C for 2 h (Figures S2 and S18). The Cu particle size in Cu–Zn(5)/SiO_2_ is smaller than that found for Cu/SiO_2_, i.e., 2.9 ± 0.4 nm (Figure S24). This is possibly due to the interaction between Cu and Zn since Zn is found to be enriched around Cu NPs in the EDX mappings (Figure S25). The powder X-ray diffraction pattern of Cu–Zn(5)/SiO_2_ contains a weak peak at 36.5° (Figure S26), likely due to Cu_2_O, consistent with the XPS and X-ray absorption near edge structure (XANES) results discussed below. No NPs are observed in Zn(5)/SiO_2_ (Figures S27 and S28).

The Cu K-edge XANES spectrum of pristine CuMes-Et_2_Zn(5)/SiO_2_ (that is, recorded ex situ without exposure to air) shows clearly a decrease of the Cu^1+^ feature at 8982 eV compared to grafted CuMes/SiO_2_ (Figure 1d). This result is consistent with the reduction of the grafted CuMes sites during the pulsing of Et_2_Zn at 150 °C, the color change, and the observation of NPs in TEM. The complete disappearance of the feature at 8982 eV confirms a further reduction of Cu in Cu–Zn(5)/SiO_2_. Compared with the Cu foil reference, a pre-edge feature with a maximum at ca. 8980 eV that is less developed and slightly shifted to higher energies is observed for Cu/SiO_2_; this difference is explained by the morphological difference of Cu in Cu NPs of Cu/SiO_2_ and bulk Cu in a Cu foil (Figure S29).^6,7^ A further shift in this region is observed for Cu–Zn(5)/SiO_2_, consistent with the presence of even smaller Cu NPs in Cu–Zn(5)/SiO_2_ relative to Cu/SiO_2_. The fitting of the Cu K-edge EXAFS data is also in agreement with the presence of smaller NPs in Cu–Zn(5)/SiO_2_, evidenced by the lower peak intensity of the Cu–Cu shell and a smaller coordination number (CN) of Cu–Zn(5)/SiO_2_ compared to Cu/SiO_2_, i.e., 5.8(9) vs 8.7(6) (Figures 1e, S3 and Table S3).

The Zn K-edge XANES data suggest the presence of similar Zn states in CuMes-Et_2_Zn(5)/SiO_2_ and Cu–Zn(5)/SiO_2_, with the edge position found at ca. 9662 eV for both materials (Figure S31). This means that Zn^2+^ sites remain in Cu–Zn(5)/SiO_2_ even after H_2_ treatment at 500 °C for 2 h (Figure S32). Noteworthy, the different white line profiles of Cu–Zn(5)/SiO_2_ and the ZnO reference further suggest that Zn exists mainly as Zn^2+^ sites in Cu–Zn(5)/SiO_2_, probably in the form of a dispersed amorphous zinc oxide ZnO_*x*_ phase on the silica surface. Consistent with this conclusion is the effective absence of a second coordination sphere due to Zn–Zn paths in the EXAFS data of Cu–Zn(5)/SiO_2_ (Figure S32). Importantly, the presence of metallic Zn in Cu–Zn(5)/SiO_2_ is also identified in the derivative XANES spectrum due to a shoulder at 9659 eV (Figure 1f). The presence of this shoulder is indicative of the formation of either Zn^0^ or a CuZn alloy in Cu–Zn(5)/SiO_2_.^7^ Control experiments show that the feature at 9659 eV is not observed in the commercial Cu–ZnO–Al_2_O_3_ catalyst H_2_-pretreated at 250 °C (Figure S33).

The amounts of surface Cu^0^ sites determined by N_2_O titration in Cu/SiO_2_, Cu–Zn(2)/SiO_2_, Cu–Zn(5)/SiO_2_, and Cu–Zn(10)/SiO_2_ are 124, 133, 126, and 101 μmol (Cu^0^) g_cat_^–1^, and these amounts account for ca. 39, 40, 40, and 29% of the total Cu loading in these materials, respectively (Table S4). Note that given the notably lower Zn weight loading in the prepared materials, viz., ca. 5 times lower Zn amount in Cu–Zn(5)/SiO_2_ than Cu, and that according to Zn K-edge XANES, there is more Zn^2+^ than Zn^0^ in pristine (but measured ex situ) Cu–Zn(5)/SiO_2_ (Figure 1f), the amounts of surface Cu^0^ reported above were obtained by assuming no competing oxidation of Zn^0^ (or anionic oxygen vacancies in ZnO_*x*_) by N_2_O.^17^ The higher amount of Cu^0^ surface sites in Cu–Zn(2)/SiO_2_ relative to Cu/SiO_2_ correlates with the smaller NP size in Cu–Zn(2)/SiO_2_. The lower number of surface Cu^0^ sites in Cu–Zn(10)/SiO_2_ may be due to a partial blocking of Cu^0^ sites by ZnO_*x*_ with an increasing number of ALD pulses.

To investigate the redox properties of Cu in the prepared materials, we compared their H_2_ TPR behavior after treatment of the reduced materials in 5% O_2_ at room temperature (Figure S34). While Zn(5)/SiO_2_ does not consume a detectable amount of H_2_, the total H_2_ consumption of other studied materials is consistent with the theoretical value calculated from the Cu loading determined by ICP (Table S5). According to the consumed amount of H_2_ and the Cu loading, most of the Cu sites in Cu–Zn(5)/SiO_2_ were oxidized to CuO and then reduced to metallic Cu, whereby the temperature at the maximum H_2_ consumption rate is 161 °C. The temperature of the maximum H_2_ consumption rate of the commercial Cu–ZnO–Al_2_O_3_ catalyst is found at 158 °C, i.e., close to that of Cu–Zn(5)/SiO_2_, while the temperature at the maximum H_2_ consumption rate of Cu/SiO_2_ is ca. 7 °C higher than that of Cu–Zn(5)/SiO_2_. These results indicate that the presence of Zn eases to a minor extent the reduction of CuO, possibly due to the formation of a CuZn alloy.

H_2_ temperature-programed desorption (TPD) experiments allow for a comparison of the nature and quantity of (surface) Cu sites in the reduced Cu–Zn/SiO_2_ materials (prepared in situ from the respective passivated materials; vide infra). We have reported previously that Cu/SiO_2_ has a H_2_ desorption peak centered at 20 °C.^30^ In general, Cu-based catalysts feature a H_2_ desorption peak in the temperature range of 30–60 °C (due to chemisorbed H_2_).^44,45^ In Cu–ZnO–Al_2_O_3_ catalysts with significant amounts of a CuZn alloy, the desorption peak occurs at a higher temperature.^46^ Cu–Zn(2)/SiO_2_ shows a peak centered at 38 °C and a shoulder at 58 °C, while Cu–Zn(5)/SiO_2_ features an asymmetric peak with a maximum at 58 °C (Figure 1g). Generally, the H_2_-TPD profile of Cu–Zn(10)/SiO_2_ is similar to that of Cu–Zn(5)/SiO_2_, yet a notable tailing toward higher desorption temperatures is observed for this material. The appearance of a higher temperature desorption peak at 58 °C for Cu–Zn/SiO_2_ materials indicates a modification of the Cu surface sites by Zn (alloying).^46^ Similar results were observed by H_2_ TPD of Cu–ZrO_2_ and Cu–ZnO–ZrO_2_ catalysts.^47^

### CO_2_ Hydrogenation Tests

**Figure 2:**
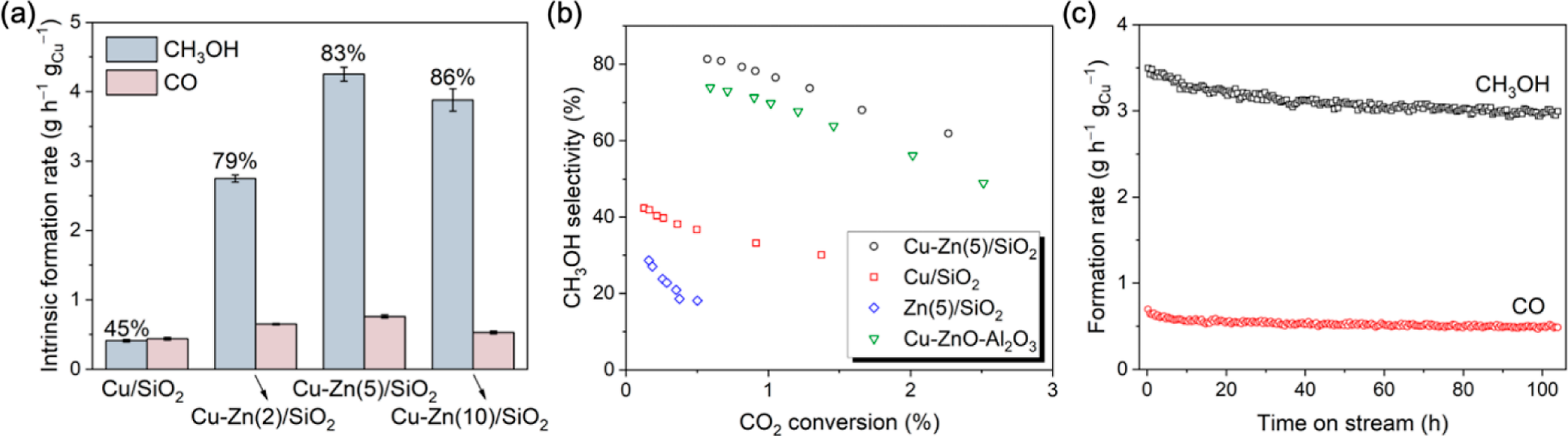
(a) Intrinsic formation rates of CH_3_OH and CO (230 °C, 25 bar, H_2_/CO_2_/N_2_ = 3:1:1) obtained by extrapolation to zero conversion (zero contact time, see Figure S36) together with the selectivity for CH_3_OH specified above the respective bars. (b) CH_3_OH selectivity as a function of CO_2_ conversion. (c) Stability test of Cu–Zn(5)/SiO_2_ over ca. 100 h of TOS (230 °C, 25 bar, H_2_/CO_2_/N_2_ = 3:1:1, contact time 0.06 s g mL^–1^). The catalytic performance of Cu/SiO_2_ has been reported by us previously and is reproduced here for the sake of comparison.^30^

The catalytic performance of the series of Cu–Zn/SiO_2_ catalysts for CO_2_ hydrogenation was evaluated at 230 °C and 25 bar (H_2_/CO_2_/N_2_ = 3:1:1). Activated catalysts were prepared in situ starting from the respective CuMes-Et_2_Zn/SiO_2_ materials via H_2_ pretreatment (500 °C for 2 h). The intrinsic methanol formation rate of Cu–Zn(2)/SiO_2_ is 2.8 g h^–1^ g_Cu_^–1^, which is seven times higher than that of Cu/SiO_2_ (0.4 g h^–1^ g_Cu_^–1^, Figure 2a)^30^ leading overall to a significantly higher methanol selectivity for Cu–Zn(2)/SiO_2_ compared to Cu/SiO_2_ (79 vs 45%), as the CO formation rate in the two catalysts is similar. Cu–Zn(5)/SiO_2_ has an even higher intrinsic methanol formation rate and selectivity compared to Cu–Zn(2)/SiO_2_, i.e., 4.3 g h^–1^ g_Cu_^–1^ and 83%, respectively, with the space-time yield of 0.073 g h^–1^ g_cat_^–1^ at the contact time of 0.06 s g mL^–1^. This intrinsic methanol formation rate is considerably higher than methanol formation rates displayed by other related catalysts under similar conditions when rates are compared after normalization per mass of copper (Table S6). Based on previous reports, the addition of Zn to Cu-based methanol synthesis catalysts usually results in an increase of activity by ca. an order of magnitude.^10^ We observe a similar increase of activity, by ca. 11 times, when comparing Cu/SiO_2_ and Cu–Zn(5)/SiO_2_. However, in Cu–Zn(10)/SiO_2_, the intrinsic methanol formation rate and CO_2_ conversion decrease slightly compared to those in Cu–Zn(5)/SiO_2_ (Figures 2a and S35), possibly due to the coverage of surface Cu sites by Zn, as also indicated by the N_2_O titration results. Increasing the number of Zn ALD cycles [Cu–Zn(20)/SiO_2_] leads to a further decrease in the intrinsic methanol formation rate, i.e., to 3.6 g h^–1^ g_Cu_^–1^ at 87% selectivity to methanol (Figure S36). Note that catalysts prepared using the SOMC-ALD approach developed in this work require a comparatively small amount of Zn to yield the optimal activity, i.e., Cu:Zn = 5.25 at only 0.4 wt % Zn, a loading that is ca. 3 times lower than typically used in other Cu–Zn/SiO_2_ catalysts.^7,22^ Considering the remarkable influence of the addition of Zn to the activity and selectivity of Cu-based catalysts, such a low total amount of Zn is expected to contain significant quantities of active Zn sites (and, respectively, only a relatively low quantity of spectator Zn species). Developing materials that contain a high fraction of active Zn species is essential to facilitate in situ studies of the electronic state and speciation of active Zn species (vide infra).

Zn(5)/SiO_2_ (the respective reference material without Cu) shows only low activity and methanol selectivity (Figures S4 and S5). It is worth noting that the intrinsic CO formation rate of Cu–Zn(5)/SiO_2_ is similar to that of Cu/SiO_2_ or Zn(5)/SiO_2_, suggesting that the interaction of Cu and Zn does not influence the formation of CO considerably. When normalized by the mass of copper in the catalyst, the intrinsic methanol formation rate of Cu–Zn(5)/SiO_2_ exceeds that of Cu–ZnO–Al_2_O_3_ (0.7 g of h^–1^ g_Cu_^–1^, Figure S37).^30^ Comparing the intrinsic methanol formation rate normalized by the number of surface Cu^0^ sites (obtained from the N_2_O titration as discussed above) shows that the activity of Cu–Zn(5)/SiO_2_ is ca. ten times higher than that of Cu/SiO_2_ and slightly higher than that of Cu–ZnO–Al_2_O_3_ (10.6, 1.1, and 8.9 , Figure S38). At the same CO_2_ conversion of 1%, the methanol selectivity increases as follows: Cu/SiO_2_ < Cu–ZnO–Al_2_O_3_ < Cu–Zn(5)/SiO_2_ (32, 70, and 77%, respectively, Figure 2b).

Next, to evaluate how the addition of Zn influences the pathways of CO_2_ hydrogenation, we performed a contact time study by changing the flow rates of the reactants and comparing the results to the reference system Cu/SiO_2_. For Cu–Zn(5)/SiO_2_, the formation rate of methanol decreases with increasing contact time, i.e., from 3.5 to 1.6 g h^–1^ g_Cu_^–1^ for an increase in contact time from 0.06 to 0.4 s g mL^–1^ (Figure S36). However, this rate is still four times higher than that of Cu/SiO_2_ at an identical contact time of 0.4 s g mL^–1^.^30^ A similar decrease of the methanol formation rate with an increase in contact time was observed for other Cu-based catalysts and was attributed to the inhibition of methanol formation by the products, i.e., water and/or methanol.^5,29,30^ In contrast, the CO formation rate does not change significantly with an increase in contact time (Figure S36), consistent with distinct formation mechanisms for methanol and CO. In addition, the methanol formation rate does not change substantially with contact time for Cu/SiO_2_.^30^ These results indicate different active sites for methanol formation in Cu/SiO_2_ and Cu–Zn(5)/SiO_2_.

After more than 100 h of time on stream (TOS) of Cu–Zn(5)/SiO_2_, the formation rate of methanol decreased by 14% relative to the initial rate (Figure 2c), indicating a modest deactivation of the catalyst. Similar deactivation has also been observed in other Cu-based catalysts prepared by SOMC.^5,7^ Interestingly, the selectivity for CH_3_OH does not change substantially within 100 h (Figure S39).

A discussion of the catalytic performance of additional Cu–Zn/SiO_2_ catalysts, prepared using a lower H_2_ pretreatment temperature of 300 °C or a reversed order of how the metals are introduced onto the silica support, that is, first pulses of Et_2_Zn onto SiO_2–500_ followed by the grafting of copper mesityl and H_2_ pretreatment at 500 °C, or using pulses of Et_2_Zn directly onto Cu/SiO_2_, is provided in the Supporting Information file (Figures S1–S11). In brief, all of these additional materials feature a lower catalytic activity relative to Cu–Zn(5)/SiO_2_. We also note that the passivation of the Cu–Zn(2)–, Cu–Zn(5)–, and Cu–Zn(10)/SiO_2_ catalysts under 1% O_2_/N_2_ (2 h, room temperature) allows us to handle these materials in air without the loss of their catalytic performance (see Figures S12–S16 for details).

### Characterization of the Materials after Exposure to the Reaction Mixture

According to HAADF-STEM, the Cu particle size in Cu–Zn(5)/SiO_2_ increased from 1.8 ± 0.4 to 2.4 ± 0.6 nm after 100 h of TOS (Figure S40), and this likely accounts for the 14% decline in the methanol formation rate. No notable agglomeration of Zn was observed in the EDX maps (Figure S41), which is different from what was reported for the commercial Cu–ZnO–Al_2_O_3_ catalyst, for which the agglomeration/sintering of Zn phases has been suggested as one of the main reasons for deactivation.^13^ ICP-OES data showed that the Zn loading (0.4 wt %) remained unchanged after the 100 h stability test (Table S2).

**Figure 3:**
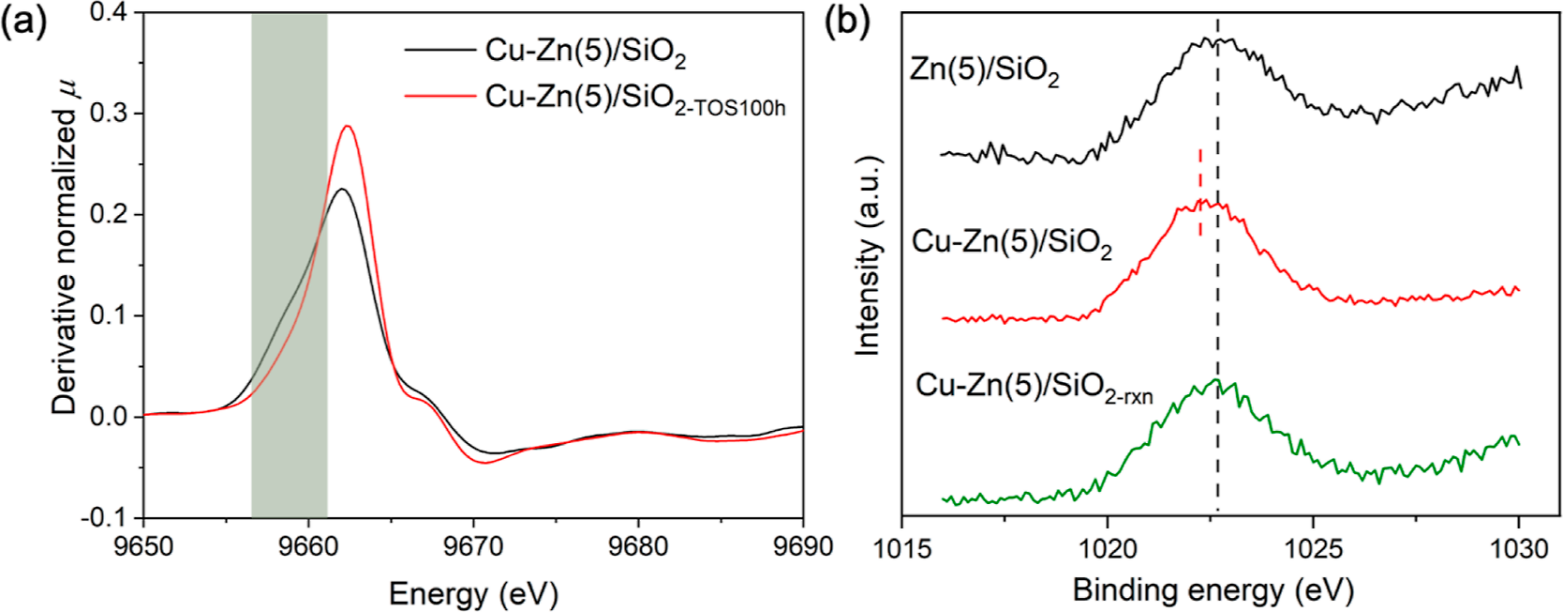
(a) First derivative of the Zn K-edge XANES of Cu–Zn(5)/SiO_2_ after H_2_ pretreatment at 500 °C (2 h) and after ca. 100 h of catalytic test. (b) Zn 2p_3/2_ XPS spectra of activated Zn(5)/SiO_2_ and Cu–Zn(5)/SiO_2_ (H_2_, 300 mbar, 1 h) and the pretreated Cu–Zn(5)/SiO_2_ after exposure to a reaction mixture of H_2_ and CO_2_ (300 and 100 mbar, respectively) at 230 °C for 1 h.

Ex situ XANES experiments were performed to compare Cu–Zn(5)/SiO_2_ after H_2_ pretreatment at 500 °C (2 h) and after 100 h of catalytic test. There is no obvious difference in the Cu K-edge XANES features of the active (working, cooled down in N_2_) catalyst and the activated (H_2_ pretreated) catalyst (Figure S42). However, the shoulder peak at 9658 eV in the Zn K-edge XANES derivative plot disappeared in Cu–Zn(5)/SiO_2_ that had been exposed to 100 h of TOS, suggesting that the CuZn NPs underwent dealloying under CO_2_ hydrogenation conditions (Figure 3a). This is similar to what has been reported for the Cu–ZnO–Al_2_O_3_ catalyst.^20^ Considering that the activity of Cu–Zn(5)/SiO_2_ after 100 h TOS is still remarkably high, this result implies that the CuZn alloy may not be the active and selective phase for the CO_2_ hydrogenation to methanol reaction (vide infra).^48^

XPS experiments were performed to further investigate the state of Zn under (i) reducing conditions, (ii) a CO_2_/H_2_ atmosphere, and (iii) methanol vapor. The measurements were performed by treating the passivated catalysts in a reaction chamber connected to the XPS instrument, which allows to study activated materials without their exposure to the ambient air.^49^ The Zn 2p_3/2_ XPS region of Cu–Zn(5)/SiO_2_ after pretreatment in H_2_ (300 mbar) at 200 °C (1 h) features a broad peak, indicating the presence of both Zn^0^ and Zn^2+^ states (Figure 3b). Peak deconvolution yields a Zn^0^/Zn^2+^ ratio of 0.45, which is higher than that of Zn(5)/SiO_2_ pretreated under identical conditions (Zn^0^/Zn^2+^ = 0.36, Figure S43). This observation is consistent with the partial formation of a CuZn alloy during reduction, as also indicated by the Zn K-edge XANES data discussed above. Next, these activated materials were exposed at 230 °C for 1 h to either a mixture of H_2_/CO_2_ = 3:1 (*P*_total_ = 400 mbar, Figure 3b) or to 120 mbar of methanol (Figure S44). Both experiments show that the Zn 2p_3/2_ peak of Cu–Zn(5)/SiO_2_ shifts to a higher energy. This result indicates the dealloying of the CuZn species and oxidation of Zn^0^ to Zn^2+^, consistent with the result of Zn XANES discussed above.

The Cu LMM Auger region of the XPS spectra provides information about the fractions of Cu^0^ and Cu^1+^ states in the catalysts. Reduction of passivated Cu/SiO_2_ or Cu–Zn(5)/SiO_2_ under 300 mbar of H_2_ at 100 or 200 °C for 1 h leads to a decrease of the fraction of Cu^1+^ and an increase in the fraction of Cu^0^ (Figures S45 and S46). Specifically, the Cu^1+^/Cu^0^ ratios (combined values obtained from fitting the two Cu^1+^ and two Cu^0^ peaks) in Cu/SiO_2_ and Cu–Zn(5)/SiO_2_ in the passivated materials are 0.72 and 0.82, respectively. This ratio decreases to 0.43 and 0.35 in Cu/SiO_2_ and Cu–Zn(5)/SiO_2_, respectively, after reductive treatment at 200 °C. The presence of Cu^1+^ sites can, at least in part, be attributed to interfacial Cu–O–Si≡ sites.^30^ After the exposure of the pretreated materials to a reaction mixture of H_2_ and CO_2_ (300 and 100 mbar, respectively) or to methanol vapor (120 mbar) at 230 °C for 1 h, the fraction of Cu^1+^ does not change notably, indicating that the Cu-oxidation states are stable under reactive conditions (Figures S47–S49).

### In Situ XAS Study during Pretreatment and CO_2_ Hydrogenation Conditions

To gain further insight into the structure of the Cu–Zn(5)/SiO_2_ catalyst under the reaction conditions, we turned to in situ XAS studies. Here, the passivated material was loaded into a capillary reactor and treated under a flow of H_2_ under conditions that are similar to those employed during the laboratory-based catalyst pretreatment (i.e., ramping up from room temperature to 300 °C under 1 bar of undiluted H_2_) before being exposed to the reaction conditions (details of the experiments are given in the Experimental part of the Supporting Information; the experimental profile is given in Figures S51 and S52). Analysis of the Cu K-edge XANES data before and after H_2_ pretreatment reveals that Cu is transformed from Cu^1+^ (edge energy of 8980.3 eV) to a reduced state (8979.0 eV, Figure S53). The reduction of Cu is further supported by EXAFS data (its fitting is discussed in Supporting Information), although EXAFS analysis cannot exclude the presence of a minor amount of Cu^0^ in passivated Cu–Zn(5)/SiO_2_ prior to in situ H_2_ treatment.^50,51^ Summarizing, in situ Cu K-edge XAS data are consistent with the formation of the Cu^0^ state in the activated Cu–Zn(5)/SiO_2_.

**Figure 4:**
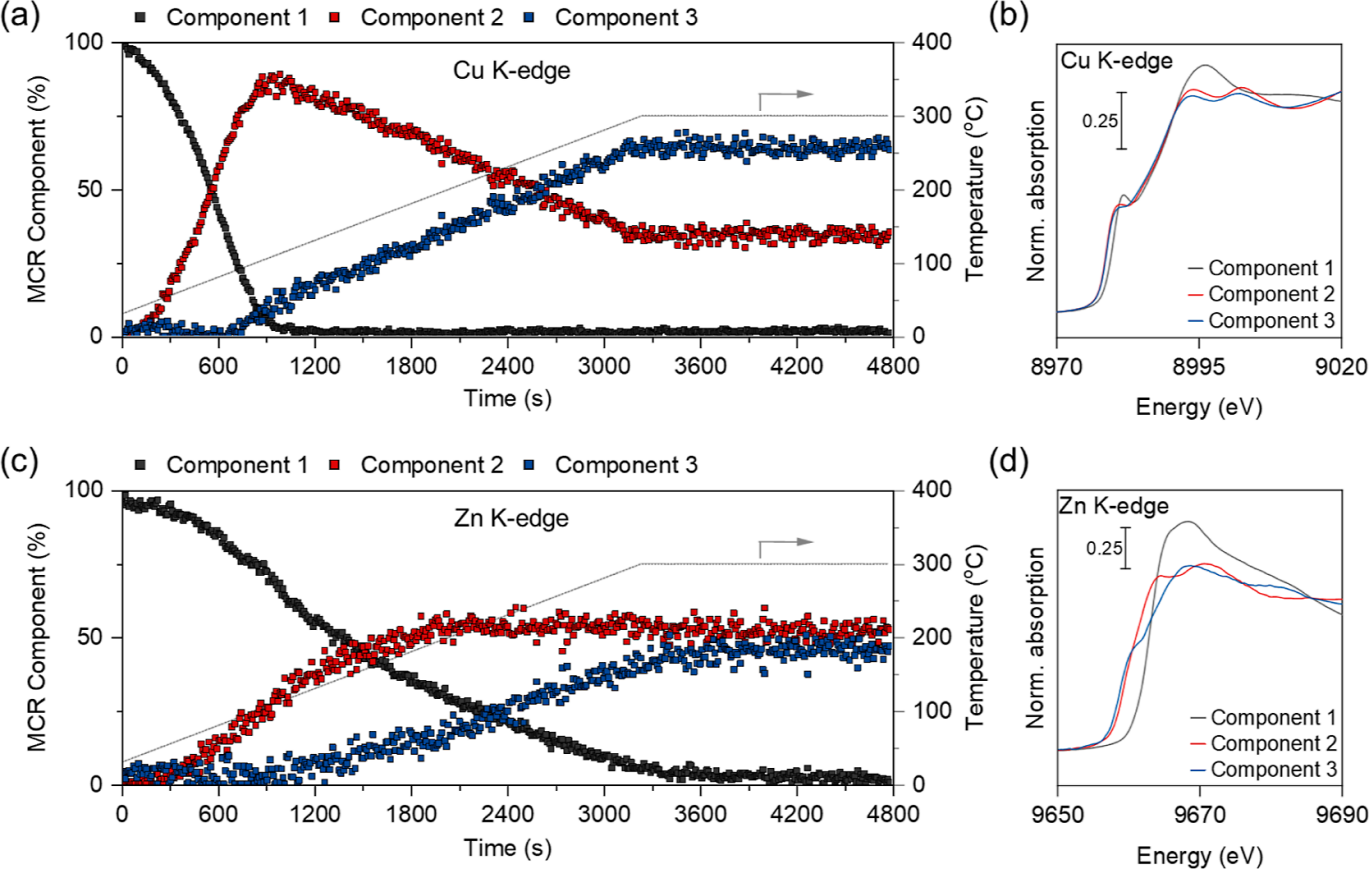
MCR-ALS analysis of XANES data recorded during H_2_ pretreatment of passivated Cu–Zn(5)/SiO_2_ with (a) extracted component fractions at the Cu K-edge as a function of time; gray trace shows temperature as a function of time; and (b) the corresponding spectrally pure components (see Figure S53 for in situ spectra). (c) Component fractions at the Zn K-edge as a function of time; gray trace shows temperature as a function of time; and (d) corresponding spectrally pure components (see Figure S57 for in situ spectra). Conditions: 3 mm quartz capillary (i.d. 2.8 mm), using ca. 20 mg of passivated Cu–Zn(5)/SiO_2_, temperature raised from room temperature to 300 °C, *P* = 1 bar, total flow rate 10 mL min^–1^ of indiluted H_2_.

The simultaneous monitoring of XANES spectra at the Cu and Zn K-edges during hydrogen pretreatment reveals that the reduction of Cu occurs rapidly with an increase in temperature and that the reduction of Zn requires higher temperatures than the reduction of Cu (Figures S53 and S57). A more detailed interrogation of this process was performed by a multivariate curve resolution alternating least-squares (MCR-ALS) analysis of the XANES data. MCR-ALS analysis allows for a blind-source (i.e., without references) separation of kinetically unique spectral features.^52^ MCR-ALS analysis (of the Cu K-edge XANES data) shows that the reduction process is best described by using a three-component fit (Figure 4a,b). The onset of the reduction process from Cu^1+^ to Cu^0^ in Cu–Zn(5)/SiO_2_ is initiated at a relatively low temperature (at ca. 50 °C), as evidenced by the rapid depletion of component 1 in the MCR-ALS fit. Concomitantly, a new component emerges (denoted component 2), which is subsequently depleted above ca. 100 °C, transforming into component 3. Component 1 corresponds to Cu^1+^ species with a pre-edge feature, a white line region (intensity and profile), and edge energy reminiscent of Cu_2_O (yet a minor amount of Cu^0^ may be present, as mentioned above). Both component 2 and component 3 are consistent with Cu^0^ species, as evidenced by the characteristic edge profile and the white line doublet at around 9000 eV.^50,51^ Components 2 and 3 differ slightly in the intensity of the white line region, with component 3 having a doublet feature of lower intensity and a more smoothly rising edge profile, indicative of either an increased structural disorder that might arise from thermal effects or alloying; the latter explanation is consistent with the MCR-ALS analysis of the Zn K-edge data discussed below.

MCR-ALS analysis of the Zn K-edge XANES data during H_2_ pretreatment also yields the presence of three kinetically distinct components, i.e., one component representing Zn^2+^ species and two other components representing reduced Zn species. With increasing temperature, component 1 is continuously depleted, while component 2 emerges starting from ca. 85 °C (Figure 4c,d). At ca. 125 °C, component 3 is observed. In contrast to the results of the Cu K-edge XANES data, the fraction of component 2 remains nearly constant above 200 °C, with the fraction of component 3 rising, presumably due to the depletion of component 1. An alternative explanation for the rising relative fraction of component 3 above ca. 200 °C is the similar rate for formation of component 2 from component 1 and depletion of component 2 to component 3. The spectral profile of component 1 is consistent with a Zn^2+^ species that is, however, distinct from crystalline ZnO, ZnO/SiO_2_, or an ordered Zn^2+^ silicate.^53–56^ Therefore, component 1 is assigned to amorphous zinc oxide (ZnO_*x*_) that is highly dispersed on the silica support. Both components 2 and 3 correspond to reduced Zn species, i.e., metallic Zn species, with component 2 possessing a more distinct increase of the edge profile relative to component 3. Component 3 can be assigned to a CuZn alloy, while component 2 is assigned to nanolloyed Zn^0^.^7,19,22^ Therefore, the evolution of the fractions of components 1–3 is consistent with the reduction of Zn^2+^ species to Zn^0^ species, possibly facilitated by Cu, which is followed by a partial intercalation of the formed Zn^0^ into Cu^0^, yielding a CuZn alloy.

To summarize, applying in situ XAS analysis at the Cu and Zn K-edges yields the following conclusions concerning the reduction of passivated Cu–Zn(5)/SiO_2_: (a) the passivated catalyst contains predominantly Cu^1+^ and Zn^2+^ species (amorphous ZnO_*x*_), which are reduced during H_2_ pretreatment; (b) Cu^1+^ is reduced to Cu^0^ at a lower temperature than the temperature that is required for the reduction of Zn^2+^; (c) the temporal dynamics of the fraction of component 3 (both for Cu and Zn) in the material are correlated (Figure 4a,c), implying that component 3 is an alloy of Cu and Zn; (d) the activated catalyst contains Cu and Zn predominantly in metallic states, i.e., as a CuZn alloy as well as some not alloyed Cu^0^ and Zn^0^. This analysis indicates that Cu and Zn interact intimately in passivated Cu–Zn(5)/SiO_2_, an advantage arising from the utilized SOMC-ALD methodology. It is essential to note that while the ex situ XANES spectrum of Cu–Zn(5)/SiO_2_, obtained after H_2_ pretreatment at 500 °C, shows a mixture of Zn states, i.e., Zn^0^ and Zn^2+^, the in situ XANES experiment shows clearly that under a H_2_ atmosphere, Zn^2+^ species are reduced fully to Zn^0^ even at 300 °C (Figures 1f and 4c). This observation is consistent with the emergence of reactive metal support interactions (RMSI) in Cu–Zn(5)/SiO_2_ under a H_2_ atmosphere,^57^ likely due to an atomic-scale mixing, as was reported recently for an ALD-derived PdGa/Al_2_O_3_ catalyst for CO_2_ hydrogenation to methanol.^41^ The emergence of RMSI in a H_2_ atmosphere is also consistent with the XPS results discussed above.

**Figure 5:**
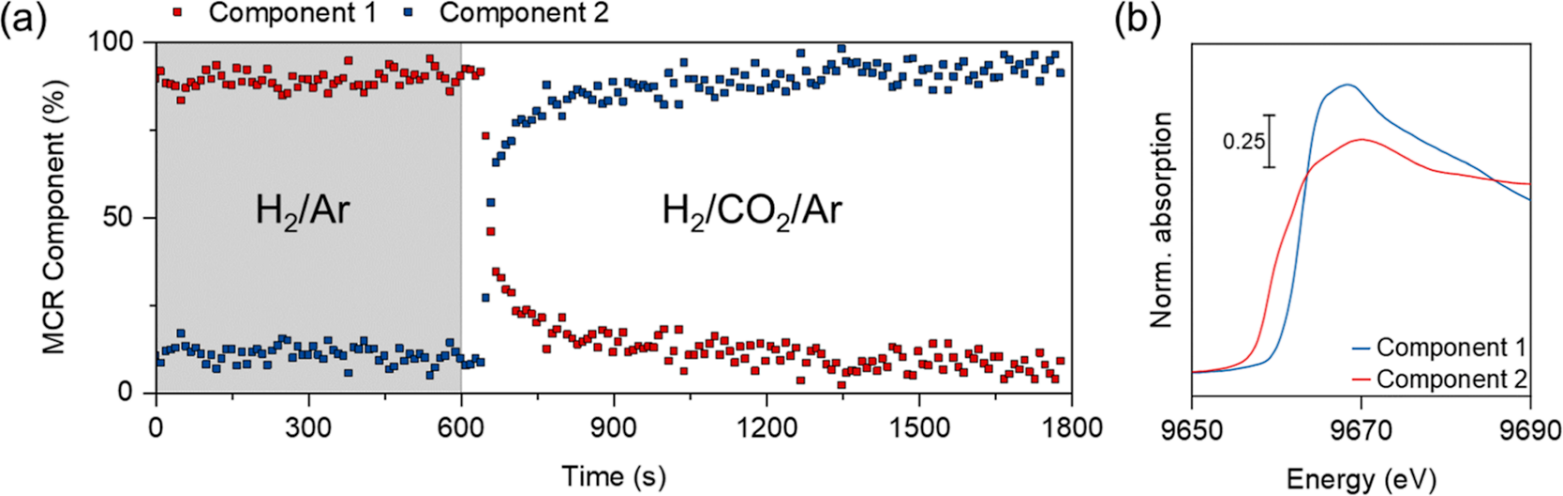
MCR-ALS analysis of the XANES of Cu–Zn(5)/SiO_2_ acquired at the Zn K-edge under H_2_/Ar and upon exposure to CO_2_ hydrogenation conditions. (a) Fractional contribution of the identified components as a function of time; the gray shaded region denotes period prior to the introduction of CO_2_ to the H_2_/Ar mixture. (b) Spectrally pure components as obtained by MCR-ALS analysis. Reaction conditions: 3 mm quartz capillary (i.d. 2.8 mm), ca. 20 mg catalyst bed, switching from H_2_/Ar (3:2) to H_2_/CO_2_/Ar (3:1:1), *T* = 230 °C, *P* = 11 bar, and total flow rate = 10 mL min^–1^.

To gain further insight into the interplay between Cu and Zn, changes in their electronic states and speciation upon exposure to the CO_2_ hydrogenation conditions were investigated. In this set of experiments, a flow of H_2_/Ar (3:2) was switched to H_2_/Ar/CO_2_ (3:1:1) at 11 bar, while structural changes were recorded simultaneously (see Supporting Information for details). XANES spectra at the Cu K-edge obtained before and after the switch to the gas atmosphere show only minor changes (Figure S58). Consistent with this observation, the Cu–M CNs obtained from fitting of Cu K-edge EXAFS data both before and after the gas switch are indistinguishable, suggesting that Cu remains metallic and the size of the Cu structures (NPs) does not change appreciably (Figure S59 and Table S7). In contrast, a pronounced change in the XANES spectra at the Zn K-edge is observed upon a change of the gas atmosphere, consistent with the predominant oxidation of Zn^0^ to Zn^2+^ (Figure S60).^22^ A more detailed analysis of changes in the Zn K-edge XANES spectra due to gas switching was performed by using MCR-ALS analysis. The results reveal that exposure of activated Cu–Zn(5)/SiO_2_ to a CO_2_ hydrogenation gas atmosphere leads to a nearly instantaneous oxidation of Zn, i.e., the Zn in the reduced state (represented for the purpose of this analysis by a single reduced component 1) is oxidized to component 2 within several minutes at 230 °C (Figure 5). Component 2 is reminiscent of amorphous ZnO_*x*_ dispersed on SiO_2_.^56^ Overall, the in situ XANES data suggest that the majority of Zn^0^ that is present in the form of a CuZn alloy (or as separate Zn^0^ species) in the activated catalyst (formed during H_2_ pretreatment) is rapidly oxidized to Zn^2+^ upon exposure to CO_2_ hydrogenation conditions. This observation is consistent with previous reports on similar bimetallic Cu–Zn systems.^20,22,58^

Lastly, we performed multiple gas switching experiments by alternating the flow between H_2_/Ar (3:2) and H_2_/CO_2_/Ar (3:1:1) at 230 °C and 11 bar pressure to detect dynamic changes in XANES features at the Zn K-edge. A rapid reversible alloying-dealloying pattern is observed, that is, alloying under H_2_ and dealloying under a reaction atmosphere, as presented in Figure S61.

### Characterization of Surface Intermediates by Solid-State NMR

To assess the nature of surface species that form under CO_2_ hydrogenation conditions, we utilized solid-state nuclear magnetic resonance (NMR) spectroscopy. Note that owing to the Knight shift that broadens peaks arising from intermediates absorbed on the metallic (i.e., conductive) surface of Cu^0^ or CuZn-alloy, NMR studies provide information related to species stabilized on the support or the particle/support interface.^5^ Toward this end, we treated Cu–Zn(5)/SiO_2_ at 230 °C (12 h) with a 3:1 mixture of ^13^C-labeled CO_2_ and H_2_ (5 bar) and subsequently removed volatiles at room temperature (ca. 10^–5^ mbar). Results indicate the presence of surface methoxy species in Cu–Zn(5)/SiO_2_, identified by a peak in the ^13^C CP-MAS spectrum at ca. 49 ppm (Figure S62).^5^ A peak at the same chemical shift is also observed in the spectrum of Cu/SiO_2_, and both ^13^C peaks show a correlation in the ^1^H–^13^C HETCOR spectrum with the ^1^H peak at 3.84 ppm (Figures S63 and S64). No formate peaks that would be expected at the ^13^C chemical shift of ca. 168 ppm are observed in any of the two catalysts.^5,30,59^

### Temporal Evolution of Surface Intermediates during CO_2_ Hydrogenation

The formation and presence of surface intermediate species during the CO_2_ hydrogenation conditions were investigated further by operando DRIFTS at 230 °C and 20 bar (Figure S65). After pretreatment in H_2_ (500 °C, 2 h) and purging with He, the gas atmosphere was switched to a mixture of CO_2_/H_2_ (1:3). The evolving surface species were studied by MCR-ALS and correlated with the gaseous products as sampled by mass spectroscopy (MS).^52^ The assignment of the IR bands and the corresponding vibrational frequencies is given in Table S8.

**Figure 6:**
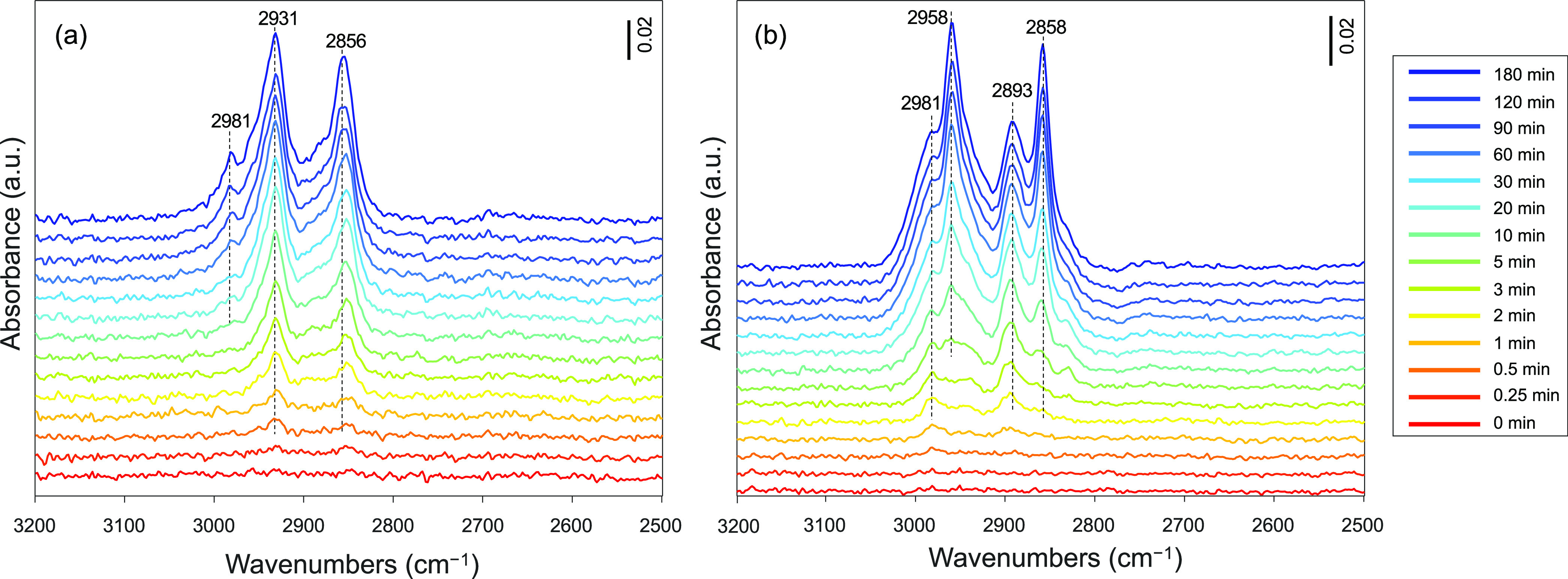
Time-resolved operando DRIFT spectra of the surface species formed under CO_2_ hydrogenation conditions over (a) Cu/SiO_2_ and (b) Cu–Zn(5)/SiO_2_ catalysts. Pretreatment was performed at 500 °C under 20 mL min^–1^ of H_2_ for 2 h. Reaction conditions: ca. 30 mg of catalyst, 230 °C, 20 bar, H_2_/CO_2_ = 3:1, total flow rate 20 mL min^–1^.

At steady-state, the surface adsorbates saturate the catalyst surface (Figure 6). The main surface species on Cu/SiO_2_ are μ-HCOO*(Cu) at 2856 and 2931 cm^–1^ and surface-bound methanol CH_3_OH* at 2981 cm^–1^ (Figure 6a). The formation of CH_3_OH* is delayed by up to 20 min relative to the formation of the μ-HCOO*(Cu). In contrast, at steady-state, the key surface species on Cu–Zn(5)/SiO_2_ are μ-HCOO*(ZnO_*x*_) at 2893 cm^–1^,^60,61^ where ZnO_*x*_ is an amorphous ZnO species dispersed on silica, as discussed above. In addition, CH_3_O*(SiO_2_) featuring bands at 2858 and 2958 cm^–1^ and CH_3_OH* at 2981 cm^–1^ are also observed (Figure 6b). Owing to the overlapping bands in the C–H region, the apparent absence of CH_3_O*(SiO_2_) on Cu/SiO_2_ and μ-HCOO*(Cu) on Cu–Zn(5)/SiO_2_ requires validation by MCR-ALS analysis, as discussed below.

As the bands assigned to ν(C–H) of μ-HCOO*(Cu) and to CH_3_O*(Cu) tend to overlap, their assignment also requires consideration of ν(C–O). However, both Cu/SiO_2_ and Cu–Zn(5)/SiO_2_ provide a low infrared throughput below 2000 cm^–1^, especially Cu/SiO_2_ (Figure S66), which hampers the detection of the characteristic ν(C–O) band of μ-HCOO*(Cu). Yet, dilution of the catalysts with SiO_2_ slightly increases the infrared throughput below 2000 cm^–1^ and allows us to discern the band at 1604 cm^–1^ that can be assigned to ν(C–O) of μ-HCOO*(Cu) (Figure S67). Likewise, characteristic ν(C–O) of μ-HCOO*(ZnO_*x*_) is observed at ca. 1590 cm^–1^ in the case of Cu–Zn(5)/SiO_2_.^60^ Carbonyl species (CO*) were not detected on both catalysts (the bands in the 2250–2050 cm^–1^ region are assigned to combination bands of pressurized CO_2_ rather than to bands due to bound CO, Figure S68).^62^

**Figure 7:**
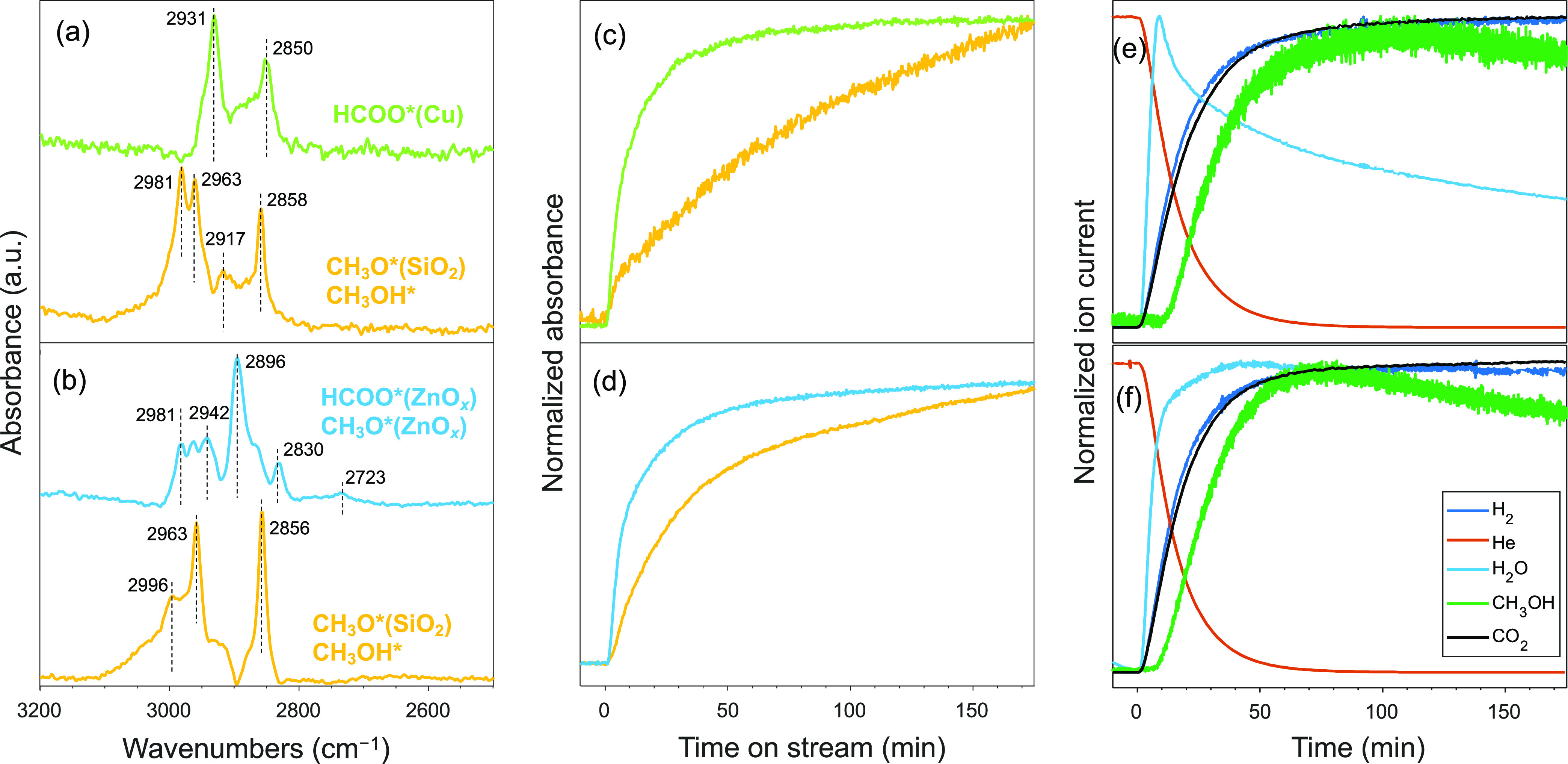
Time-resolved operando DRIFTS under CO_2_ hydrogenation conditions. Spectra of the different components obtained by MCR-ALS analysis were applied to time-resolved DRIFT spectra over (a) Cu/SiO_2_ and (b) Cu–Zn(5)/SiO_2_ catalysts. Relative fraction profiles of the spectra of the corresponding components obtained by MCR-ALS were obtained for (c) Cu/SiO_2_ and (d) Cu–Zn(5)/SiO_2_ catalysts. Corresponding normalized ion current signals were obtained from mass spectrometry of (e) Cu/SiO_2_ and (f) Cu–Zn(5)/SiO_2_ catalysts. Pretreatment was performed at 500 °C under 20 mL min^–1^ of H_2_ for 2 h. Reaction conditions: ca. 30 mg catalyst, 230 °C, 20 bar, H_2_/CO_2_ = 3:1, total flow rate 20 mL min^–1^.

MCR-ALS analysis identified two kinetically separable spectra for Cu/SiO_2_ and Cu–Zn(5)/SiO_2_ (Figure 7a,b). The respective temporal evolution of each spectrum is presented in Figure 7c,d. On Cu/SiO_2_, μ-HCOO*(Cu) formed rapidly during CO_2_ hydrogenation conditions, followed by a slower formation of CH_3_O*(SiO_2_) and CH_3_OH* (Figure 7a,b). The latter methoxy/methanol species appear simultaneously in the MCR-ALS analysis. This implies that their formation is kinetically indistinguishable (i.e., appearing at the same time) within the time-scale of this study. Similarly, on Cu–Zn(5)/SiO_2_, first μ-HCOO*(ZnO_*x*_) species form, followed by the gradual formation of CH_3_O*(SiO_2_) and CH_3_OH*. Interestingly, the rapidly formed μ-HCOO*(ZnO_*x*_) species are accompanied by the formation of kinetically identical CH_3_O*(ZnO_*x*_) species. Since the hydrogenation of surface formate species has been discussed as the rate-limiting step in the methanol synthesis over Cu-based catalysts (although the rate-limiting step may change to methanol desorption at high CO_2_ conversion),^63,64^ the observation of kinetically favored methoxy species in Cu–Zn(5)/SiO_2_ implies a critical role of Zn in the catalytic cycle. It is noteworthy that the band due to μ-HCOO*(Cu) is not observed in our Cu–Zn(5)/SiO_2_ catalyst, which is in contrast to other Cu–Zn-based catalysts.^48,65–67^

The effluent gas was analyzed during the DRIFTS experiment by MS. A significant delay in the CH_3_OH signal was observed as compared to H_2_O, which correlates with the delay in the formation of CH_3_O*(SiO_2_) species. This may imply that the formed CH_3_OH reacts with silanol groups of SiO_2_ to form CH_3_O*(SiO_2_).^68^ The delay in the appearance of the product methanol, exhibiting adsorption-breakthrough behavior (green, Figure 7e,f), hints at a strong interaction or reaction of methanol with the catalyst surface. CH_3_O*(SiO_2_) species are expected to be stable and do not participate in the CO_2_ hydrogenation reaction.^68^

Transient experiments were performed to distinguish catalytically active species from spectator species by driving the catalyst away from its steady-state regime, where all surface species, including spectators, can be present as major species. A short exposure of the catalytic surface to CO_2_ or H_2_ allows for active species to form. After pretreatment with H_2_, the catalyst was first kept under H_2_/He (3:1) for 30 min prior to switching to He/CO_2_ (3:1), leading to the CO_2_ activation and the formation of surface species. The surface species varied in every CO_2_ and H_2_ switching cycle and required up to 3 cycles to reach a quasi-steady state (i.e., reproducible response to transient conditions) surface and gas concentration. The subsequent 3–4 cycles of IR responses were averaged to improve the signal-to-noise ratio and were analyzed by MCR-ALS analysis. The surface plots of the averaged cycles are shown in Figure S69.

**Figure 8:**
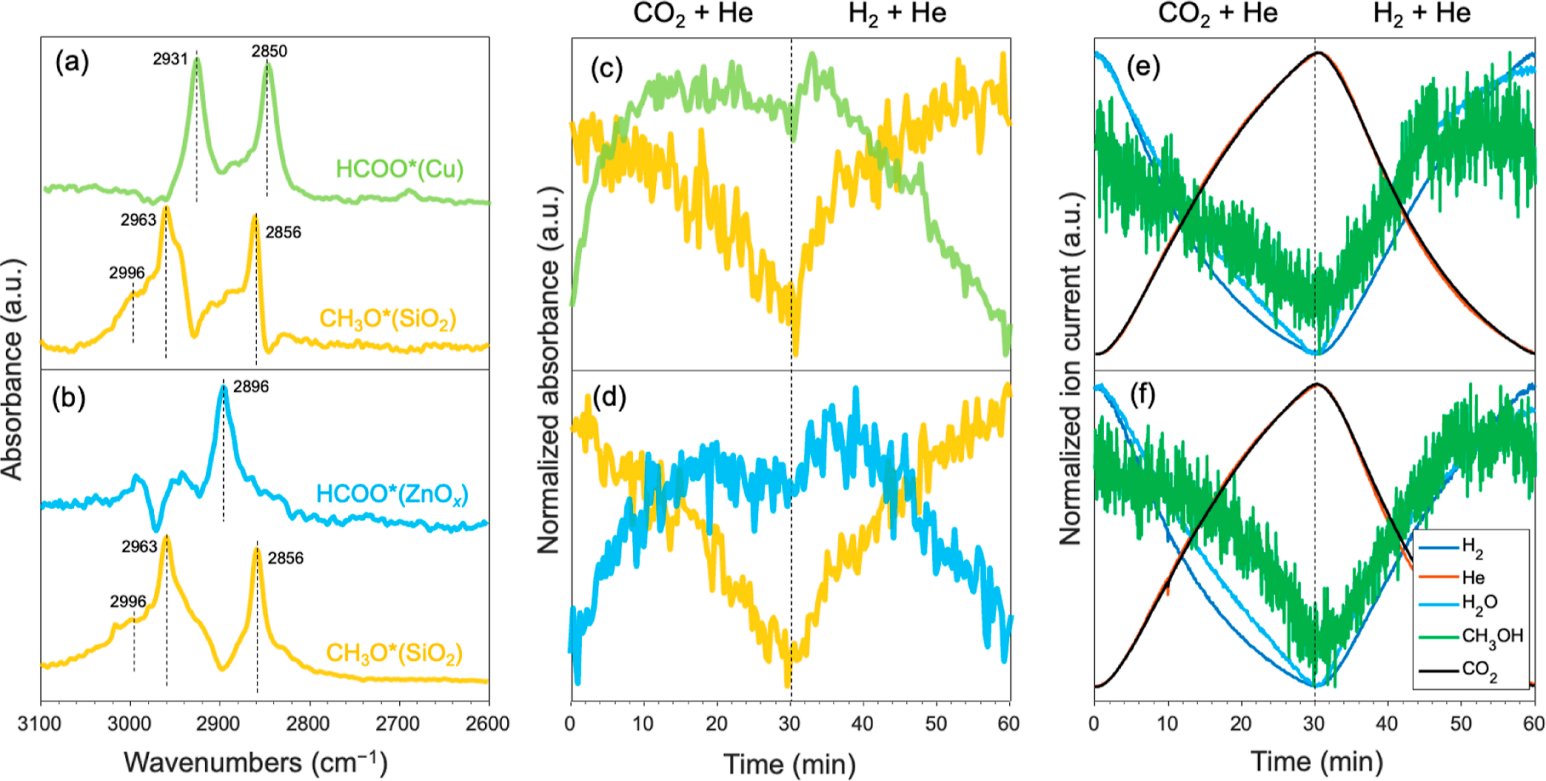
Time-resolved operando DRIFTS under transient concentration perturbation conditions using the CO_2_/He and He/H_2_. Spectra of the components were obtained by MCR-ALD analysis of the time-resolved DRIFT spectra over (a) Cu/SiO_2_ and (b) Cu–Zn(5)/SiO_2_ catalysts. Relative fraction profiles of the spectra of the corresponding components obtained by MCR-ALS from (c) the Cu/SiO_2_ and (d) the Cu–Zn(5)/SiO_2_ catalysts. The corresponding normalized ion current signal was obtained from simultaneously acquired mass spectrometry data of (e) Cu/SiO_2_ and (f) Cu–Zn(5)/SiO_2_ catalysts. Condition: pretreatment at 500 °C under 20 mL min^–1^ of H_2_ for 2 h using ca. 30 mg of catalyst followed by He/CO_2_ = 3:1 and H_2_/He = 3:1, *T* = 230 °C, *P* = 20 bar, total flow rate 20 mL min^–1^.

The MCR-resolved spectra of the transient experiment contained fewer overlapping features compared to the steady-state experiment, as shown in Figure 8a,b for Cu/SiO_2_ and Cu–Zn(5)/SiO_2_, respectively. This, and also the methanol formation under transient conditions, may indicate that species undetected under the conditions of the transient experiment are spectator species, e.g., CH_3_O*(ZnO_*x*_). Alternatively, and probably more importantly, CH_3_O*(ZnO_*x*_) is not a stable intermediate under transient conditions, and it can be transformed to adsorbed methanol or methoxy on SiO_2_. A significantly lower intensity of CH_3_OH* was observed in the transient experiment relative to the steady-state conditions, indicative of lower CH_3_OH* coverage in the transient experiment. Under a CO_2_ atmosphere, CO_2_ reacted with chemisorbed H_2_ species to form μ-HCOO* as the common intermediate on both Cu/SiO_2_ and Cu–Zn(5)/SiO_2_ catalysts. However, the key difference relates to the adsorption site of μ-HCOO*, i.e., μ-HCOO* adsorbs on Cu using Cu/SiO_2_ and on ZnO_*x*_ using Cu–Zn(5)/SiO_2_ (Figure 8c,d). The fact that Cu–Zn(5)/SiO_2_ features μ-HCOO*(ZnO_*x*_) species but no μ-HCOO*(Cu) species highlights the role of Zn in the enhanced activity of the Cu–Zn(5)/SiO_2_ catalyst relative to Cu/SiO_2_. Both formate μ-HCOO*(Cu or ZnO_*x*_) species reach a plateau in the CO_2_ atmosphere, and their concentration increases only slightly after switching to a H_2_ atmosphere [in both Cu/SiO_2_ and Cu–Zn(5)/SiO_2_, as shown in Figure 8c,d, respectively]. On the other hand, gas switching influences the CH_3_O*(SiO_2_) species similarly on both Cu/SiO_2_ and Cu–Zn(5)/SiO_2_, that is, CH_3_O*(SiO_2_) species disappear under a CO_2_ atmosphere and appear under a H_2_ atmosphere. Noteworthy, the signal of CH_3_OH detected by MS closely follows the temporal profile of the CH_3_O*(SiO_2_) species (Figure 8e,f). Therefore, the transient experiments show that CH_3_O*(SiO_2_) species desorb under a CO_2_ atmosphere and reform by adsorption of CH_3_OH under a H_2_ atmosphere.

Overall, our operando DRIFTS studies show that the role of Zn^2+^ sites in ZnO_*x*_ is to stabilize formate species and accelerate their hydrogenation to methoxy species while still being bound to ZnO_*x*_. Methoxy bound to ZnO_*x*_ is significantly less stable under CO_2_ hydrogenation conditions than methanol or methoxy bound to SiO_2_, thereby accelerating methanol formation.

## Conclusions

We have developed a highly active, selective, and stable Cu–Zn/SiO_2_ catalyst through a combination of SOMC and ALD, showing an intrinsic methanol formation rate of 4.3 g h^–1^ g_Cu_^–1^ and selectivity of 83% during CO_2_ hydrogenation at 25 bar. This SOMC-ALD approach provides an atomic-scale mixing of Cu and Zn species and thus enables a strong interaction (alloying) between Cu and Zn, achieved using a low loading of Zn (0.4 wt % in the optimized catalyst). The low loading of Zn and the atomic-scale mixing between Cu and Zn minimized the amount of spectator Zn sites, which facilitates the identification of changes in the electronic states and speciation of Zn that are related to the active site formation under reactive conditions. In situ XAS as well as XPS studies reveal that while the CuZn alloy forms in H_2_ pretreatment conditions, dealloying occurs rapidly under CO_2_ hydrogenation conditions, and the alloy evolves into an active Cu^0^–Zn^2+^ interface. Consequently, the Cu–Zn^2+^ interface is responsible for the superior catalytic performance due to the faster hydrogenation rate of the μ-HCOO*(ZnO_*x*_) species compared to the μ-HCOO*(Cu) species during the CO_2_ hydrogenation reaction, as identified by operando DRIFTS.

## Supporting Information Available

Details about experimental procedures, spectroscopic methods, and associated data (PDF)
