# Supplementary material for: Combining Atomic Layer Deposition with Surface Organometallic Chemistry to Enhance Atomic-Scale Interactions and Improve the Activity and Selectivity of Cu–Zn/SiO2 Catalysts for the Hydrogenation of CO2 to Methanol
Source: JACS Au. 2023 Aug 23;3(9):2536–49. doi: 10.1021/jacsau.3c00319 (PMC10523371; doi:10.1021/jacsau.3c00319)
Supplement: Supplementary file 1 — au3c00319_si_001.pdf [file au3c00319_si_001.pdf]

Supplementary Information

Combining Atomic Layer Deposition with Surface Organometallic Chemistry to Enhance Atomic-Scale Interactions and Improve Activity and Selectivity of Cu-Zn/SiO_2 Catalysts for the Hydrogenation of CO_2 to Methanol

Hui Zhou,[1,2] Scott R. Docherty,[3] Nat Phongprueksathat,[4] Zixuan Chen,[1] Andrey V. Bukhtiyarov,[5] Igor P. Prosvirin,[6] Olga V. Safonova,[7] Atsushi Urakawa,[4*] Christophe Copéret,[3*] Christoph R. Müller,[1*] and Alexey Fedorov[1*]

[1] Department of Mechanical and Process Engineering, ETH Zürich, CH-8092 Zürich, Switzerland

[2] Department of Energy and Power Engineering, Tsinghua University, 100084 Beijing, China

[3] Department of Chemistry and Applied Biosciences, ETH Zürich, CH-8093 Zürich, Switzerland

[4] Department of Chemical Engineering, Delft University of Technology, 2629 HZ Delft, The Netherlands

[5] Synchrotron Radiation Facility SKIF, Boreskov Institute of Catalysis SB RAS, 630559 Kol'tsovo, Russia

[6] Boreskov Institute of Catalysis, SB RAS, 630090 Novosibirsk, Russia

[7] Paul Scherrer Institute, CH-5232 Villingen, Switzerland

E-Mail:

A.Urakawa@tudelft.nl

ccoperet@ethz.ch

muelchri@ethz.ch

fedoroal@ethz.ch

Table of Content

Experimental part 7

Supplementary Figures 11

Figure S1. Formation rates normalized per mass of Cu of Cu-Zn(5)/SiO_2 catalysts after different H_2 treatment temperatures together with their selectivities for CH_3OH, specified above the respective bars (230 °C, 25 bar, $H_2/CO_2/N_2$ = 3:1:1, contact time 0.06 s g mL^{-1}). 11

Figure S2. A representative TEM image and particle size distribution of Cu-Zn(5)/SiO_{2-300}. (a) HAADF-STEM (scale bar: 20 nm). (b) Particle size distribution. 12

Figure S3. H_2 temperature-programmed desorption of the catalysts after saturation in 5% H_2/Ar. 12

Figure S4. Intrinsic formation rates of Zn(5)/SiO_2, Zn(5)-Cu/SiO_2, and Cu-Zn(5)/SiO_2 obtained by extrapolation to zero conversion (zero contact time) together with the respective selectivities for CH_3OH, specified above the respective bars (230 °C, 25 bar, $H_2/CO_2/N_2$ = 3:1:1). 13

Figure S5. Formation rates of CH_3OH and CO for Zn(5)/SiO_2 and Zn(5)-Cu/SiO_2 with respect to the contact time extrapolated to zero conversion (zero contact time) using second-order polynomial fits. .13

Figure S6. TEM-EDX of Zn(5)-Cu/SiO_2. Scale bar: 6 nm. 14

Figure S7. TEM and particle size of Zn(5)-Cu/SiO_2. (a) HAADF-STEM (scale bar: 20 nm). (b) Particle size distribution. 14

Figure S8. TEM and particle size distribution of Cu_{red}-Zn(5)/SiO_2. (a) HAADF-STEM (scale bar: 20 nm). (b) Particle size distribution. 15

Figure S9. TEM-EDX maps of Cu_{red}-Zn(5)/SiO_2. Scale bar: 6 nm. 15

Figure S10. Intrinsic formation rates normalized per mass of Cu of Cu-Zn(5)/SiO_2 and Cu_{red}-Zn(5)/SiO_2 obtained by extrapolation to zero conversion (zero contact time, Figure S11) together with their respective selectivities for CH_3OH, specified above the respective bars (230 °C, 25 bar, $H_2/CO_2/N_2$ = 3/1/1). 16

Figure S11. Formation rates of CH_3OH and CO for Cu_{red}-Zn(5)/SiO_2 with respect to the contact time; extrapolated to zero conversion (zero contact time) using second-order polynomial fits. 16

Figure S12. Images of Cu-Zn(5)/SiO_2 catalysts after different pretreatments: (a) CuMes-Et_2Zn(5)/SiO_2 without air exposure. (b) CuMes-Et_2Zn(5)/SiO_2 after air exposure. (c) CuMes-Et_2Zn(5)/SiO_2 after reduction (H_2, 500 °C, 2 h), passivation (1% O_2/N_2, room temperature, 2 h), and air exposure. 17

Figure S13. Formation rates normalized per mass of Cu of Cu-Zn(5)/SiO_2 after different pretreatment procedures together with the selectivities for CH_3OH, specified above the respective bars (230 °C, 25 bar, $H_2/CO_2/N_2$ = 3:1:1, contact time 0.06 s g mL^{-1}). Air-free: the catalyst was loaded inside a glovebox with exposure to air. Exposed to air: the catalyst was loaded in air. Reduced before exposure to air: the catalyst was reduced (H_2, 500 °C, 2 h) and then loaded in air. Passivated: the catalyst was reduced (H_2, 500 °C, 2 h), passivated (1% O_2/N_2, room temperature, 2 h), and loaded in air. All the catalysts were in-situ reduced under H_2 (500 °C, 2 h) before CO_2 hydrogenation. 18

Figure S14. A representative TEM image and particle size distribution of Cu-Zn(5)/$SiO_{2\text{-air}}$. (a) HAADF-STEM (scale bar: 20 nm). (b) Particle size distribution. 18

Figure S15. TEM-EDX of Cu-Zn(5)/$SiO_{2\text{-air}}$. Scale bar: 6 nm. 19

Figure S16. Temperature-programmed reduction of passivated Cu-Zn(5)/SiO_2 (1% O_2/N_2, 2 h) using 5% H_2/Ar........ 19

Figure S17. Transmission IR spectra of $SiO_{2\text{–}500}$, Et_2Zn(5)/SiO_2, and Zn(5)/SiO_2........ 20

Figure S18. A representative TEM image and particle size distribution of CuMes-Et_2Zn(5)/SiO_2. (a) HAADF-STEM (scale bar: 20 nm). (b) Particle size distribution. 20

Figure S19. TEM-EDX maps of CuMes-Et_2Zn(5)/SiO_2. Scale bar: 3 nm........ 21

Figure S20. HAADF-STEM image of CuMes/SiO_2. Scale bar: 20 nm........ 21

Figure S21. TEM-EDX maps of CuMes/SiO_2. Scale bar: 6 nm. 22

Figure S22. Cu and Zn loading in CuMes-Et_2Zn(*n*)/SiO_2 and Cu-Zn(*n*)/SiO_2 (*n* = 2, 5, 10). 22

Figure S23. TEM of Cu-Zn(5)/SiO_2. (a) BF-TEM. (b) STEM-HAADF. Scale bar: 10 nm........ 23

Figure S24. A representative TEM image and particle size of Cu/SiO_2. (a) HAADF-STEM (scale bar: 20 nm). (b) Particle size distribution. 23

Figure S25. TEM-EDX maps of Cu-Zn(5)/SiO_2. Scale bar: 10 nm. 24

Figure S26. XRD pattern of Cu-Zn(5)/SiO_2. 24

Figure S27. TEM of Zn(5)/SiO_2. (a) BF-TEM. (b) STEM-HAADF. Scale bar: 20 nm........ 25

Figure S28. TEM-EDX of Zn(5)/SiO_2. Scale bar: 20 nm........ 25

Figure S29. Cu K-edge XANES of Cu foil, Cu/SiO_2, and Cu-Zn(5)/SiO_2........ 26

Figure S30. Fitting of the k^3-weighted EXAFS data of the different catalysts and references: (a) Cu foil. (b) Cu/SiO_2. (c) Cu-Zn(5)/SiO_2. 26

Figure S31. Zn K-edge XANES spectra of CuMes-Et_2Zn(5)/SiO_2 and Cu-Zn(5)/SiO_2........ 27

Figure S32. Zn K-edge XAS of Cu-Zn(5)/SiO_2 and references: (a) XANES. (b) Fourier-transform of the k^3-weighted EXAFS........ 27

Figure S33. Zn K-edge XAS of Cu-ZnO-Al_2O_3. (a) XANES. (b) The first derivative of the XANES. (c) Fourier-transform of the k^3-weighted EXAFS........ 28

Figure S34. Temperature-programmed reduction in 5% H_2/Ar after oxidation of the specimen in 5% O_2/He at room temperature. 28

Figure S35. CO_2 conversion for Cu-Zn(2)/SiO_2, Cu-Zn(5)/SiO_2, and Cu-Zn(10)/SiO_2 with respect to contact time (230 °C, 25 bar, H_2/CO_2/N_2 = 3:1:1). 29

Figure S36. Formation rates of CH_3OH and CO for Cu-Zn(2)/SiO_2, Cu-Zn(5)/SiO_2, Cu-Zn(10)/SiO_2, or Cu-Zn(20)/SiO_2 with respect to contact time, extrapolated to zero conversion (zero contact time) with second-order polynomial fits. 30

Figure S37. Intrinsic formation rates normalized per mass of Cu for Cu-Zn(5)/SiO_2 and Cu-ZnO-Al_2O_3 obtained by extrapolation to zero conversion (zero contact time) together with the respectively selectivities for CH_3OH, specified above the respective bars (230 °C, 25 bar, $H_2/CO_2/N_2$ = 3:1:1).......31

Figure S38. Intrinsic formation rates normalized per mass of surface Cu sites (denoted Cu^0(surf)) with the respective selectivities to CH_3OH (specified above the respective bars) for the catalysts tested (230 °C, 25 bar, $H_2/CO_2/N_2$ = 3:1:1). 31

Figure S39. CH_3OH and CO selectivity at ca. 100 hours of TOS for Cu-Zn(5)/SiO_2 (230 °C, 25 bar, $H_2/CO_2/N_2$ = 3/1/1, contact time 0.06 s g mL^{-1})..32

Figure S40. A representative TEM and particle size distribution of Cu-Zn(5)/$SiO_{2\text{-TOS100h}}$. (a) HAADF-STEM (scale bar: 20 nm). (b) Particle size distribution. 32

Figure S41. TEM-EDX of Cu-Zn(5)/$SiO_{2\text{-TOS100h}}$. Scale bar: 2 nm. 33

Figure S42. Cu and Zn K-edge XAS of fresh Cu-Zn(5)/SiO_2 and after 100 h of TOS. (a) Cu K-edge XANES. (b) Zn K-edge XANES. (c) Fourier-transform of the k^2-weighted Zn K-edge EXAFS. 33

Figure S43. Peak deconvolution of the Zn $2p_{3/2}$ XPS spectra of the freshly reduced Cu-Zn(5)/SiO_2 and Zn(5)/SiO_2. Pretreatment conditions: 200 °C, 1 h, 300 mbar H_2. 34

Figure S44. Zn $2p_{3/2}$ XPS spectra of Cu-Zn(5)/SiO_2 and Zn(5)/SiO_2 after exposure of the activated materials to 120 mbar of methanol vapor (230 °C, 1 h). 34

Figure S45. Cu LMM Auger spectra of Cu/SiO_2. (a) Passivated under 1% O_2/N_2 for 2 h. (b) Reduced under 300 mbar H_2 at 100 °C for 1 h. (c) Reduced under 300 mbar H_2 at 200 °C for 1 h. 35

Figure S46. Cu LMM Auger spectra of Cu-Zn(5)/SiO_2. (a) Passivated under 1% O_2/N_2 for 2 h. (b) Reduced under 300 mbar H_2 at 100 °C for 1 h. (c) Reduced under 300 mbar H_2 at 200 °C for 1 h. 35

Figure S47. Cu 2p XPS spectra of Cu/SiO_2 and Cu-Zn(5)/SiO_2 before and after exposure to the reaction mixture of H_2 (300 mbar) and CO_2 (100 mbar) at 230 °C for 1 h. Cu 2p XPS region is not sensitive enough to separate contributions from Cu^0 and Cu^{+1}. 36

Figure S48. Cu LMM Auger spectra of Cu/SiO_2 and Cu-Zn(5)/SiO_2 pre-treated at 300 mbar of H_2 at 200 °C prior to and after their exposure to the reaction mixture of H_2 and CO_2 (300 and 100 mbar, respectively) at 230 °C for 1 h. 36

Figure S49. Cu LMM Auger spectra of activated Cu/SiO_2 and Cu-Zn(5)/SiO_2 after their exposure to methanol vapor (300 mbar, 230 °C, 1 h). 37

Figure S50. Cu(Zn)/Si atomic ratio of the Cu-Zn(5)/SiO_2 material from XPS. 37

Figure S51. Schematic representation of the temperature profile during the *in situ* XAS experiment of passivated Cu-Zn(5)/SiO_2. (a) TPR (conditions: r.t. → 300 °C, H_2, 10 sccm, 1 bar, ramp: 5 °C min^{-1}), and (b) CO_2 hydrogenation reaction (conditions: 230 °C, H_2/Ar = 3:2 → H_2/CO_2/Ar = 3:1:1, 10 sccm, 11 bar). 38

Figure S52. Schematic representation of a single gas switch experiment as shown in Figure S51b (conditions: 230 °C, H_2/Ar = 3:2 → H_2/CO_2/Ar = 3:1:1, 10 sccm, 11 bar)..38

Figure S53. (a) Cu K edge XANES during TPR. (b) MCR-ALS modelled XANES spectra at the Cu K edge during the TPR process (conditions: r.t. → 300 °C, H_2, 10 sccm, 1 bar, ramp: 5 °C min^{-1}). 39

Figure S54. k^2-weighted Cu K edge EXAFS for Cu foil. (a) R-space (Window (blue) 1–3 Å, Hanning window, dk = 0.5). (b) K-space (Window (blue) 3.0–14.0 $Å^{-1}$, Hanning window, dk = 1). Fitting results are summarized in Table S7. 40

Figure S55. k^2-weighted Cu K edge EXAFS for passivated Cu-Zn(5)/SiO_2. (a) R-space (Window (blue) 1–2.7 Å, Hanning window, dk = 1). (b) K-space (Window (blue) 3.0–11.5 $Å^{-1}$, Hanning window, dk = 0.5). Fitting results are summarized in Table S7. 41

Figure S56. k^2-weighted Cu K edge EXAFS for Cu-Zn(5)/SiO_2 after hydrogen treatment and before the gas switching experiment. (a) R-space (Window (blue) 1–3 Å, Hanning window, dk = 1). (b) K-space (Window (blue) 3.0–12.0 $Å^{-1}$, Hanning window, dk = 0.5). Fitting results are summarized in Table S7. 42

Figure S57. (a) Zn K edge XANES during TPR. (b) MCR modelled Zn K edge XANES spectra during TPR. Conditions: r.t. → 300 °C, H_2, 10 sccm, 1 bar, ramp: 5 °C min^{-1}. 43

Figure S58. Cu K edge XANES before and after gas switching (i.e. introduction of CO_2). Conditions: 230 °C, 3:2 H_2/Ar → 3:1:1 H_2/CO_2/Ar, 10 sccm, 11 bar. 43

Figure S59. k^2-weighted Cu K edge EXAFS for Cu-Zn(5)/SiO_2 after the *in situ* gas switching experiment. (a) R-space (Window (blue) 1–3 Å, Hanning window, dk = 1). (b) K-space (Window (blue) 3.0–12.0 $Å^{-1}$, Hanning window, dk = 0.5). 44

Figure S60. (a) Zn K edge XANES during gas switch (introduction of CO_2). (b) MCR modelled Zn K edge XANES spectra after the gas switch (introduction of CO_2). Conditions: 230 °C, 3:2 H_2/Ar → 3:1:1 H_2/CO_2/Ar, 10 sccm, 11 bar). The point of zero sec is defined as the time when CO_2 was introduced into the H_2 containing gas feed. 45

Figure S61. MCR-ALS analysis of the Zn K edge XANES during a multiple-cycle gas switching experiments between H_2/Ar (3:2) and H_2/CO_2/Ar (3:1:1) at 230 °C and 11 bar. Cycle length: 1200 seconds (600 sec + 600 sec), 20 mg Cu-Zn(5)/SiO_2 catalyst, 10 sccm. 45

Figure S62. Solid-state MAS NMR of Cu-Zn(5)/SiO_2 after exposure to H_2 and $^{13}CO_2$ (H_2/$^{13}CO_2$ = 3/1, total pressure 5 bar, 230 °C, 12 h). (a) ^{1}H spectrum (B_0 = 9.4 T, 298 K, 4 mm probe, $\nu_L(^1H)$ = 400 MHz, NS = 64). (b) ^{13}C CP-MAS spectrum (B_0 = 9.4 T, 298 K, 4 mm probe, $\nu_L(^1H)$ = 400 MHz, $\nu_L(^{13}C)$ = 100.6 MHz, NS = 3688). 46

Figure S63. ^{1}H–^{13}C heteronuclear correlation (HETCOR) spectrum of (a) Cu/SiO_2 and (b) Cu-Zn(5)/SiO_2 after exposure to H_2 and $^{13}CO_2$ (H_2/$^{13}CO_2$ = 3:1, total pressure 5 bar, 230 °C, 12 h). 2D HETCOR NMR spectra were recorded with 128 scans in the direct and 64 increments in the indirect dimensions. For homonuclear decoupling, e-DUMBO scheme was used. 46

Figure S64. Solid-state MAS NMR of Cu/SiO_2 after exposure to H_2 and $^{13}CO_2$ (H_2/$^{13}CO_2$ = 3/1, total pressure 5 bar, 230 °C, 12 h). (a) ^{1}H spectrum (B_0 = 9.4 T, 298 K, 4 mm probe, $\nu_L(^1H)$ = 400 MHz, NS = 64). (b) ^{13}C CP-MAS spectrum (B_0 = 9.4 T, 298 K, 4 mm probe, $\nu_L(^1H)$ = 400 MHz, $\nu_L(^{13}C)$ = 100.6 MHz, NS = 12272). 47

Figure S65. Experimental procedures for transient *operando* DRIFTS for modulated He/CO_2 and H_2/He feeds. Reduction condition: 500 °C under 20 ml min^{-1} of H_2 for 2 h. Reaction conditions: ca. 30 mg catalyst, 230 °C, 20 bar, He/CO_2 and H_2/He = 3:1, total flow rate 20 ml min^{-1}....................................47

Figure S66. Single beam spectra (background) collected under He prior to the introduction of the CO_2 + H_2 feed: (a) without dilution and (b) with dilution with SiO_2. Reduction condition: 500 °C under 20 ml min^{-1} of H_2 for 2 h. Reaction conditions: ca. 30 mg catalyst, 230 °C, 20 bar, H_2/CO_2 = 3/1, total flow rate 20 ml min^{-1}..48

Figure S67. Time-resolved *operando* DRIFT spectra of surface species formed during CO_2 hydrogenation over Cu/SiO_2 diluted with SiO_2 (1:3). Reduction condition: 500 °C under 20 ml min^{-1} of H_2 for 2 h. Reaction conditions: ca. 30 mg catalyst, 230 °C, 20 bar, H_2/CO_2 = 3:1, total flow rate 20 ml min^{-1}. 48

Figure S68. Time-resolved *operando* DRIFT spectra of surface species formed during CO_2 hydrogenation over (a–b) Cu/SiO_2 and (c–d) Cu-Zn/SiO_2 catalysts. Reduction condition: 500 °C under 20 ml min^{-1} of H_2 for 2 h. Reaction conditions: ca. 30 mg catalyst, 230 °C, 20 bar, H_2/CO_2 = 3:1, total flow rate 20 ml min^{-1}..49

Figure S69. O*perando* DRIFTS during the switching between He+CO_2 and H_2+He feeds over (a) Cu/SiO_2 and (b) CuZn(5)/SiO_2. Reduction condition: 500 °C under 20 ml min^{-1} of H_2 for 2 h. Reaction conditions: ca. 30 mg catalyst, 230 °C, 20 bar, He/CO_2 and H_2/He = 3:1, total flow rate 20 ml min^{-1}. 50

Supplementary Tables *51*

Table S1. Cu and Zn loading of Cu-Zn/SiO_2 materials. 51

Table S2. Cu and Zn loading and Cu particle size of the materials prepared...51

Table S3. Cu-Cu shell parameters obtained by Cu K-edge EXAFS fitting of the different catalysts.....51

Table S4. Quantity of surface Cu^0 sites determined through N_2O titration..52

Table S5. Total H_2 consumption calculated from bulk TPR (Cu was pre-oxidized to CuO by O_2)........52

Table S6. Comparison of the catalysts in this study with benchmark catalysts in the literature............53

Table S7. Summary of Cu K-edge EXAFS fitting results obtained from the in situ XAS experiment. .53

Table S8. Assignment of surface species in DRIFTS experiments. 54

References *55*

Experimental part

Catalyst preparation. SiO_2 (Aerosil 300) was compacted, sieved (180–300 μm), calcined in static air (500 °C, 12 h, 1 °C min^{-1}), evacuated to ca. 10^{-5} mbar (500 °C, 20 h) and then transferred, while hot, to a nitrogen-filled glovebox. This support is referred to as SiO_{2-500}. Silica-grafted copper mesityl, CuMes/SiO_{2-500}, was prepared via a surface organometallic chemistry approach,[1] using Cu_x(Mesityl)$_x$, where x = 2, 4, 5, denoted CuMes (Strem Chemicals), as described previously.[2,3] Briefly, in a glovebox, SiO_{2-500} (1 g, 0.59 mmol of ≡SiOH) was dispersed in 5 mL of dry toluene (dried by molecular sieve packed column, water content < 5 ppm) and contacted with a solution of CuMes (72 mg, 0.39 mmol of Cu) in 5 mL of toluene. The reaction mixture was stirred at 100 r.p.m for 3 h at room temperature. After this time, the solid was washed with toluene (3 × 5 mL) and dried (ca. 10^{-5} mbar) at room temperature for 3 h to give the material denoted CuMes/SiO_{2-500}. This material (300 mg) was then exposed to pulses of diethylzinc (Pegasus Chemicals, pulse duration was 0.1 s; 2, 5, 10 or 20 pulses were used) at 150 °C in the ALD chamber (Picosun R-200). Materials obtained are referred to as CuMes-Et_2Zn(n)/SiO_2, where n is the number of pulses used. Before the catalytic tests, the materials were loaded, inside a glovebox, to a plug flow reactor and pretreated under undiluted H_2 (50 mL min^{-1}) at 500 °C for 2 h. The reference methanol synthesis catalyst (Alfa Aesar) contained 63.5 wt% CuO, 25 wt% ZnO, 10 wt% Al_2O_3, and 1.5 wt% MgO. This material was reduced under H_2 at 250 °C for 3.5 h, i.e., pretreated according to published protocols.[4,5]

Catalyst characterization. The surface area and pore volume of the materials were determined by N_2 physisorption (Quantachrome NOVA 4000e) with the Brunauer-Emmet-Teller (BET) model[6] (using the adsorption data) and Barrett-Joyner-Halenda (BJH) model[7] (using the desorption data), respectively. Before the measurements, the samples were outgassed at 250 °C for 2.5 h. The Cu and Zn loadings of the catalysts were determined, after digestion in *aqua regia*, by inductively coupled plasma optical emission spectroscopy (ICP-OES) using an Agilent 5100 VDV instrument. Fourier-Transform Infrared (FTIR) spectroscopy experiments were performed on self-supporting wafers using a Bruker Alpha spectrometer in transmission mode (24 scans, 4 cm^{-1} resolution) under a N_2 atmosphere. Intensities were normalized to the Si–O–Si overtones of the silica support. Powder X-ray diffraction (XRD) data were collected on a PANalytical Empyrean X-ray diffractometer equipped with a Bragg-Brentano HD mirror operated at 45 kV and 40 mA using Cu Kα radiation (λ = 1.5418 Å). The materials were examined within the 2θ range of 5–90° using a step size of 0.0167°. The scan time per step was 3 s. Transmission electron microscopy (TEM), scanning transmission electron microscopy (STEM) with a high-angle annular dark-field (HAADF) detection, and energy-dispersive X-ray (EDX) spectroscopy were carried out on an FEI Talos F200X transmission electron microscope. Gold grids were used. If not noted otherwise, all samples were passivated under 1% O_2/N_2 at room temperature for 2 h before the transfer to the TEM instrument.

X-ray photoelectron spectra (XPS) were recorded on a SPECS (Germany) photoelectron spectrometer using a hemispherical PHOIBOS-150-MCD-9 analyzer (Mg K_α radiation, $h\nu$ = 1253.6 eV, 150 W). The binding energy (BE) scale was pre-calibrated using the positions of the peaks of Au $4f_{7/2}$ (BE = 84.0 eV) and Cu $2p_{3/2}$ (BE = 932.67 eV) core levels. The Si 2p peak at 103.5 eV of the SiO_2 support was used as an internal standard. The survey and narrow spectra were recorded at a pass energy of the analyzer of 50 and 20 eV, respectively. To determine the chemical (charge) state of elements on the surface of the samples, the regions Si 2p, C 1s, Cu LMM, O 1s, Cu 2p, and Zn $2p_{3/2}$ were measured. The atomic ratios of the elements on the catalyst surface were calculated from the integral photoelectron peak intensities which were corrected using theoretical sensitivity factors based on Scofield's photoionization cross sections.[8] The residual gas pressure

during the measurements did not exceed 8 × 10^{-9} mbar. To carry out experiments with the high-pressure cell of the SPECS photoelectron spectrometer, all samples were rubbed into a stainless steel mesh spot welded onto a standard holder.

Ex situ X-ray absorption spectra (XAS) at the Cu and Zn K-edges were measured at the SuperXAS beamline (X10DA) at the Swiss Light Source (SLS, PSI, Villigen, Switzerland), operating in top-up mode at a 2.4-GeV electron energy and a current of 400 mA. Cu and Zn XAS spectra were collected at the K-edge using a Si (111) monochromator in transmission mode with continuous scanning between 8760 and 10792 eV with a step size of 0.1 eV. Calibration of the monochromator energy position was performed by setting the inflection point of a Cu or Zn foil spectrum recorded simultaneously with the sample to 8979 eV or 9659 eV for Cu or Zn K-edges, respectively. The *ex situ* samples were sealed in a capillary in the glovebox and analyzed without exposure to air.

In situ Cu and Zn XAS spectra of Cu-Zn(5)/SiO_2 were acquired simultaneously at the SuperXAS beamline at SLS. The incident photon beam was selected by a liquid nitrogen cooled Si (111) quick-EXAFS monochromator and the rejection of higher harmonics and focusing were achieved by a rhodium-coated collimating mirror. The beam size on the sample was approximately 2000 × 500 μm. During measurement, the quick XAS monochromator was rotating with a frequency of 1 Hz in a 3° angular range and X-ray absorption spectra were collected in transmission mode using ionization chambers specifically developed for rapid data collection with a frequency of 1 MHz. The *in situ* EXAFS spectra used for fitting were collected after cooling (<50 °C), for EXAFS 600 scans were acquired (10 minutes acquisition) and averaged. For Cu and Zn, a Zn reference foil was used for energy calibration (9659.0 eV). The edge energy is set at the maximum of the first derivative of the normalized XANES. For the *in situ* experiment, approximately 20 mg of the passivated powder sample (180–300 μm) was packed into a 3 mm quartz capillary (i.d. 2.8 mm, bed length ca. 1 cm) which was integrated into a pressurizable gas flow system consisting of 2 parallel arrays, each consisting of 3 mass flow controllers (Bronkhorst), while the total pressure was maintained by a back-pressure regulator (Bronkhorst EL-Press). Switching between the two systems (i.e. switching the MFC array that was feeding the capillary) was performed using a remotely controlled 6-port 2-way switching valve (VICI, Valco) that could be operated from outside the experimental hutch. While one gas mixture was flowing to the cell, the other was directed via a bypass to the exhaust. Samples were heated using a custom-built infrared heater (Elstein-Werk M. Steinmetz GmbH & Co. KG (Germany), 30 mm length, with two heating elements – one above and one below the sample capillary), and the temperature was controlled using a 0.3-mm K-type thermocouple placed in direct contact with the catalyst bed. Ar and H_2 were purified by passing through a trap containing molecular sieves and Q5 catalyst prior to introduction to the XAS quartz cell. CO_2 was purified by passing through a trap containing activated molecular sieves.

In the *in situ* experiment, Ar (10 sccm, 1 bar) was flowed over the passivated catalyst for 10 minutes while the spectra were recorded for 10 minutes. The gas flow was then changed to H_2 (10 sccm, 1 bar), and hydrogen treatment was performed under the same flow using a temperature ramp of 5 °C min^{-1}, reaching a final temperature of 300 °C. Spectra were recorded continuously during the hydrogen treatment. The sample was then cooled, (<50 °C) under a flow of H_2 to enable collection of data to be used for EXAFS analysis of the as-reduced material (600 scans, 10 minutes), before being heated to 230 °C under H_2. The gas composition was again changed to H_2/Ar (3:2, 10 sccm), and subsequently pressurized to 11 bar. Once the pressure had stabilized, data acquisition was started, and the sample was measured continuously for 30 minutes. After 10 minutes of acquisition, the gas composition was switched to H_2/Ar/CO_2 (3:1:1, 10 sccm)

using the 6-port valve, to capture any changes upon the introduction of CO_2 to the reaction gas. The delay between switching of the gas compositions and CO_2 reaching the catalyst bed was estimated to be approximately 40 seconds, based on the reduced absorption of the beam (drop in baseline of spectrum prior to normalization) upon replacing a fraction of the Ar in the feed with less-absorbing CO_2, using a strategy similar to a previously reported protocol.[9] After reaction, the sample was depressurized and cooled under a flow of Ar (10 sccm) and data was collected for EXAFS fitting (600 scans, 10 minutes). A schematic representation of the experimental protocol is given in Figure S51.

The processing of the XAS data was performed with the ProQEXAFS and the Athena software,[10,11] and EXAFS fittings were conducted with the Artemis software.[11] The S_0^2 value (0.876) for the Cu K edge was obtained by fitting a Cu foil (See Figure S54). Coordination numbers were fixed for this fit. MCR analysis of the XANES data was performed using the in-built feature of the ProQEXAFS software. For this purpose, an energy range of 8900–9100 eV was used for Cu K-edge data, and 9600–9800 eV was used for Zn K-edge data.

H_2 TPD and TPR experiments were performed using an AutoChem system (Micromeritics) with a thermal conductivity detector (TCD). In a typical H_2 TPD experiment, ca. 50 mg of a passivated material was loaded in air and reduced *in situ* at 500 °C under a H_2 flow (50 mL min^{-1}) for 2 h. Subsequently, the specimen was purged with Ar for 30 min (50 mL min^{-1}) at 500 °C and cooled down to −50 °C under Ar. The sample was then saturated in 5% H_2/Ar flow (50 mL min^{-1}) for 30 min and purged with Ar for another 30 min. Finally, the sample was heated to 500 °C at 10 °C min^{-1} under an Ar flow and the desorbed H_2 was monitored with the TCD detector. The curves of H_2 TPD were fitted using the Origin Software and Gaussian peak shapes. For the N_2O titration measurements, the passivated materials were first pretreated under H_2 at 500 °C, followed by purging and cooling down to 50 °C under Ar. The gas was then switched to 5% N_2O/Ar for 60 min to oxidize the surface Cu^0 sites to Cu^{+1} and then reduced with 5% H_2/Ar from room temperature to 500 °C (10 °C min^{-1}). Similarly, the TPR experiments were carried out under 5% H_2/Ar from room temperature to 500 °C after the sample was oxidized by 5% O_2/He at room temperature.

Solid-state NMR spectra were recorded on a Bruker 400 MHz spectrometer using a double resonance 4 mm CP-MAS probe. Samples were packed in 4 mm zirconia rotors inside an Ar-filled glovebox, and recorded at 298 K. In all cases, the downfield ^{13}C resonance of adamantane (38.4 ppm) was used as an external secondary reference to calibrate the chemical shifts. The MAS frequency was set to 10 kHz. For the ^{1}H-^{13}C HETCOR experiment, DUMBO homonuclear (^{1}H-^{1}H) decoupling was used during t_1. To prepare ^{13}C-labelled surface intermediates, the activated Cu-Zn(5)/SiO_2 catalyst was loaded into a thick-walled glass reactor inside an argon-filled glovebox. The reactor was evacuated, and $^{13}CO_2$ was introduced (1 bar), and then condensed under liquid nitrogen cooling (–196 °C). Then, 1 bar of H_2 was introduced while still maintaining cooling with liquid nitrogen at –196 °C. The reactor was then heated to 230 °C (10 °C min^{-1}) and kept for 12 h. After 12 h, the reaction vessel was cooled to –196 ºC, evacuated at 10^{-5} mbar, and allowed to return to room temperature over the course of 20 minutes whilst dynamic vacuum was maintained. The resulting solid was transferred to an argon-filled glovebox and packed to 4 mm zirconia rotors for solid-state NMR analysis. To prepare ^{13}C-labelled surface intermediates on Cu/SiO_2, it was treated an identical way as Cu-Zn(5)/SiO_2, as described above.

CO_2 hydrogenation tests. CO_2 hydrogenation was performed in a tubular fixed-bed reactor (304.8 mm total length, 9.1 mm internal diameter, Hastelloy X, Microactivity Effi, PID Eng&Tech).[12] In a typical experiment, the catalyst (100 mg) was first pretreated under H_2 at 500 °C for 2 h. The catalytic test was

performed at 230 °C under 25 bar. The gas flow of $H_2/CO_2/N_2$ (3/1/1), N_2 as internal standard, was passed through the catalyst bed and the products were analyzed online by a GC (PerkinElmer Clarus 580) equipped with thermal conductivity and flame ionization detectors, and a methanizer. Different contact times (space velocities) were probed by changing the gas flow rate from 100 to 15 NmL min^{-1}. The formation rate, CO_2 conversion, and methanol selectivity were calculated using the following equations:

$$F_{x,out}[molh^{-1}] = C_{x,out}\frac{F_{N_2,\in}}{C_{N_2,out}} \quad (1)$$

$$r_x[gh^{-1}g_{Cu}^{-1}] = \frac{F_{x,out}}{m_{Cu}} \times MW_x \quad (2)$$

$$X_{CO_2} = \frac{\Sigma_{i=1}^{n}F_{x,out}}{F_{CO_2,\in}} \quad (3)$$

$$S_{MeOH} = \frac{F_{MeOH,out}}{\Sigma_{i=1}^{n}F_{x,out}} \quad (4)$$

where $F_{x,out}$ is the outlet flow rate of methanol or CO [mol h^{-1}]; $C_{x,out}$ is the outlet gas fraction of species x; $F_{x,in}$ is the inlet flow rate of species x [mol h^{-1}]; r_x is the formation rate of methanol or CO [g h^{-1} $g_{Cu}{}^{-1}$]; m_{Cu} is the mass of Cu used in the reaction [g]; MW_x is the molecular weight of methanol or CO [g mol^{-1}]; X_{CO2} is the conversion of CO_2; S_{MeOH} is the selectivity to methanol. Intrinsic formation rates and selectivities were extrapolated using a second-order polynomial fit of the experimental data. At least three experimental data points were averaged for each presented data point.

***Operando* DRIFTS.** The catalyst powder (50–100 mg) was placed in a cylindrical cavity of a Harrick Praying Mantis High Temperature Reaction Chamber and the cell was mounted in a diffuse reflection (DRIFTS) accessory. The spectra were collected using a Thermo Scientific Nicolet 6700 FT-IR spectrometer equipped with a liquid-nitrogen-cooled MCT detector at a resolution of 4 cm^{-1}. The flow of gases was set using mass flow controllers (Bronkhorst). The switching between two reactant gas streams was performed using a 4-way valve. The pressure of the two gas streams (to the cell and to the vent) was controlled by backpressure regulators (Bronkhorst). The outlet gas stream was analyzed by a Pfeiffer OmniStar GSD 300C mass spectrometer. Prior to the measurements, the specimen was reduced *in situ* at 500 °C in a H_2 stream (20 Nml min^{-1}) for 2 h and subsequently cooled in H_2 to the reaction temperature (230 °C). The cell was pressurized to 20 bar and immediately exposed to the reactant mixture (H_2/CO_2 = 3/1, total flow 20 Nml min^{-1}) at 20 bar via a switching valve. The spectra were acquired continuously every 20 seconds (50 scans) in a time-resolved manner, to monitor the appearance and the evolution of surface species. Transient DRIFTS utilizes a periodic perturbation of a system by external parameters (stimulation) to influence the concentration of the active species.[13] The transient DRIFTS experiment was performed in the above-mentioned setup by using a switching valve to change the stream of the reactant gases allowing for a periodic perturbation of the gas concentration, as shown in Figure S65. No baseline correction was applied to the time-resolved spectra. Multivariate spectral analysis was performed using the multivariate curve resolution-alternating least squares (MCR-ALS) algorithm, as described elsewhere.[14] MCR is a chemometric method used for data processing and deconvolution of complex spectra down to individual components based on kinetic resolution. MCR can provide the response profiles (e.g. spectra, time profiles, etc.) of the individual chemical species of an unresolved mixture when no previous information is available about the nature and composition of these mixtures.

Supplementary Figures

Optimization of the catalyst preparation method and H_2 pretreatment temperature. This section provides results and a discussion of additional catalysts to those presented in the main text. These additional catalysts differ in their preparation method. In particular, if a H_2 pretreatment temperature is lowered from 500 °C to 300 °C, it gives a Cu-Zn(5)/$SiO_{2-300-H2}$ catalyst with inferior methanol formation rate and methanol selectivity relative to Cu-Zn(5)/$SiO_{2-500-H2}$ presented in the main text (ca. 2.1 g h^{-1} g_{Cu}^{-1} and 63%, respectively, Figure S1). Considering the similar Cu particle size (1.8 ± 0.4 nm, Figure S2) in these two materials and an even slightly higher amount of surface Cu^0 sites in Cu-Zn(5)/$SiO_{2-300-H2}$ (169 μmol g_{cat}^{-1},Table S4), this result indicates that the formation of the more active structure of Cu and Zn requires high pre-treatment temperatures (ca. 500 °C) that exceed the reduction temperatures determined in the TPR experiments (Figure S3).

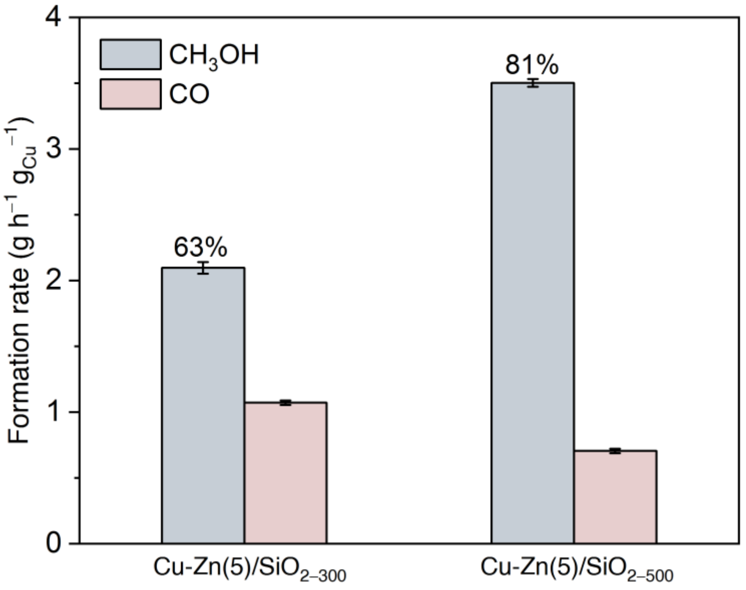

Figure S1. Formation rates normalized per mass of Cu of Cu-Zn(5)/SiO_2 catalysts after different H_2 treatment temperatures together with their selectivities for CH_3OH, specified above the respective bars (230 °C, 25 bar, $H_2/CO_2/N_2$ = 3:1:1, contact time 0.06 s g mL^{-1}).

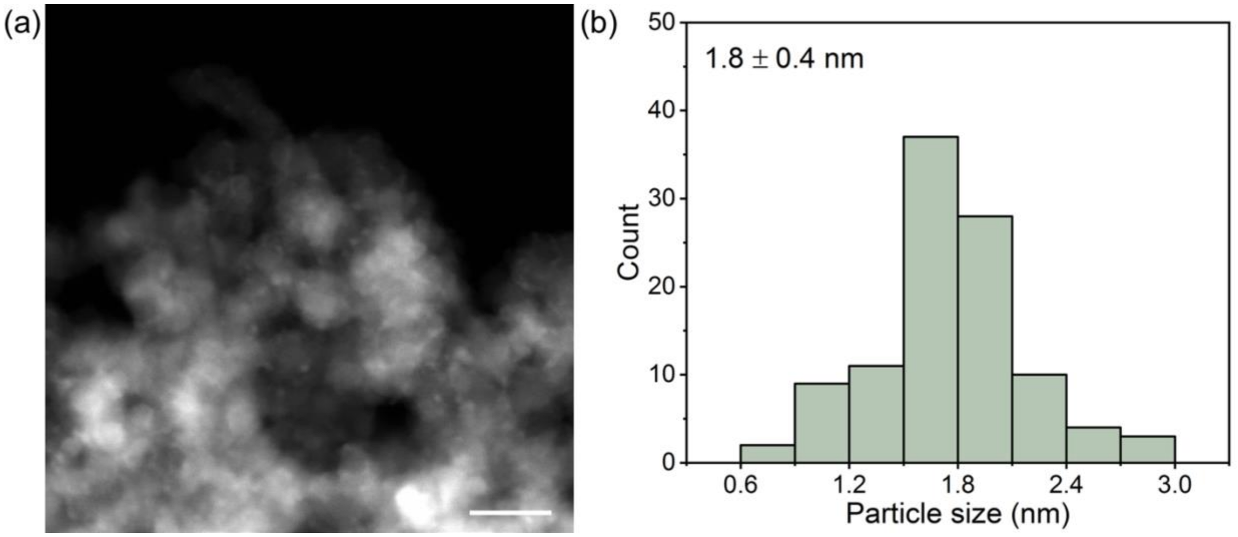

Figure S2. A representative TEM image and particle size distribution of Cu-Zn(5)/$SiO_{2\text{–}300}$. (a) HAADF-STEM (scale bar: 20 nm). (b) Particle size distribution.

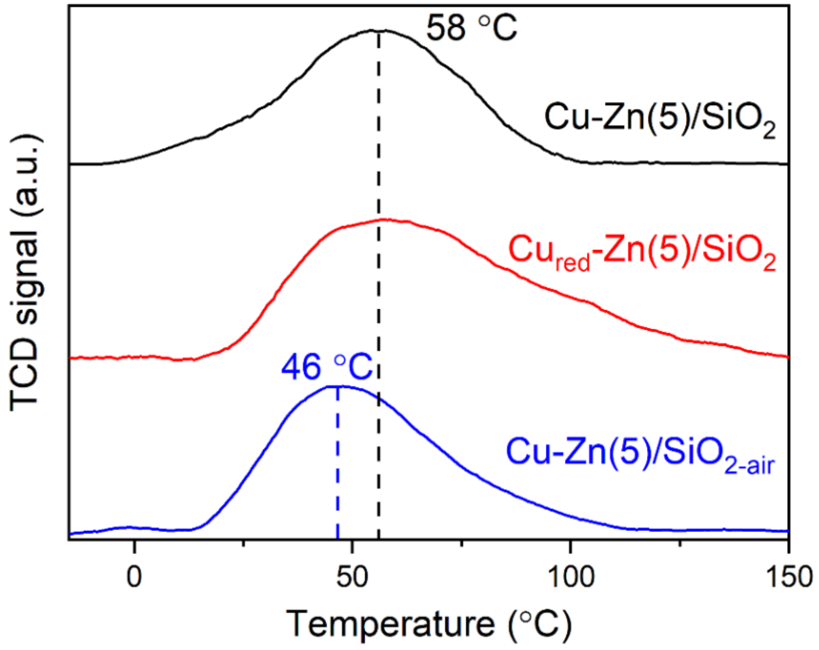

Figure S3. H_2 temperature-programmed desorption of the catalysts after saturation in 5% H_2/Ar.

Order of Cu and Zn introduction. We have also synthesized a catalyst by reversing the order of introducing Zn and Cu, that is, first pulses of Et_2Zn were performed onto SiO_{2-500}, followed by the grafting of Cu via SOMC. This material is denoted as Zn(5)-Cu/SiO_2 and after H_2 treatment (500 °C, 2 h) its methanol formation rate is 2.3 g h^{-1} g_{Cu}^{-1}, which is notably lower than that of Cu-Zn(5)/SiO_2 (Figures S4 and S5). A similar Zn enrichment around the perimeter of Cu NPs, as in Cu(5)-Zn/SiO_2, is observed in Zn(5)-Cu/SiO_2 from the TEM-EDX maps, however, the formed Cu NPs have a size of 3.4 ± 1.0 nm (Figures S6 and S7), which is larger than those in Cu-Zn(5)/SiO_2.

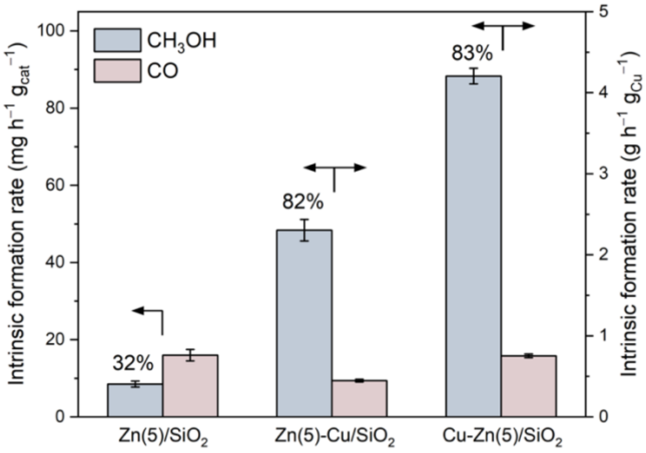

Figure S4. Intrinsic formation rates of Zn(5)/SiO_2, Zn(5)-Cu/SiO_2, and Cu-Zn(5)/SiO_2 obtained by extrapolation to zero conversion (zero contact time) together with the respective selectivities for CH_3OH, specified above the respective bars (230 °C, 25 bar, $H_2/CO_2/N_2$ = 3:1:1).

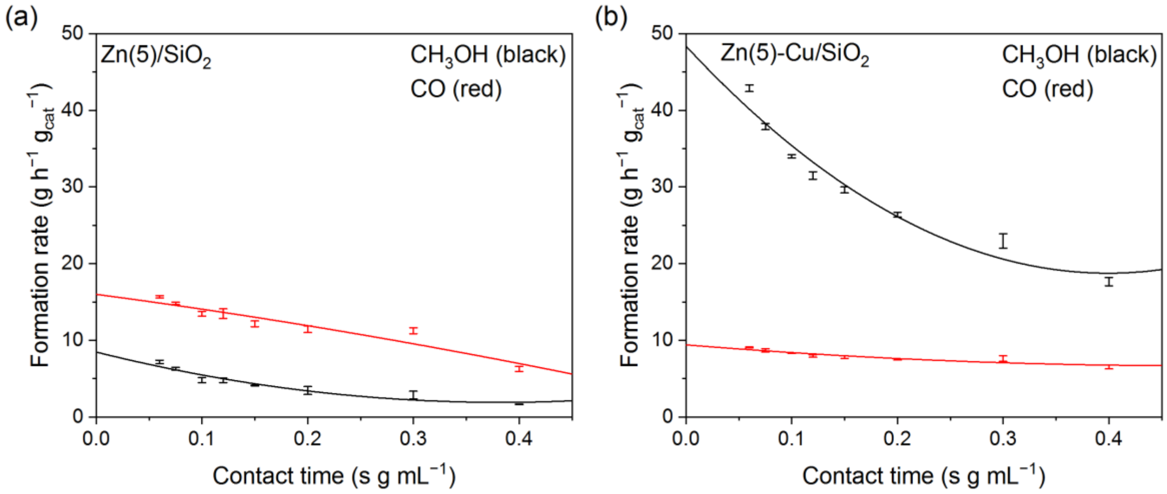

Figure S5. Formation rates of CH_3OH and CO for Zn(5)/SiO_2 and Zn(5)-Cu/SiO_2 with respect to the contact time extrapolated to zero conversion (zero contact time) using second-order polynomial fits.

Note that the formation rates of CH_3OH and CO for Cu/SiO_2 with respect to contact time have been reported.[3]

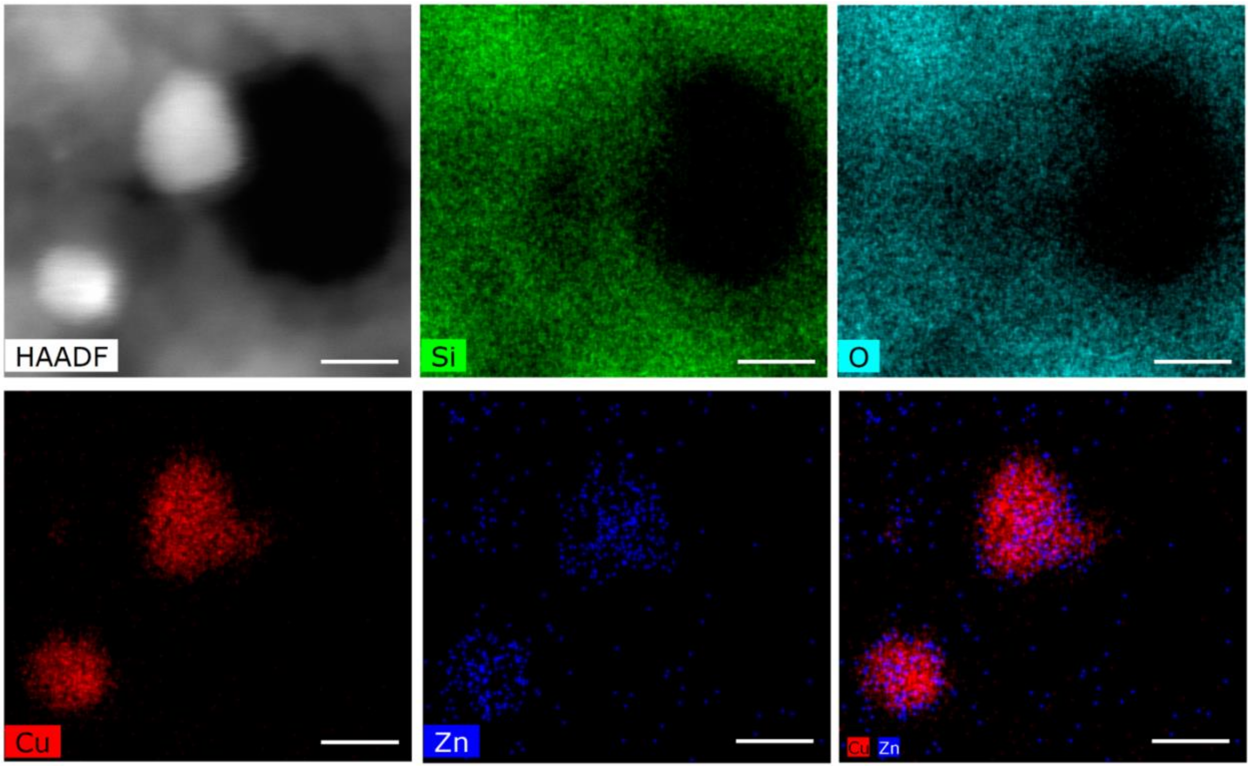

Figure S6. TEM-EDX of Zn(5)-Cu/SiO_2. Scale bar: 6 nm.

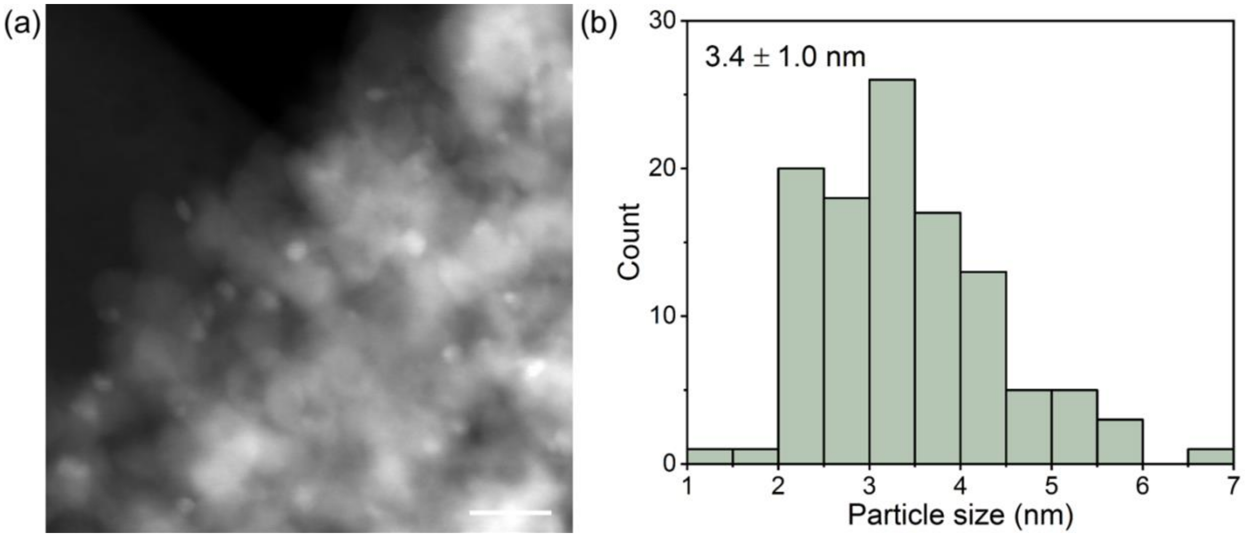

Figure S7. TEM and particle size of Zn(5)-Cu/SiO_2. (a) HAADF-STEM (scale bar: 20 nm). (b) Particle size distribution.

Reduction of CuMes before Zn ALD. To investigate if the interaction between Cu and Zn can be engineered from a preformed Cu/SiO_2, we reduced $CuMes/SiO_2$ under H_2 at 500 °C and subsequently deposition Zn using 5 pulses of Et_2Zn. After a H_2 treatment (500 °C, 2 h), the material denoted Cu_{red}-Zn(5)/SiO_2 was obtained (see Figures S8, S9, and S10 for characterization). Interestingly, Cu_{red}-Zn(5)/SiO_2 shows a low intrinsic methanol formation rate of 0.9 g h^{-1} g_{Cu}^{-1} (Figures S11 and S12). TEM reveals that the Cu particle size in Cu_{red}-Zn(5)/SiO_2 is 3.1 ± 0.6 nm and the mappings show that Zn is not enriched around the Cu NPs. These results indicate that the enrichment of Zn around Cu NPs only occurred when molecular silica-grafted CuMes sites interact with Et_2Zn species.

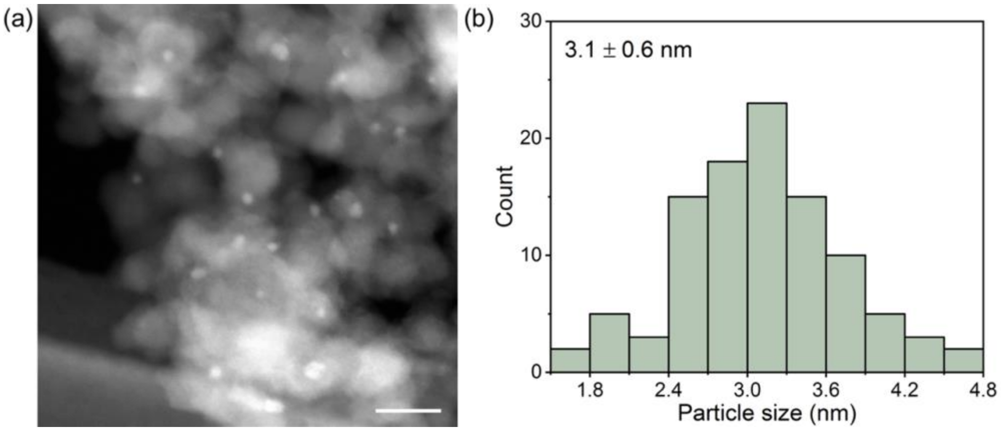

Figure S8. TEM and particle size distribution of Cu_{red}-Zn(5)/SiO_2. (a) HAADF-STEM (scale bar: 20 nm). (b) Particle size distribution.

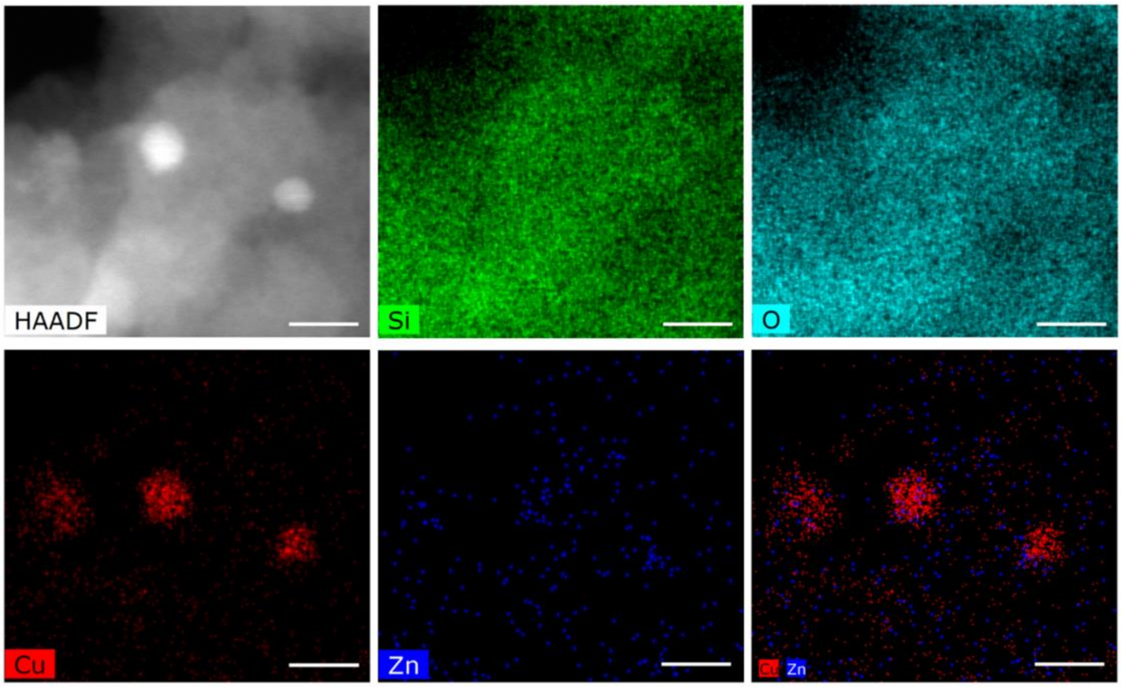

Figure S9. TEM-EDX maps of Cu_{red}-Zn(5)/SiO_2. Scale bar: 6 nm.

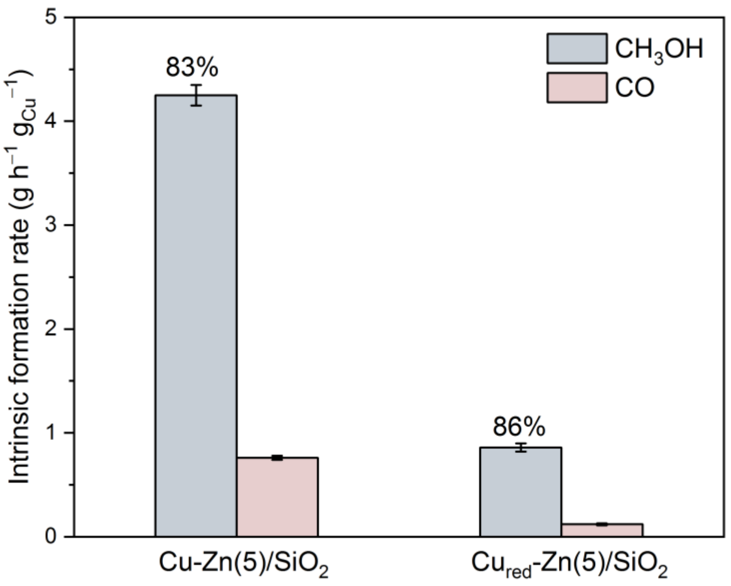

Figure S10. Intrinsic formation rates normalized per mass of Cu of Cu-Zn(5)/SiO_2 and Cu_{red}-Zn(5)/SiO_2 obtained by extrapolation to zero conversion (zero contact time, Figure S11) together with their respective selectivities for CH_3OH, specified above the respective bars (230 °C, 25 bar, $H_2/CO_2/N_2$ = 3/1/1).

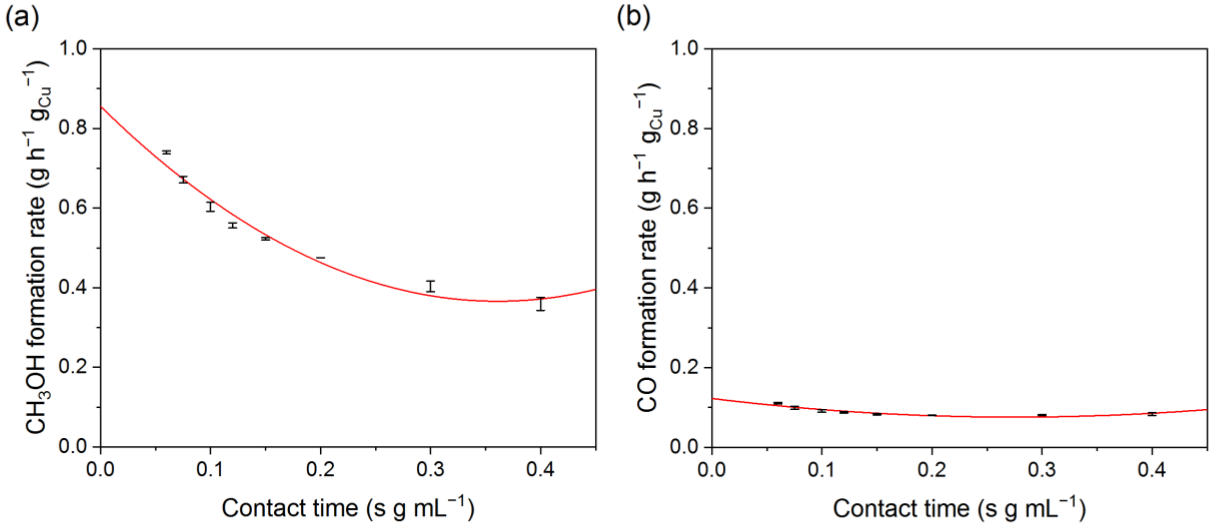

Figure S11. Formation rates of CH_3OH and CO for Cu_{red}-Zn(5)/SiO_2 with respect to the contact time; extrapolated to zero conversion (zero contact time) using second-order polynomial fits.

Air exposure. Exposure of CuMes-$Et_2Zn(5)/SiO_2$ to air leads to a rapid change in its color from dark brown to green, indicating the formation of CuO in the air-exposed material (Figure S12). Interestingly, Cu-Zn(5)/SiO_{2-air} shows, after the typical H_2 pretreatment at 500 °C, a low activity in CO_2 hydrogenation to methanol (0.6 g h^{-1} g_{Cu}^{-1} methanol formation rate, Figure S13). TEM-EDX maps show that the Cu particle size in Cu-Zn(5)/$SiO_{2-air-H2}$ grew to 2.7 ± 0.4 nm (Figure S14), and Zn is selectively enriched around the Cu NPs (Figure S15). The hydrogen desorption peak, after saturation of Cu-Zn(5)/$SiO_{2-air-H2}$ in 5% H_2/Ar, shifts to a lower temperature of 46 °C, i.e., by ca. 12 °C as compared to Cu-Zn(5)/SiO_2 (Figure S3). This data indicates that the active and selective Cu–Zn sites characteristic for Cu-Zn(5)/SiO_2 do not form after the exposure of CuMes-$Et_2Zn(5)/SiO_2$ to air. However, if CuMes-$Et_2Zn(5)/SiO_2$ is first converted to Cu-Zn(5)/SiO_2 under H_2 (500 °C, 2 h), and then exposed to air at room temperature, the activity of the catalyst (pretreated in H_2 before the test) is only slightly lower than that of the catalyst that has not been exposed to air (Figure S13). Lastly, we passivated Cu-Zn(5)/SiO_2 under 1% O_2/N_2 for 2 h and then exposed the material to air. In contrast to the exposure to air of CuMes-$Et_2Zn(5)/SiO_2$, the color of the passivated Cu-Zn(5)/SiO_2 did not change to green and remained black (Figure S12). The activity and selectivity of the passivated catalyst in CO_2 hydrogenation can be fully recovered after H_2 pretreatment (500 °C, 2 h, Figures S13 and S16). To conclude, passivation is an effective measure to handle Cu-Zn(5)/SiO_2 under air without compromising its catalytic performance.

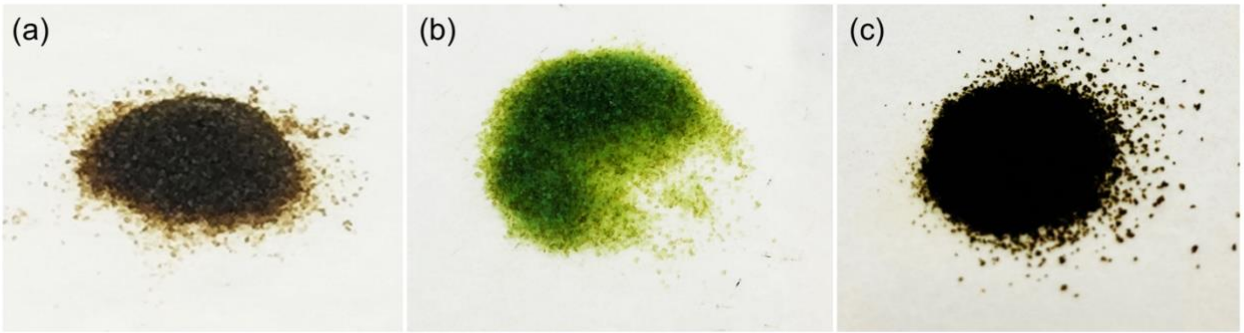

Figure S12. Images of Cu-Zn(5)/SiO_2 catalysts after different pretreatments: (a) CuMes-$Et_2Zn(5)/SiO_2$ without air exposure. (b) CuMes-$Et_2Zn(5)/SiO_2$ after air exposure. (c) CuMes-$Et_2Zn(5)/SiO_2$ after reduction (H_2, 500 °C, 2 h), passivation (1% O_2/N_2, room temperature, 2 h), and air exposure.

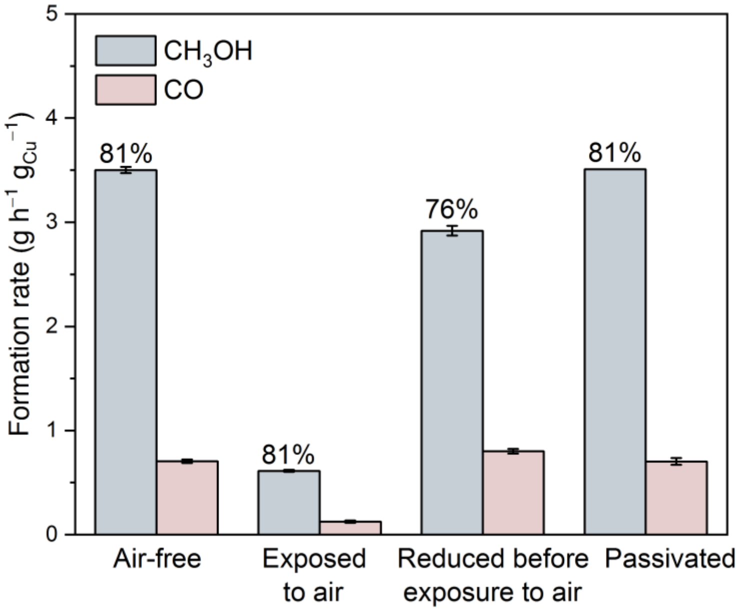

Figure S13. Formation rates normalized per mass of Cu of Cu-Zn(5)/SiO_2 after different pretreatment procedures together with the selectivities for CH_3OH, specified above the respective bars (230 °C, 25 bar, $H_2/CO_2/N_2$ = 3:1:1, contact time 0.06 s g mL^{-1}). Air-free: the catalyst was loaded inside a glovebox with exposure to air. Exposed to air: the catalyst was loaded in air. Reduced before exposure to air: the catalyst was reduced (H_2, 500 °C, 2 h) and then loaded in air. Passivated: the catalyst was reduced (H_2, 500 °C, 2 h), passivated (1% O_2/N_2, room temperature, 2 h), and loaded in air. All the catalysts were in-situ reduced under H_2 (500 °C, 2 h) before CO_2 hydrogenation.

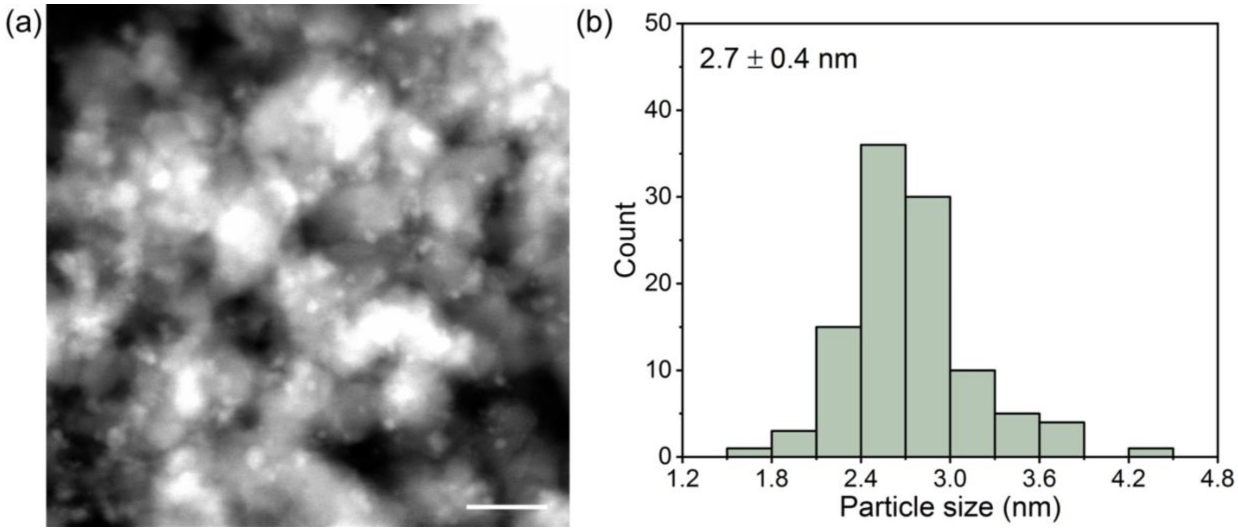

Figure S14. A representative TEM image and particle size distribution of Cu-Zn(5)/$SiO_{2\text{-air}}$. (a) HAADF-STEM (scale bar: 20 nm). (b) Particle size distribution.

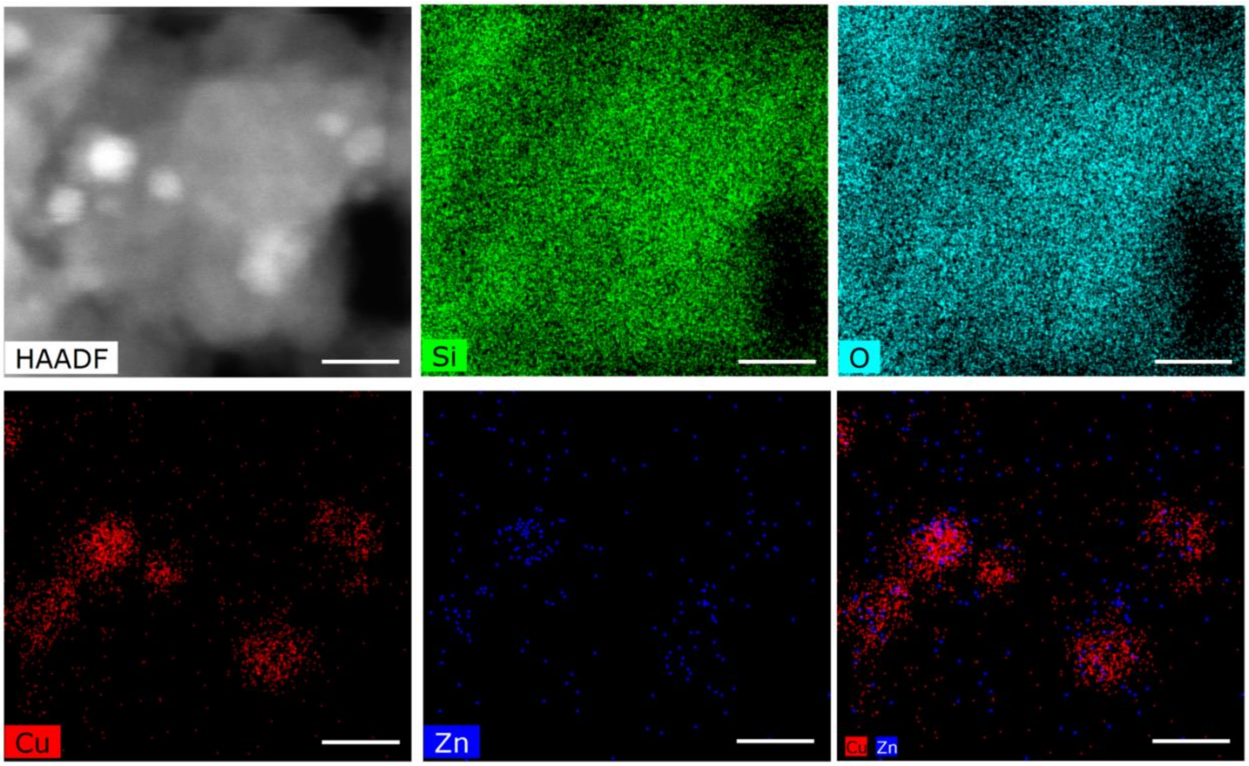

Figure S15. TEM-EDX of Cu-Zn(5)/$SiO_{2\text{-air}}$. Scale bar: 6 nm.

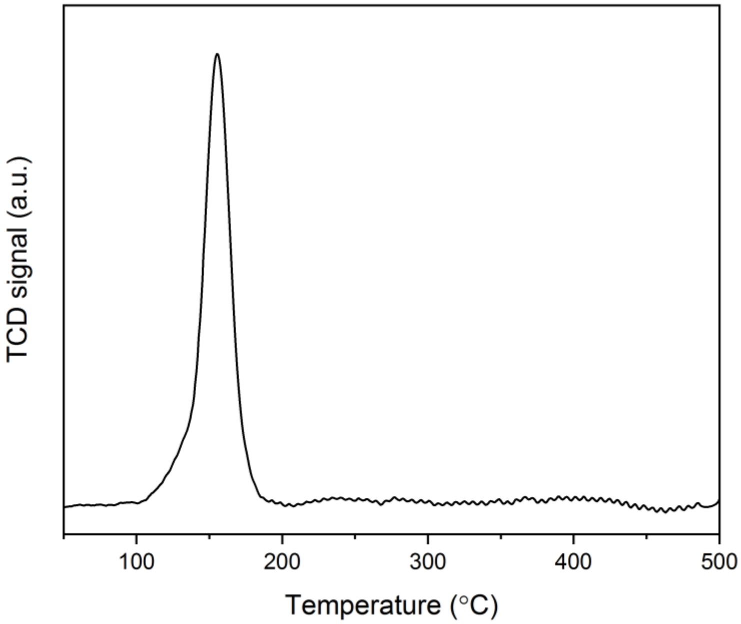

Figure S16. Temperature-programmed reduction of passivated Cu-Zn(5)/SiO_2 (1% O_2/N_2, 2 h) using 5% H_2/Ar.

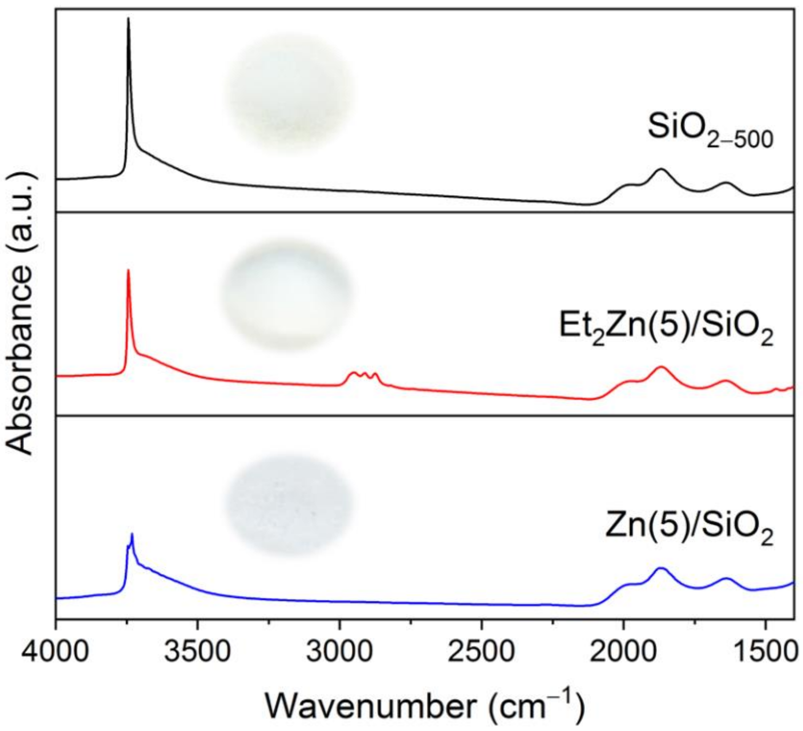

Figure S17. Transmission IR spectra of SiO_{2-500}, $Et_2Zn(5)/SiO_2$, and $Zn(5)/SiO_2$.

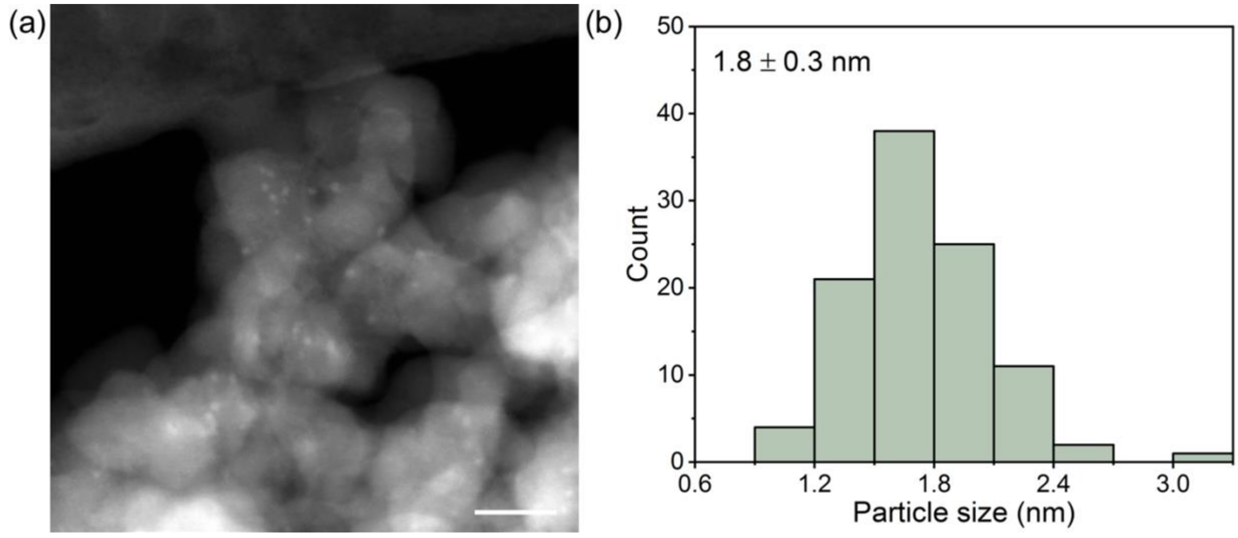

Figure S18. A representative TEM image and particle size distribution of CuMes-$Et_2Zn(5)/SiO_2$. (a) HAADF-STEM (scale bar: 20 nm). (b) Particle size distribution.

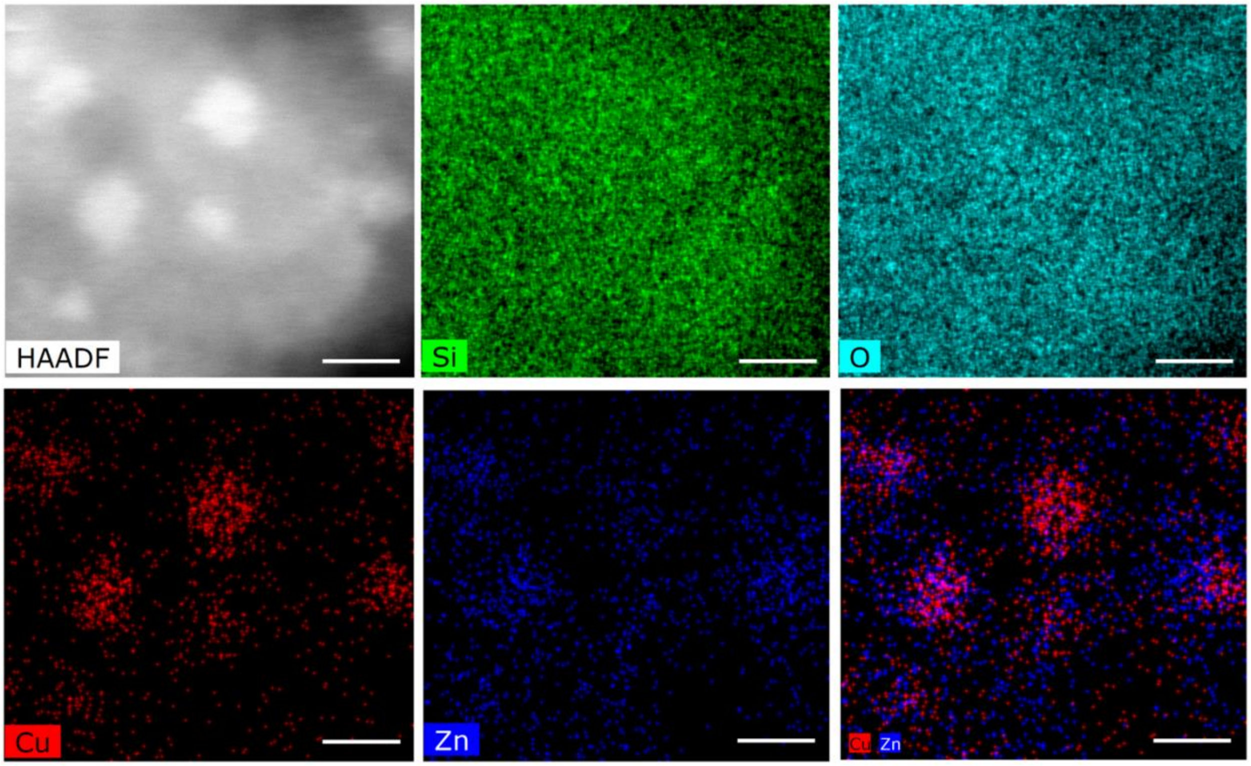

Figure S19. TEM-EDX maps of CuMes-$Et_2Zn(5)/SiO_2$. Scale bar: 3 nm.

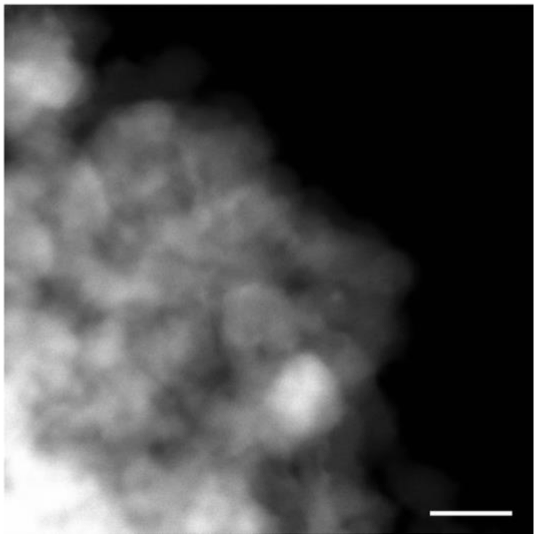

Figure S20. HAADF-STEM image of CuMes/SiO_2. Scale bar: 20 nm.

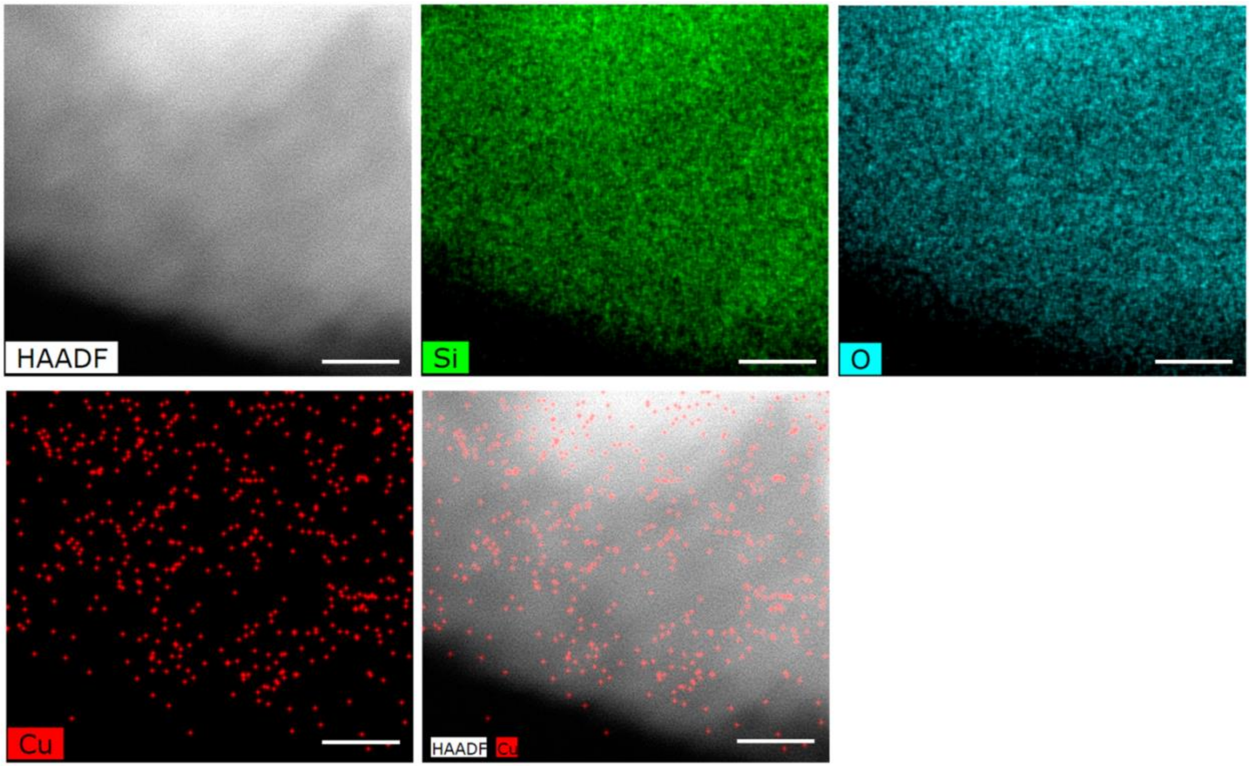

Figure S21. TEM-EDX maps of $CuMes/SiO_2$. Scale bar: 6 nm.

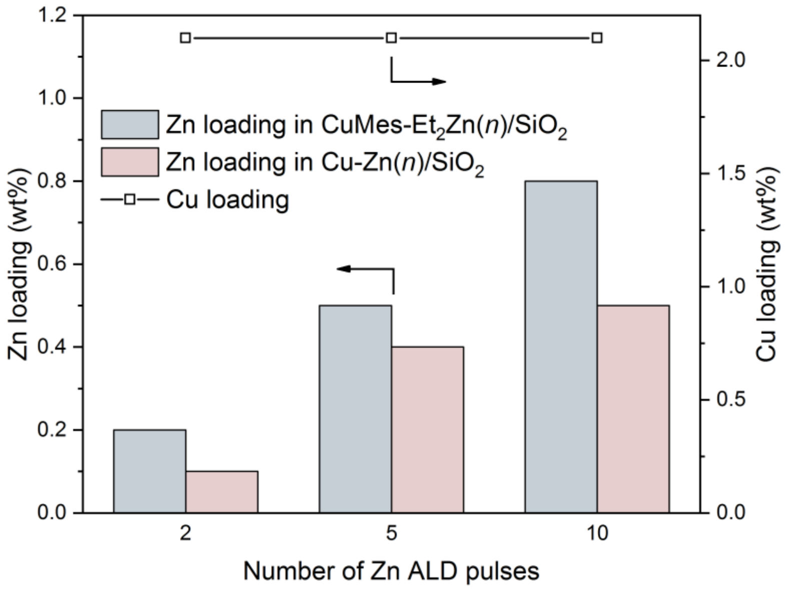

Figure S22. Cu and Zn loading in $CuMes\text{-}Et_2Zn(n)/SiO_2$ and $Cu\text{-}Zn(n)/SiO_2$ (n = 2, 5, 10).

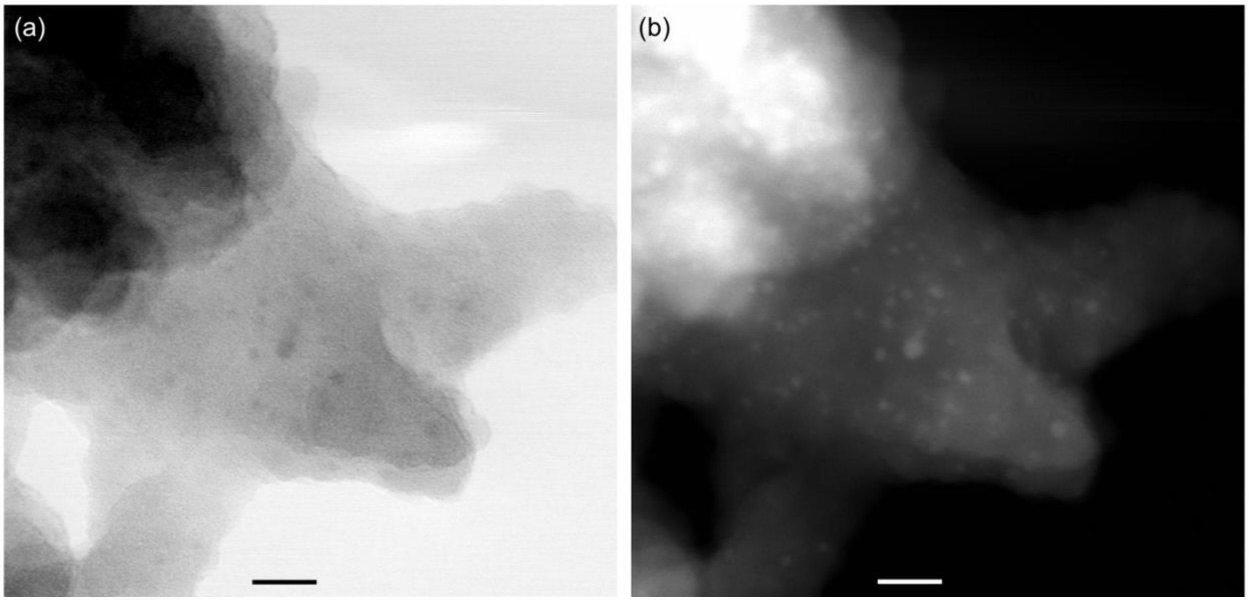

Figure S23. TEM of Cu-Zn(5)/SiO_2. (a) BF-TEM. (b) STEM-HAADF. Scale bar: 10 nm.

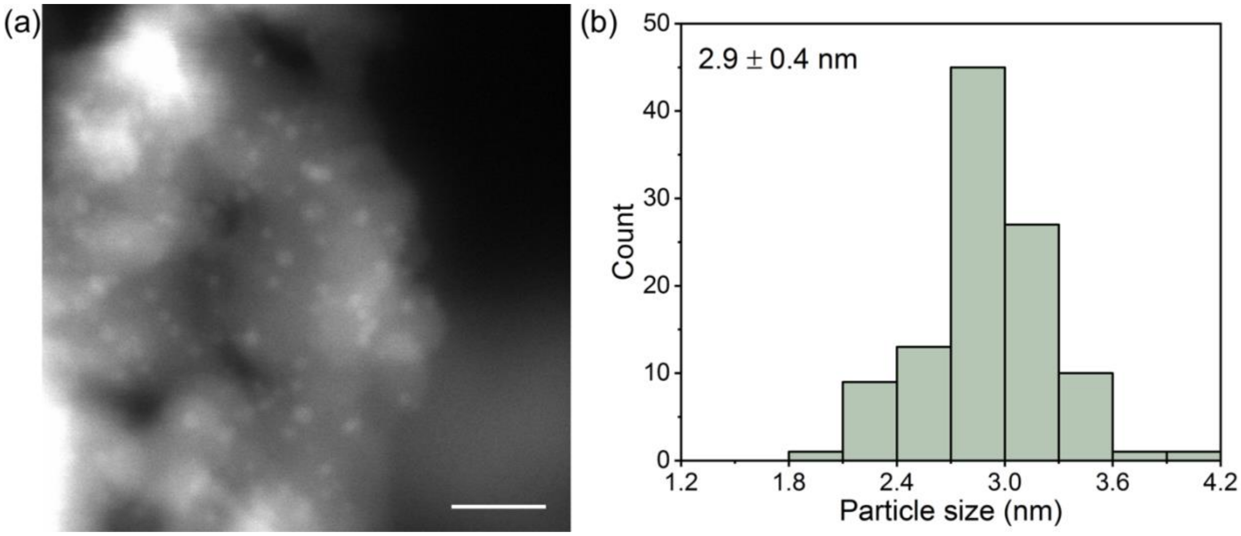

Figure S24. A representative TEM image and particle size of Cu/SiO_2. (a) HAADF-STEM (scale bar: 20 nm). (b) Particle size distribution.

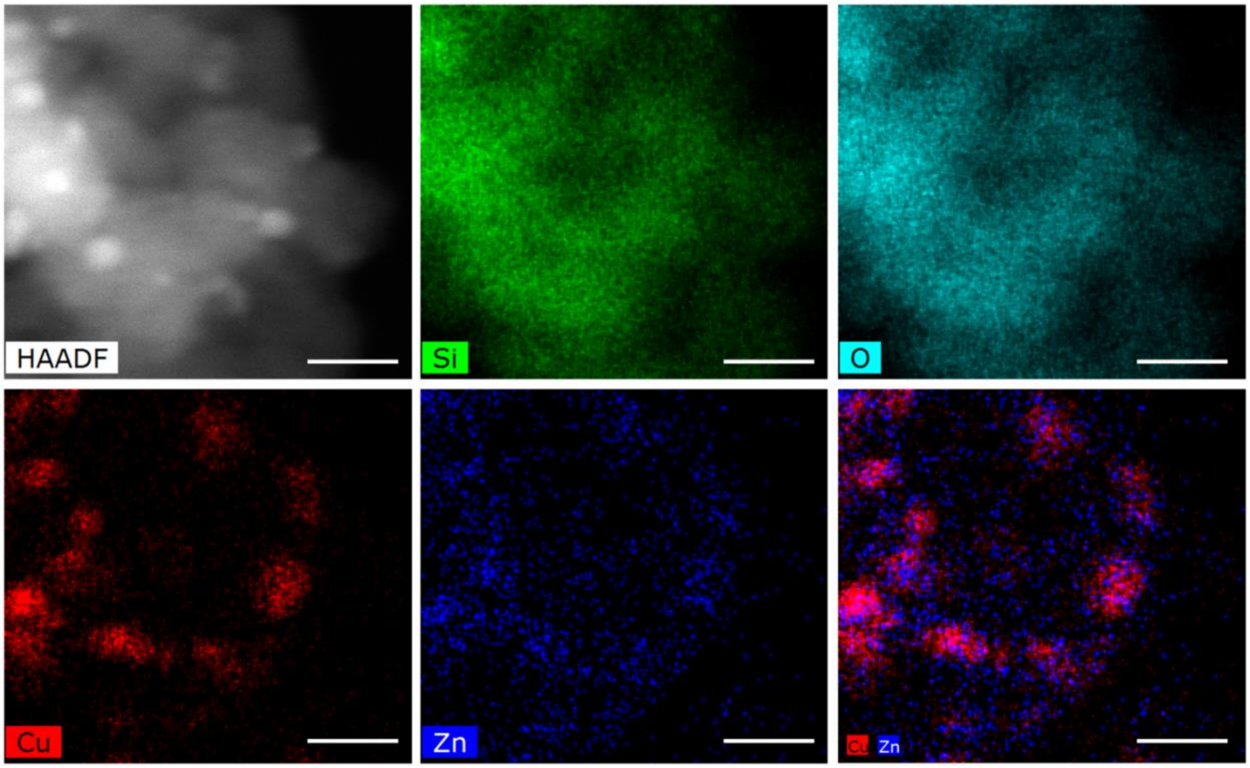

Figure S25. TEM-EDX maps of Cu-Zn(5)/SiO_2. Scale bar: 10 nm.

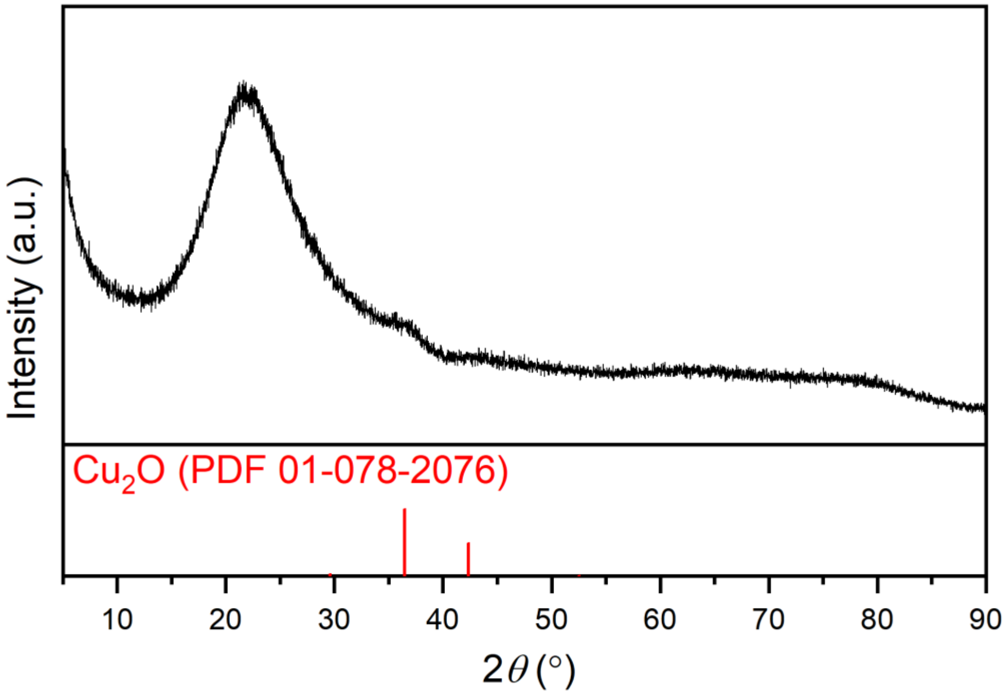

Figure S26. XRD pattern of Cu-Zn(5)/SiO_2.

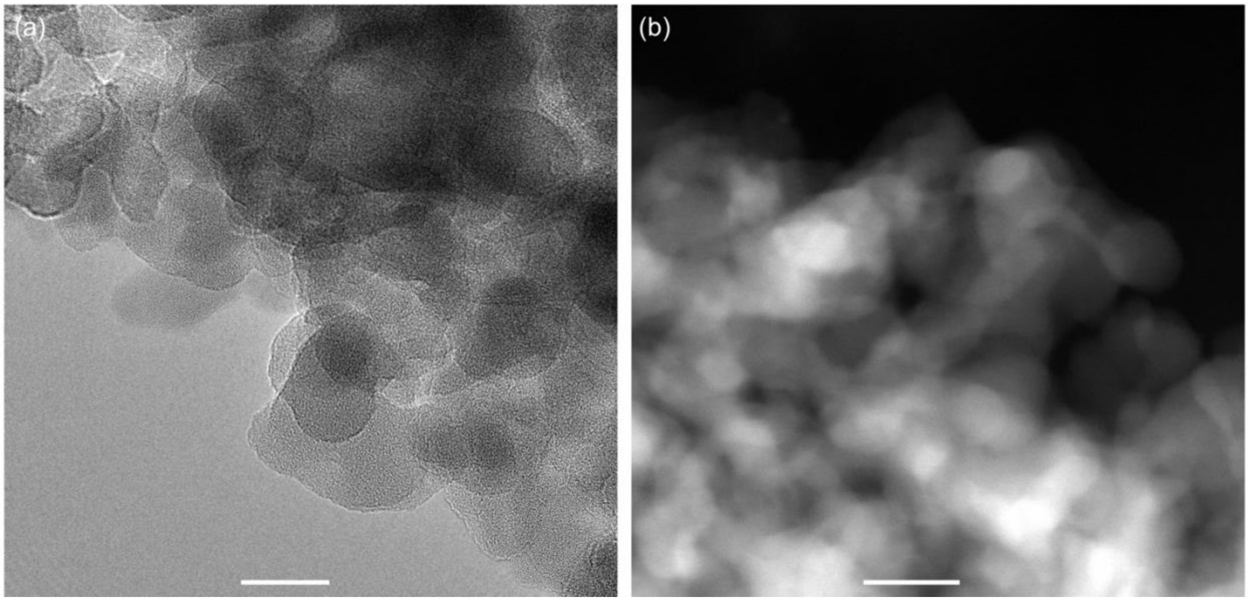

Figure S27. TEM of Zn(5)/SiO_2. (a) BF-TEM. (b) STEM-HAADF. Scale bar: 20 nm.

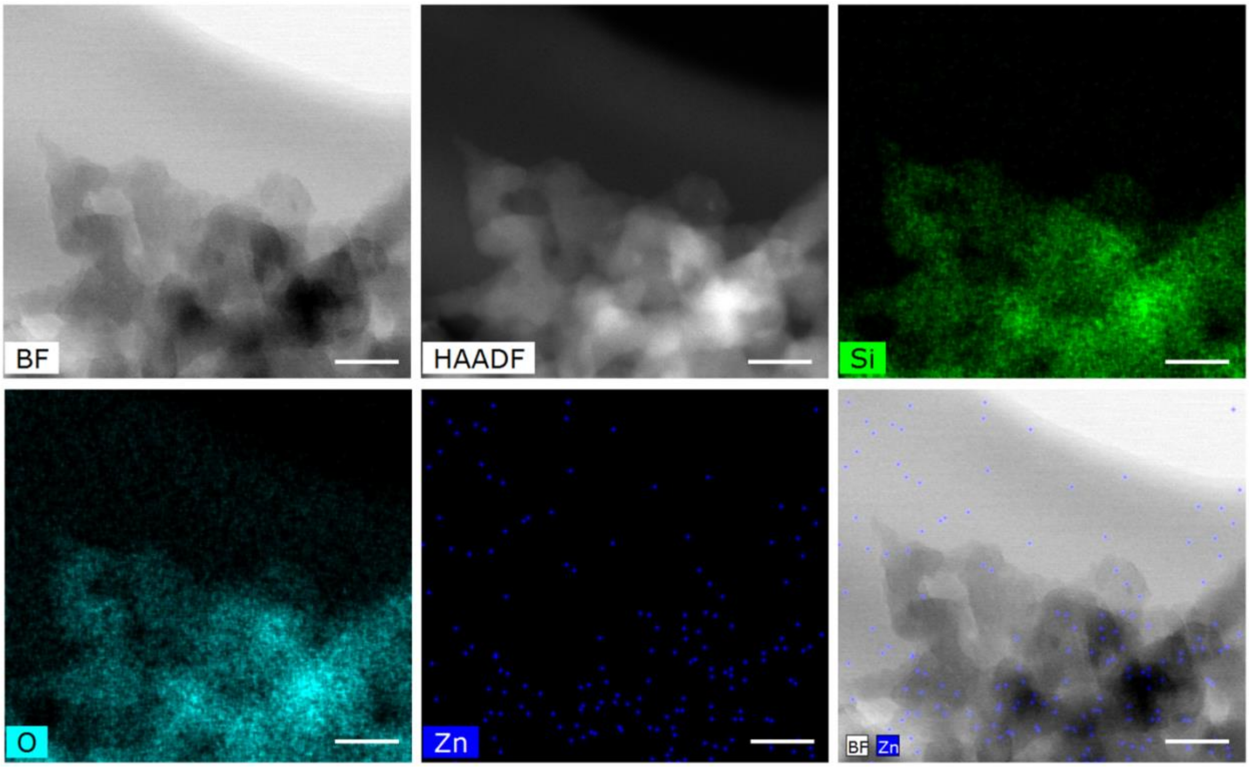

Figure S28. TEM-EDX of Zn(5)/SiO_2. Scale bar: 20 nm.

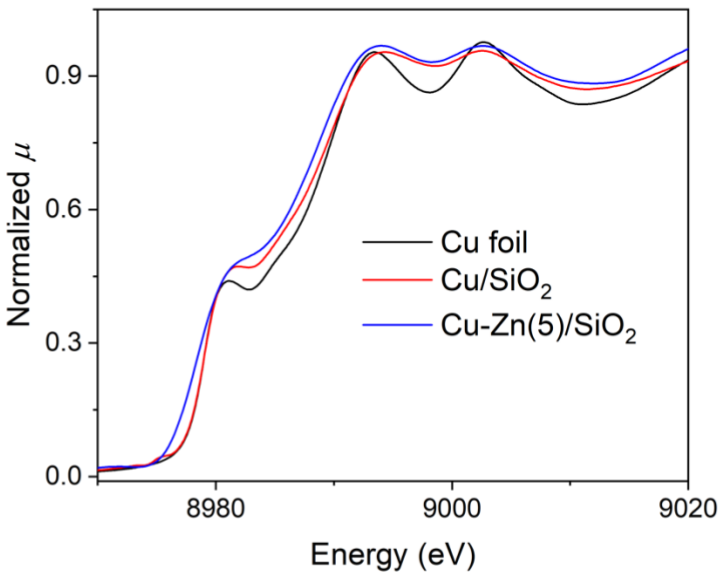

Figure S29. Cu K-edge XANES of Cu foil, Cu/SiO_2, and Cu-Zn(5)/SiO_2.

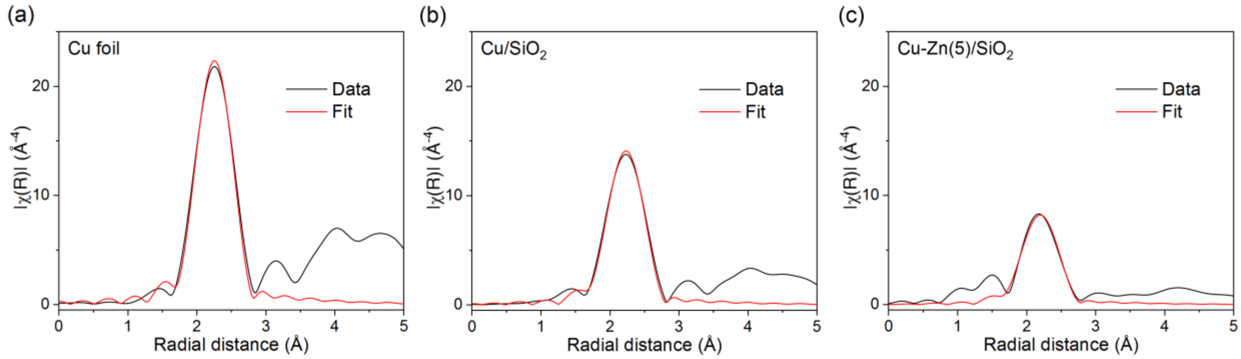

Figure S30. Fitting of the k^3-weighted EXAFS data of the different catalysts and references: (a) Cu foil. (b) Cu/SiO_2. (c) Cu-Zn(5)/SiO_2.

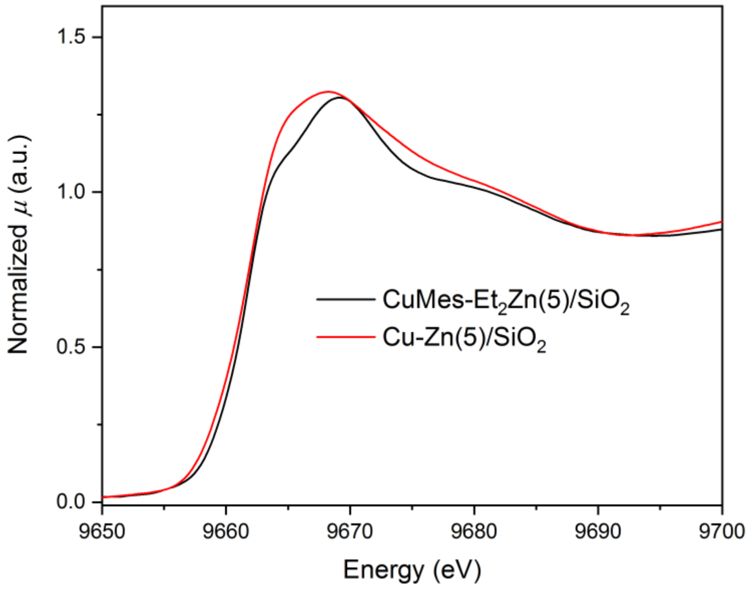

Figure S31. Zn K-edge XANES spectra of CuMes-$Et_2Zn(5)/SiO_2$ and Cu-$Zn(5)/SiO_2$.

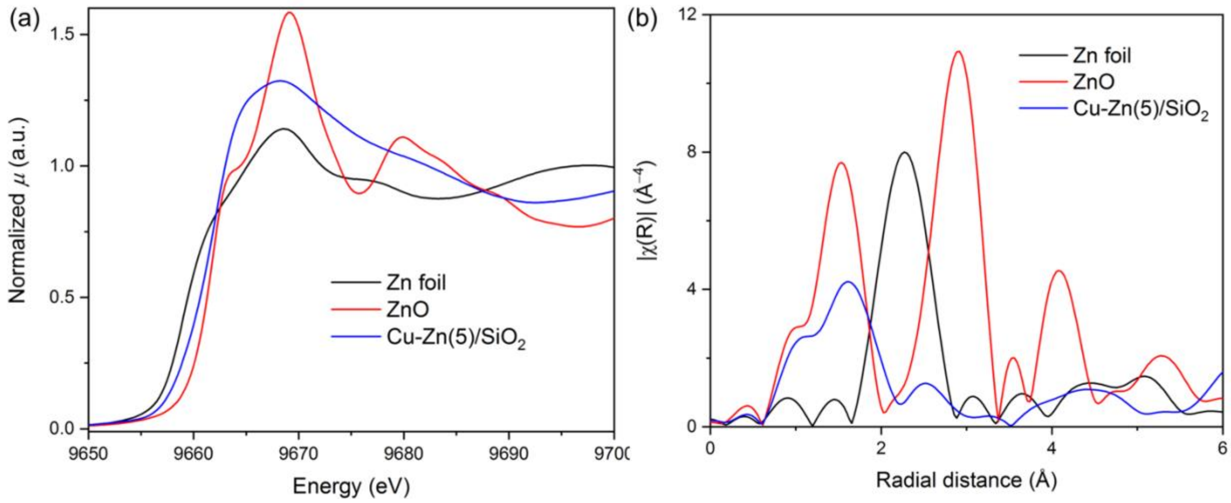

Figure S32. Zn K-edge XAS of Cu-$Zn(5)/SiO_2$ and references: (a) XANES. (b) Fourier-transform of the k^3-weighted EXAFS.

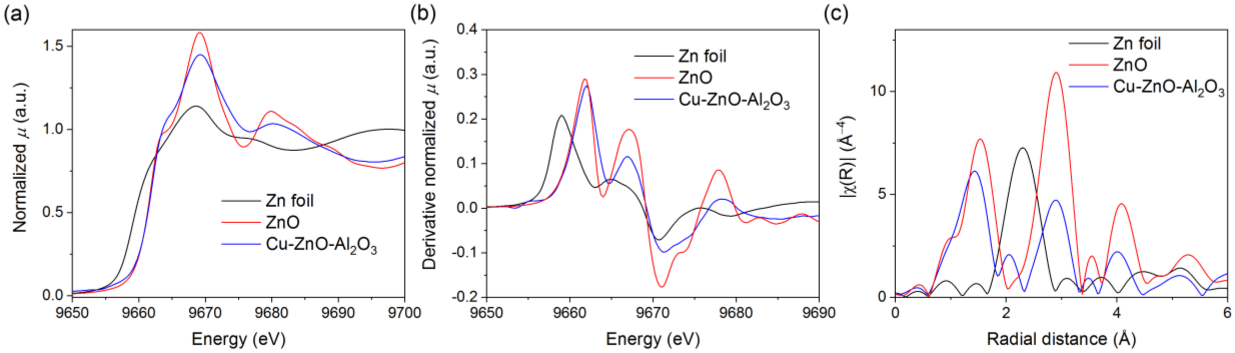

Figure S33. Zn K-edge XAS of Cu-ZnO-Al_2O_3. (a) XANES. (b) The first derivative of the XANES. (c) Fourier-transform of the k^3-weighted EXAFS.

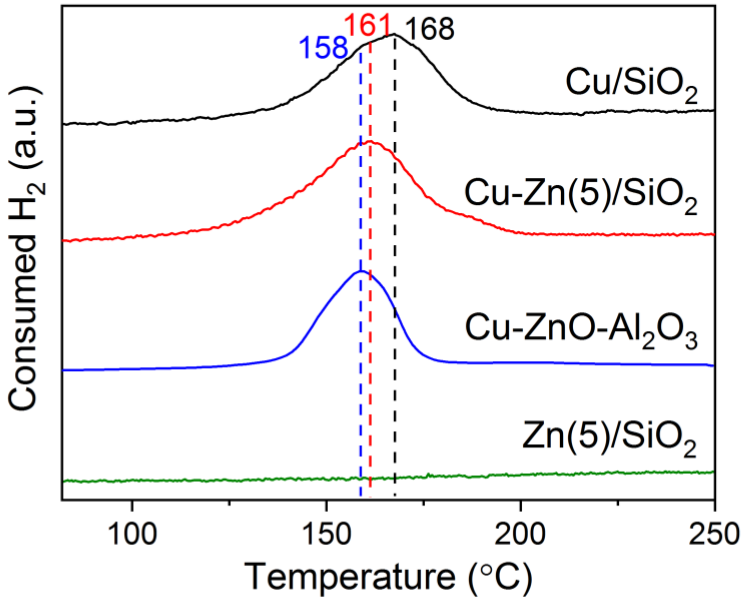

Figure S34. Temperature-programmed reduction in 5% H_2/Ar after oxidation of the specimen in 5% O_2/He at room temperature.

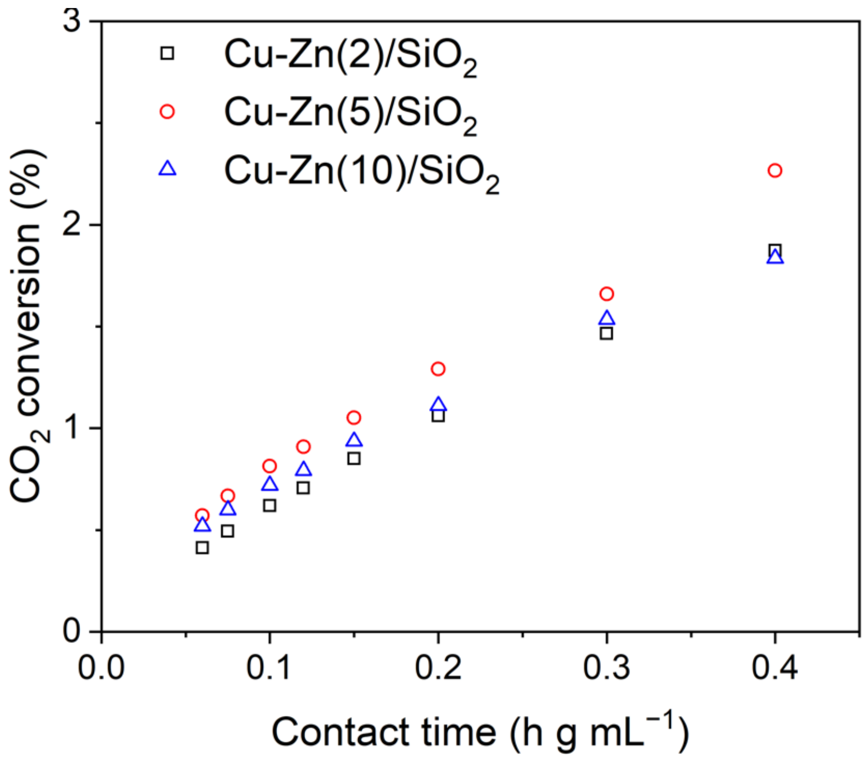

Figure S35. CO_2 conversion for Cu-Zn(2)/SiO_2, Cu-Zn(5)/SiO_2, and Cu-Zn(10)/SiO_2 with respect to contact time (230 °C, 25 bar, $H_2/CO_2/N_2$ = 3:1:1).

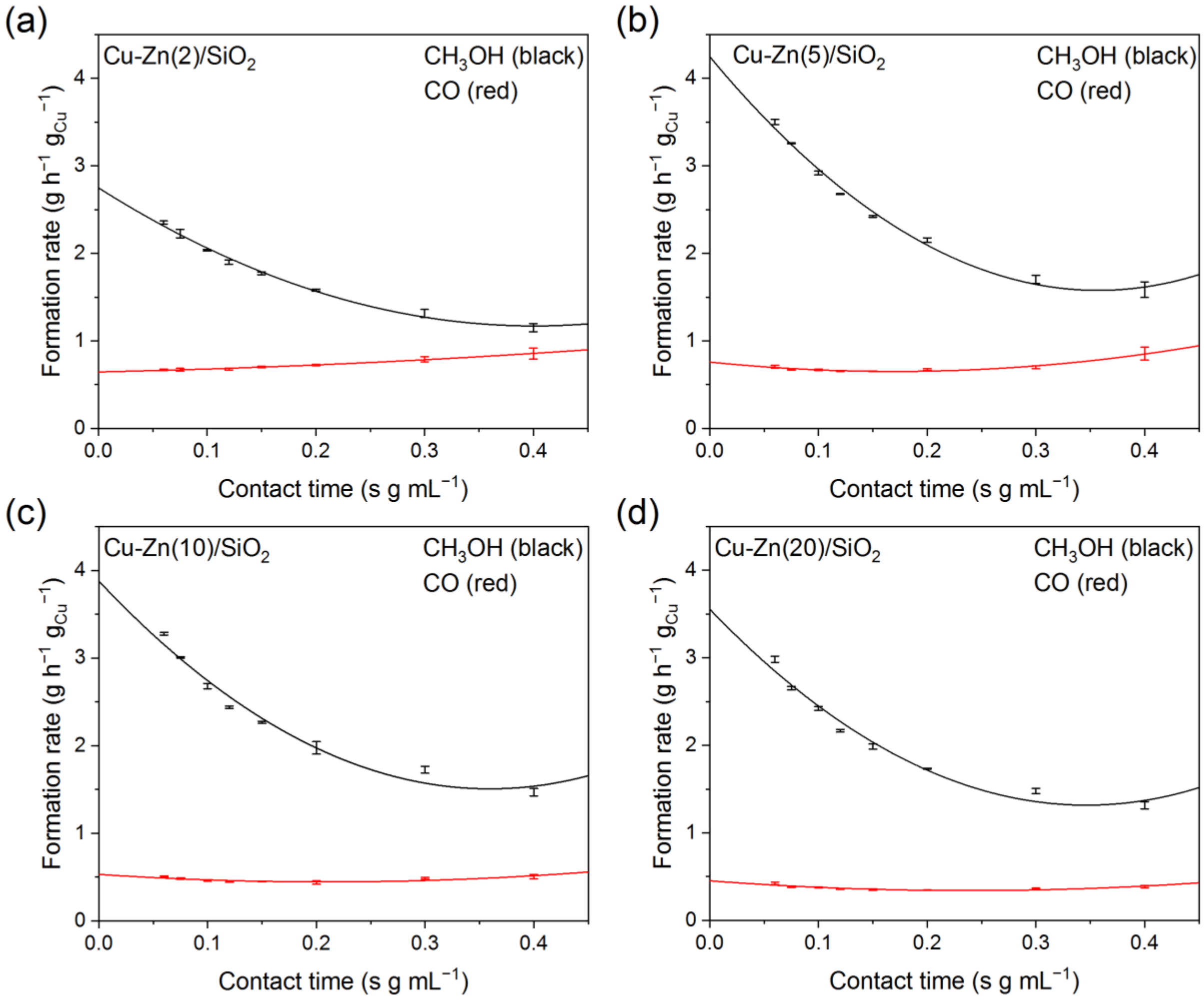

Figure S36. Formation rates of CH_3OH and CO for Cu-Zn(2)/SiO_2, Cu-Zn(5)/SiO_2, Cu-Zn(10)/SiO_2, or Cu-Zn(20)/SiO_2 with respect to contact time, extrapolated to zero conversion (zero contact time) with second-order polynomial fits.

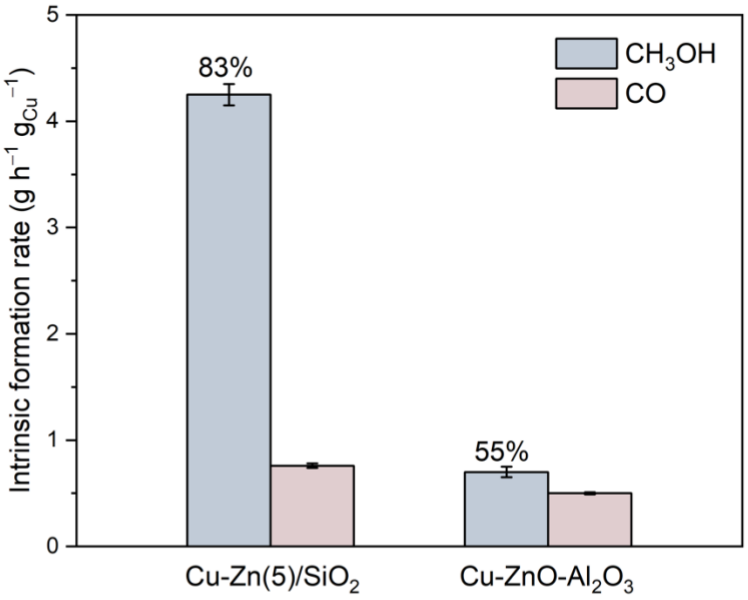

Figure S37. Intrinsic formation rates normalized per mass of Cu for Cu-Zn(5)/SiO_2 and Cu-ZnO-Al_2O_3 obtained by extrapolation to zero conversion (zero contact time) together with the respectively selectivities for CH_3OH, specified above the respective bars (230 °C, 25 bar, $H_2/CO_2/N_2$ = 3:1:1).

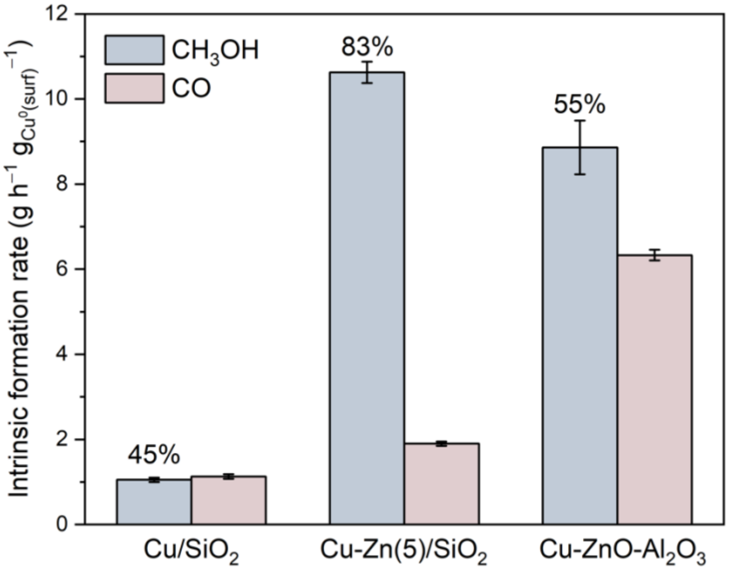

Figure S38. Intrinsic formation rates normalized per mass of surface Cu sites (denoted Cu^0(surf)) with the respective selectivities to CH_3OH (specified above the respective bars) for the catalysts tested (230 °C, 25 bar, $H_2/CO_2/N_2$ = 3:1:1).

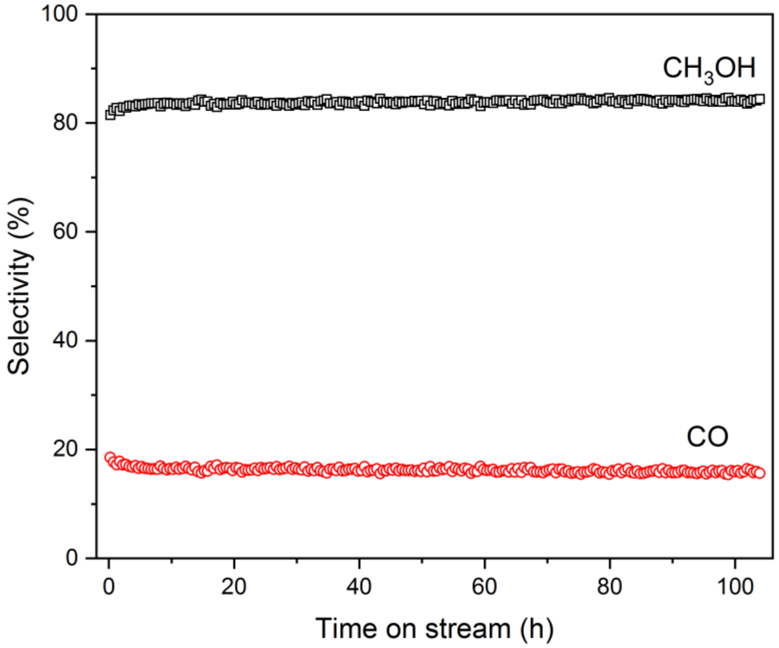

Figure S39. CH_3OH and CO selectivity at ca. 100 hours of TOS for Cu-Zn(5)/SiO_2 (230 °C, 25 bar, $H_2/CO_2/N_2$ = 3/1/1, contact time 0.06 s g mL^{-1}).

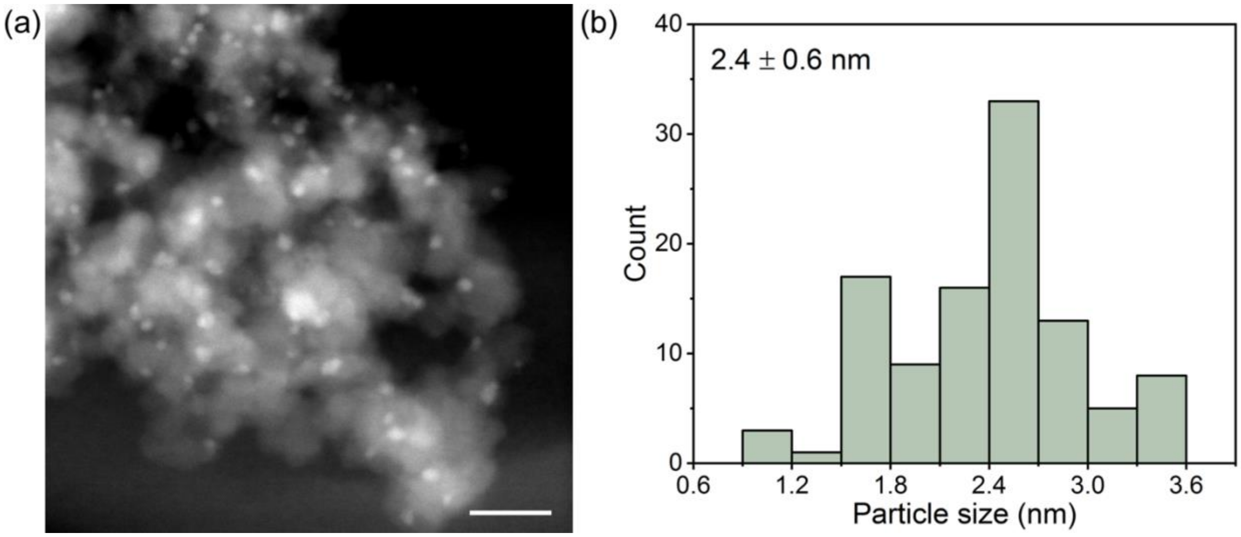

Figure S40. A representative TEM and particle size distribution of Cu-Zn(5)/$SiO_{2\text{-TOS100h}}$. (a) HAADF-STEM (scale bar: 20 nm). (b) Particle size distribution.

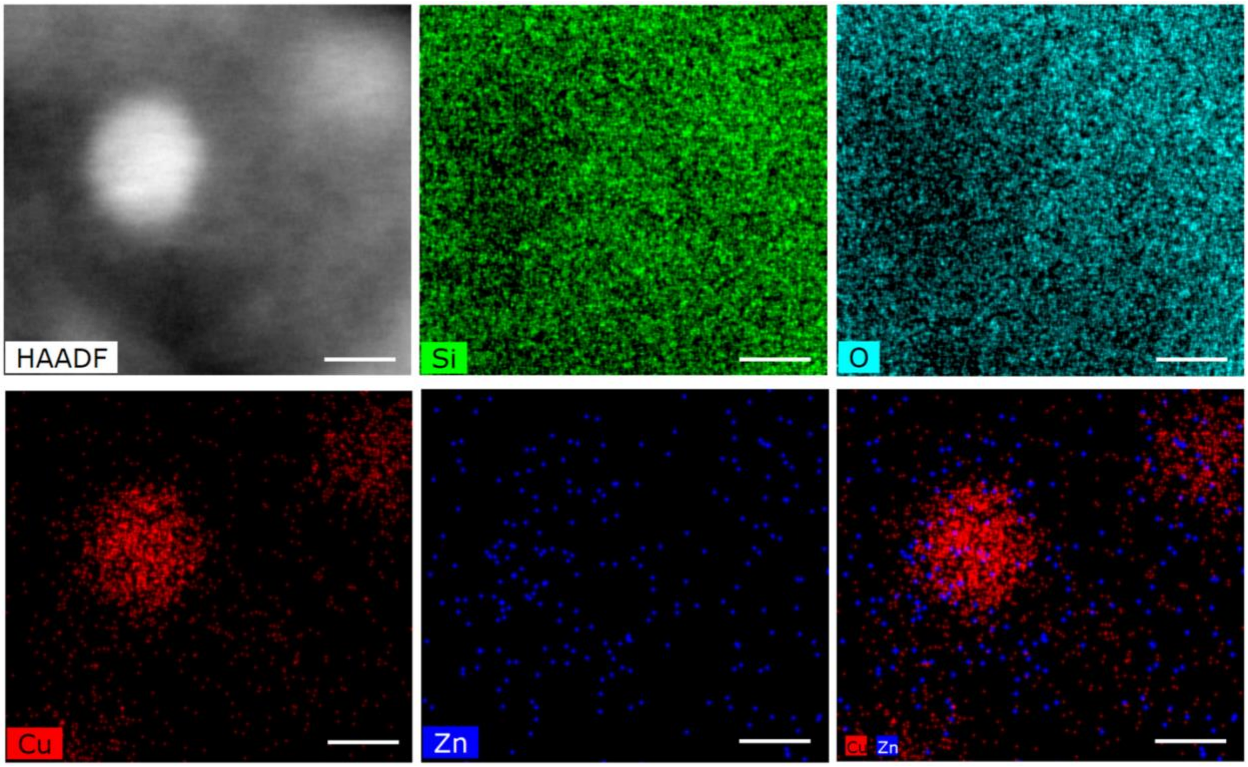

Figure S41. TEM-EDX of Cu-Zn(5)/$SiO_{2\text{-TOS100h}}$. Scale bar: 2 nm.

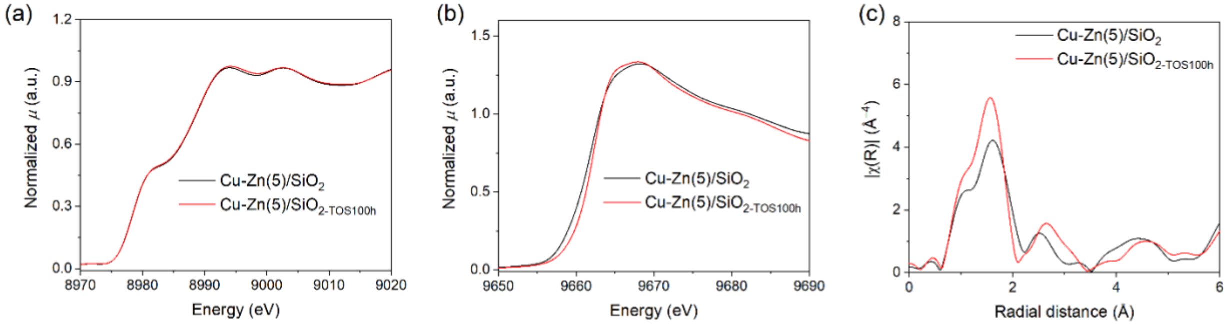

Figure S42. Cu and Zn K-edge XAS of fresh Cu-Zn(5)/SiO_2 and after 100 h of TOS. (a) Cu K-edge XANES. (b) Zn K-edge XANES. (c) Fourier-transform of the k^2-weighted Zn K-edge EXAFS.

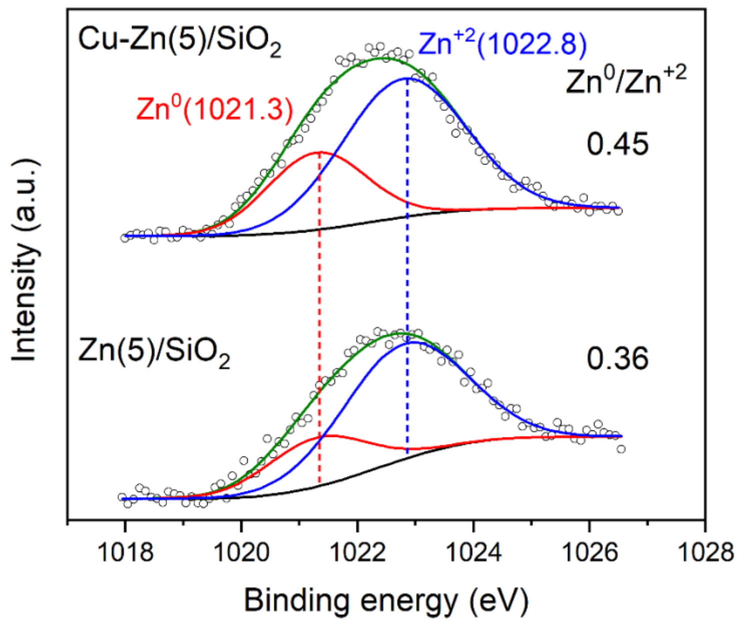

Figure S43. Peak deconvolution of the Zn $2p_{3/2}$ XPS spectra of the freshly reduced Cu-Zn(5)/SiO_2 and Zn(5)/SiO_2. Pretreatment conditions: 200 °C, 1 h, 300 mbar H_2.

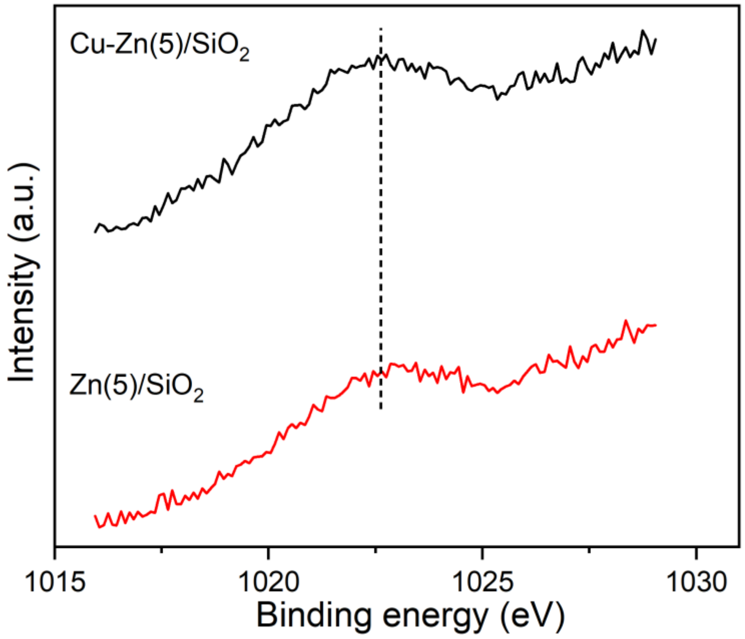

Figure S44. Zn $2p_{3/2}$ XPS spectra of Cu-Zn(5)/SiO_2 and Zn(5)/SiO_2 after exposure of the activated materials to 120 mbar of methanol vapor (230 °C, 1 h).

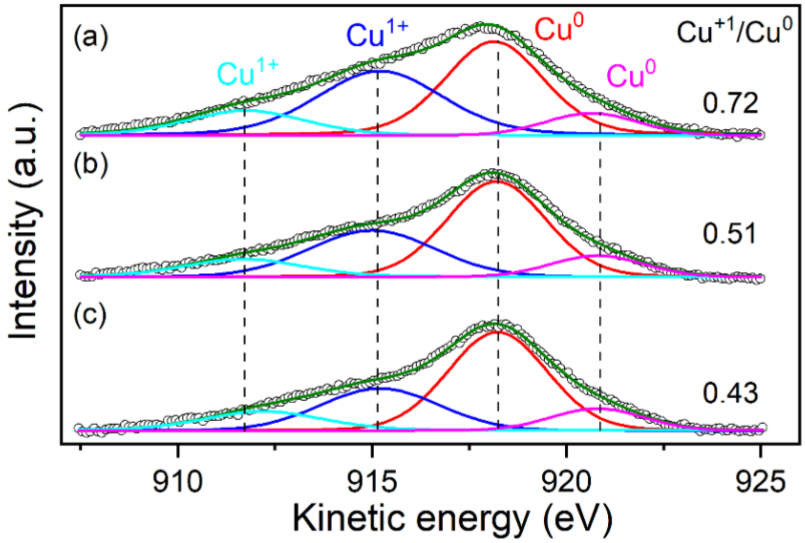

Figure S45. Cu LMM Auger spectra of Cu/SiO_2. (a) Passivated under 1% O_2/N_2 for 2 h. (b) Reduced under 300 mbar H_2 at 100 °C for 1 h. (c) Reduced under 300 mbar H_2 at 200 °C for 1 h.

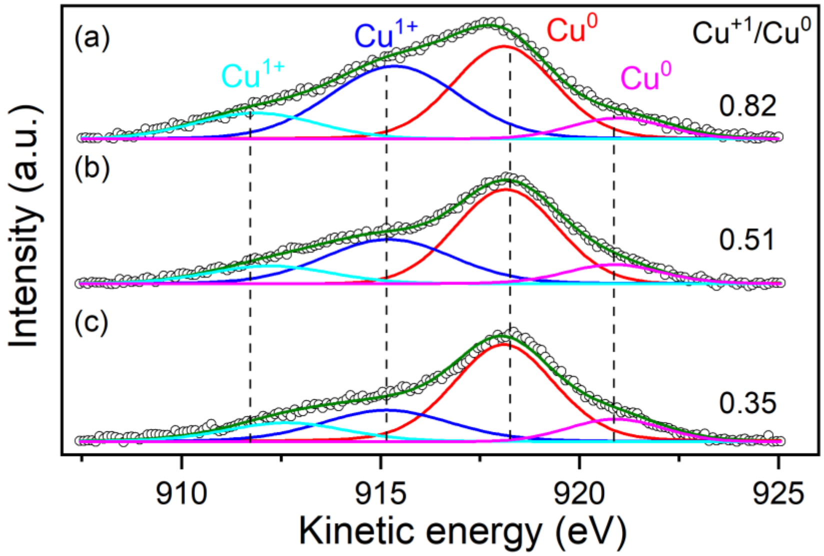

Figure S46. Cu LMM Auger spectra of Cu-Zn(5)/SiO_2. (a) Passivated under 1% O_2/N_2 for 2 h. (b) Reduced under 300 mbar H_2 at 100 °C for 1 h. (c) Reduced under 300 mbar H_2 at 200 °C for 1 h.

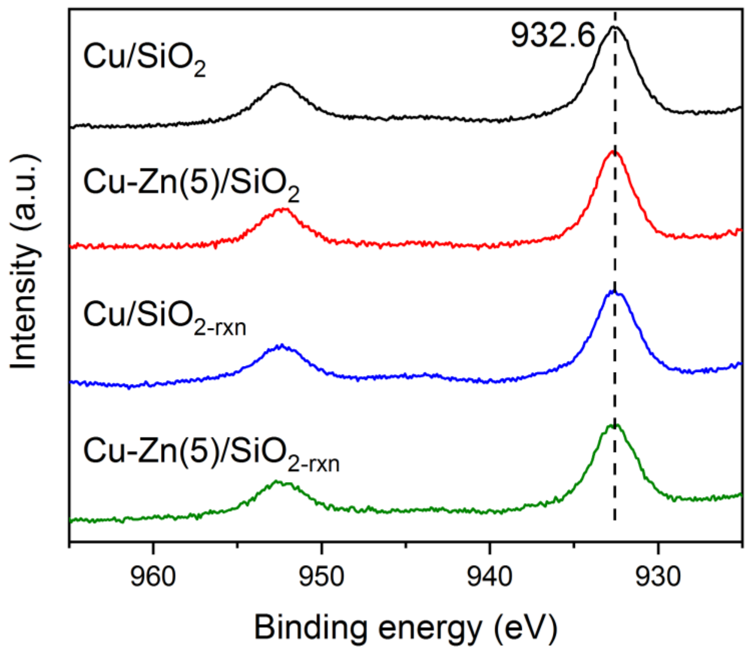

Figure S47. Cu 2p XPS spectra of Cu/SiO_2 and $Cu-Zn(5)/SiO_2$ before and after exposure to the reaction mixture of H_2 (300 mbar) and CO_2 (100 mbar) at 230 °C for 1 h. Cu 2p XPS region is not sensitive enough to separate contributions from Cu^0 and Cu^{+1}.

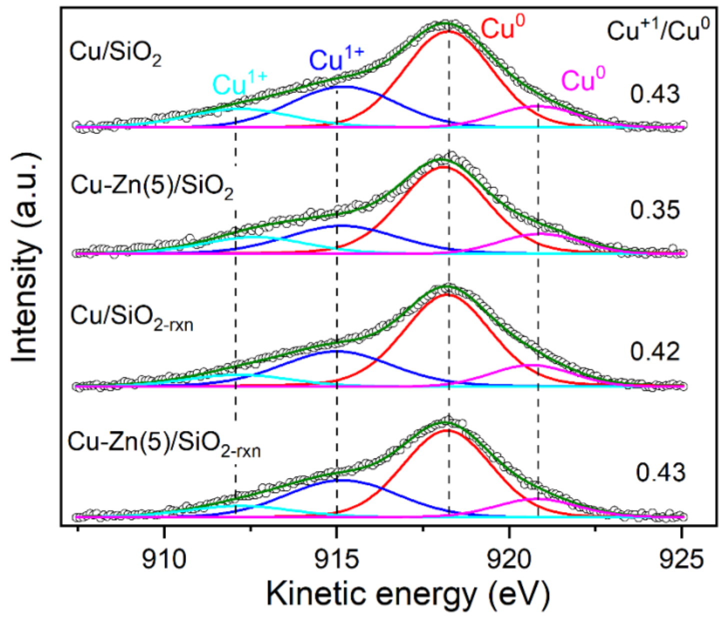

Figure S48. Cu LMM Auger spectra of Cu/SiO_2 and $Cu-Zn(5)/SiO_2$ pre-treated at 300 mbar of H_2 at 200 °C prior to and after their exposure to the reaction mixture of H_2 and CO_2 (300 and 100 mbar, respectively) at 230 °C for 1 h.

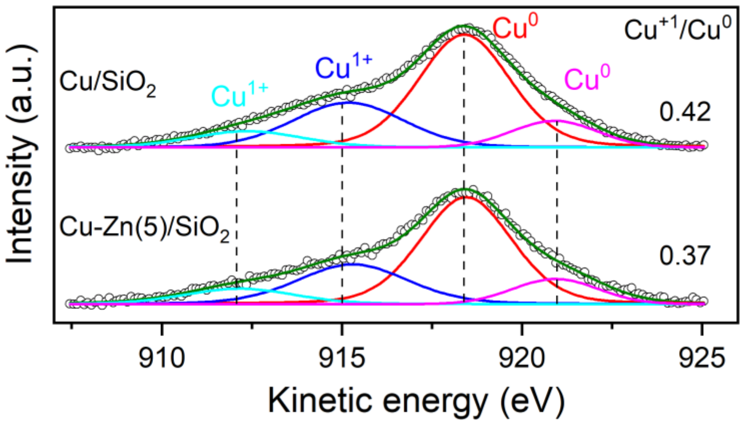

Figure S49. Cu LMM Auger spectra of activated Cu/SiO_2 and Cu-Zn(5)/SiO_2 after their exposure to methanol vapor (300 mbar, 230 °C, 1 h).

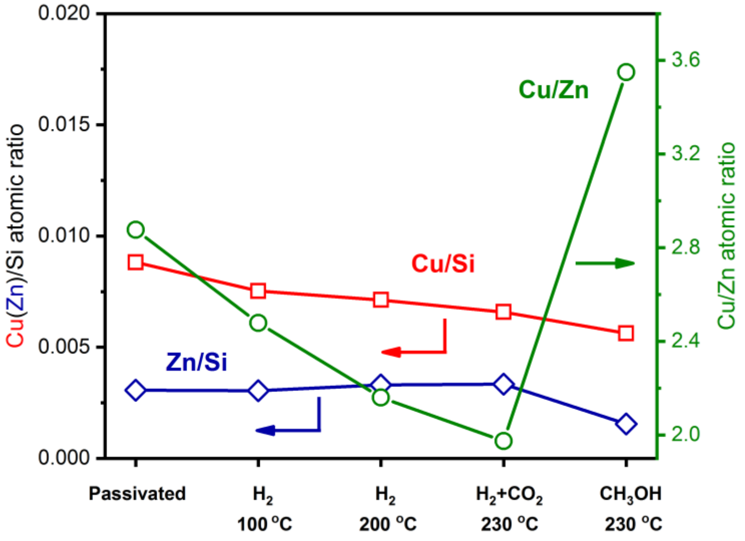

Figure S50. Cu(Zn)/Si atomic ratio of the Cu-Zn(5)/SiO_2 material from XPS.

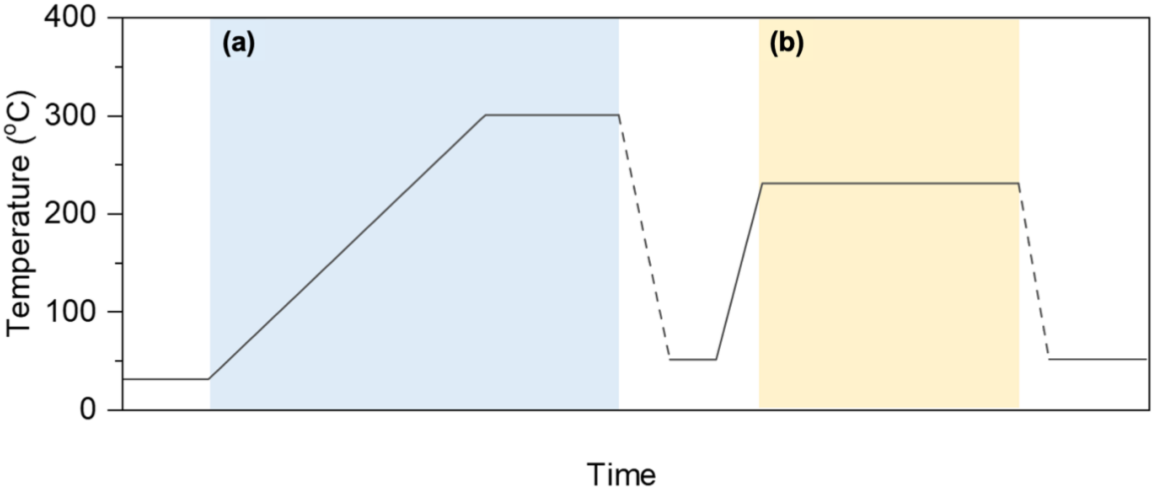

Figure S51. Schematic representation of the temperature profile during the *in situ* XAS experiment of passivated Cu-Zn(5)/SiO_2. (a) TPR (conditions: r.t. → 300 °C, H_2, 10 sccm, 1 bar, ramp: 5 °C min^{-1}), and (b) CO_2 hydrogenation reaction (conditions: 230 °C, H_2/Ar = 3:2 → H_2/CO_2/Ar = 3:1:1, 10 sccm, 11 bar).

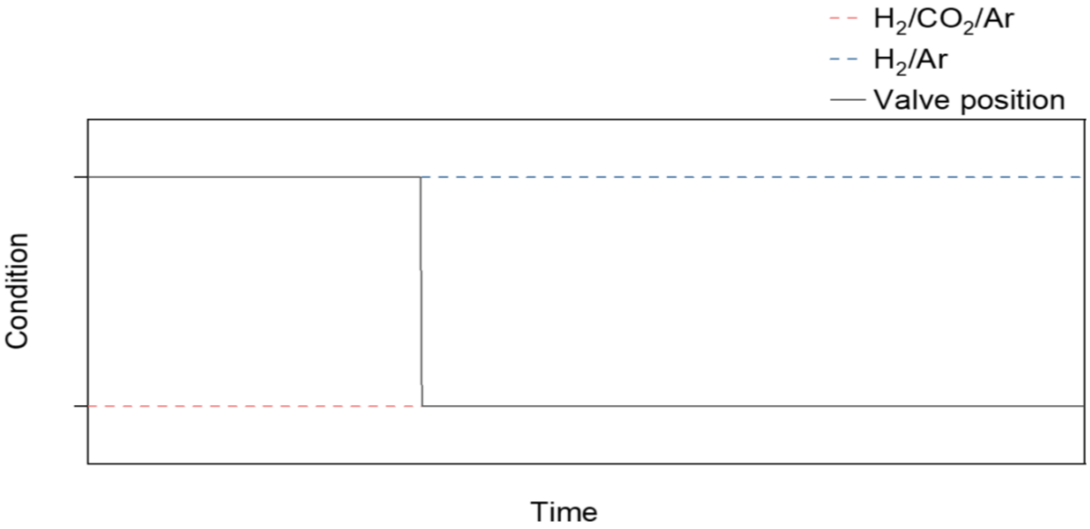

Figure S52. Schematic representation of a single gas switch experiment as shown in Figure S51b (conditions: 230 °C, H_2/Ar = 3:2 → H_2/CO_2/Ar = 3:1:1, 10 sccm, 11 bar).

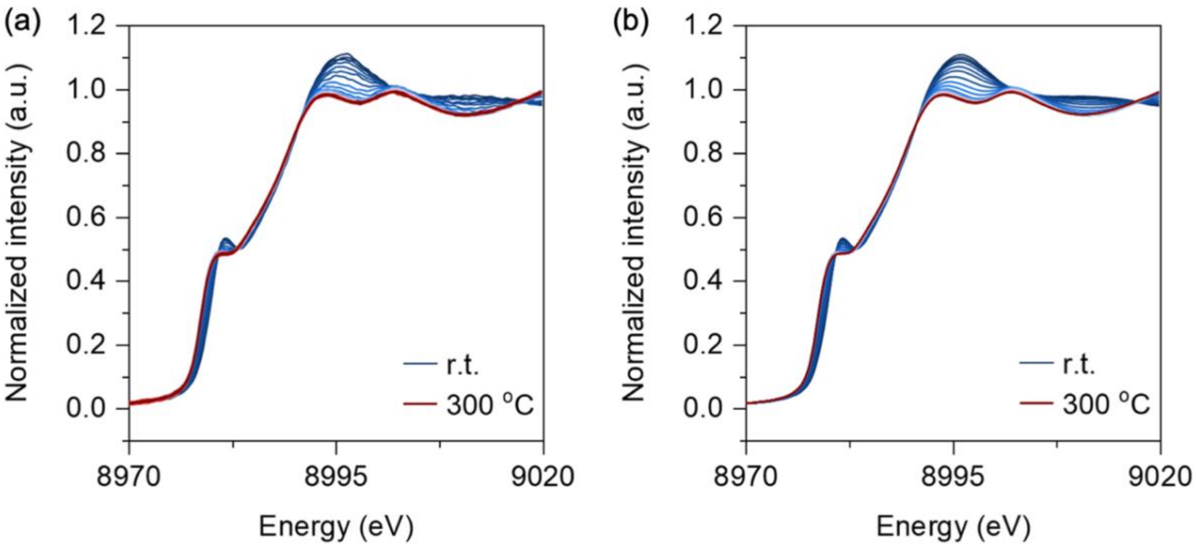

Figure S53. (a) Cu K edge XANES during TPR. (b) MCR-ALS modelled XANES spectra at the Cu K edge during the TPR process (conditions: r.t. → 300 °C, H_2, 10 sccm, 1 bar, ramp: 5 °C min^{-1}).

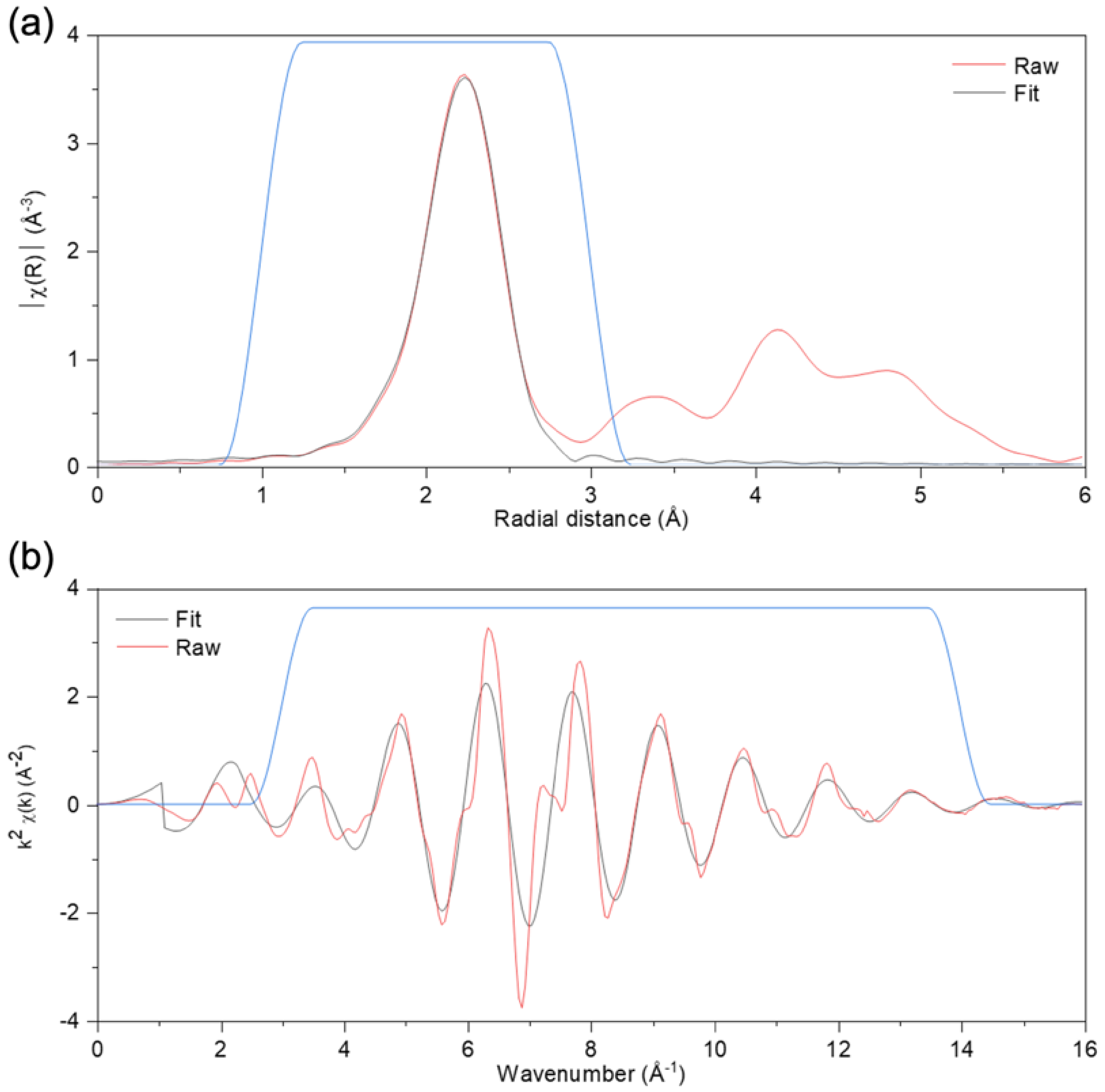

Figure S54. k^2-weighted Cu K edge EXAFS for Cu foil. (a) R-space (Window (blue) 1–3 Å, Hanning window, dk = 0.5). (b) K-space (Window (blue) 3.0–14.0 $Å^{-1}$, Hanning window, dk = 1). Fitting results are summarized in Table S7.

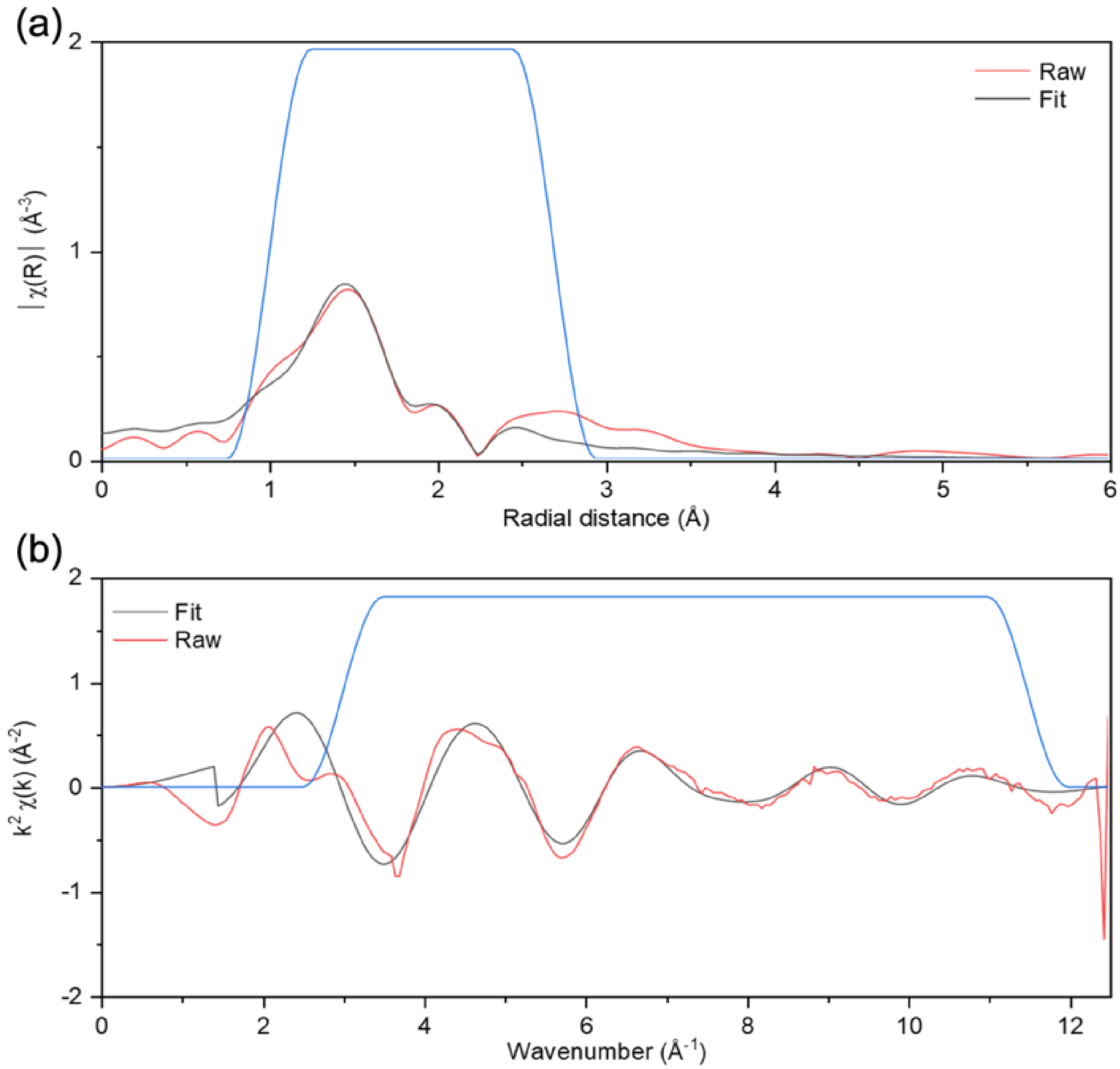

Figure S55. k^2-weighted Cu K edge EXAFS for passivated Cu-Zn(5)/SiO_2. (a) R-space (Window (blue) 1–2.7 Å, Hanning window, dk = 1). (b) K-space (Window (blue) 3.0-11.5 $Å^{-1}$, Hanning window, dk = 0.5). Fitting results are summarized in Table S7.

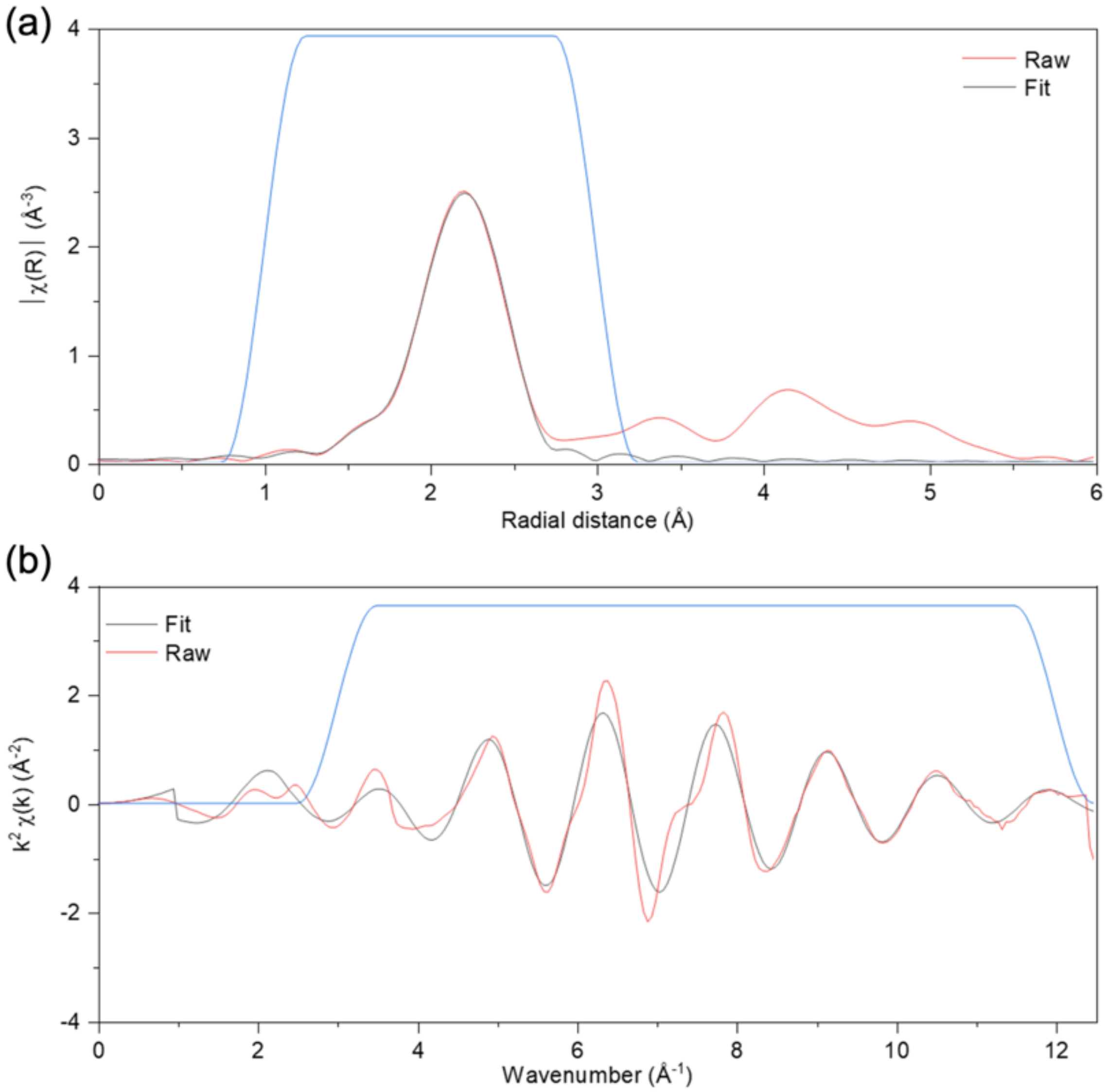

Figure S56. k^2-weighted Cu K edge EXAFS for Cu-Zn(5)/SiO_2 after hydrogen treatment and before the gas switching experiment. (a) R-space (Window (blue) 1–3 Å, Hanning window, dk = 1). (b) K-space (Window (blue) 3.0–12.0 $Å^{-1}$, Hanning window, dk = 0.5). Fitting results are summarized in Table S7.

In general, the results of the EXAFS fitting of passivated Cu-Zn(5)/SiO_2 prior to H_2 pretreatment are consistent with the presence of a Cu–O path (R_{Cu-O} = 1.87 ± 0.014 Å, CN_{Cu-O} = 2.1 ± 0.3), while after H_2 pre-treatment the EXAFS is dominated by metallic Cu-M paths (R_{Cu-M} = 2.55 ± 0.004 Å, CN_{Cu-M} = 9.9 ± 0.7), see Figures S55 and S56 and Table S7. We noted in the main text that the presence of a minor amount of Cu^0 in the passivated sample prior to H_2 treatment cannot be excluded.[16,17] Indeed, the inclusion of a Cu–Cu path in the EXAFS fitting of the passivated sample results in a Cu–M path with a low degeneracy (R_{Cu-M} = 2.52 ± 0.03 Å, CN_{Cu-M} = 0.6 ± 0.2), consistent with the presence of a small proportion of Cu in a reduced form, likely in the form of small clusters.

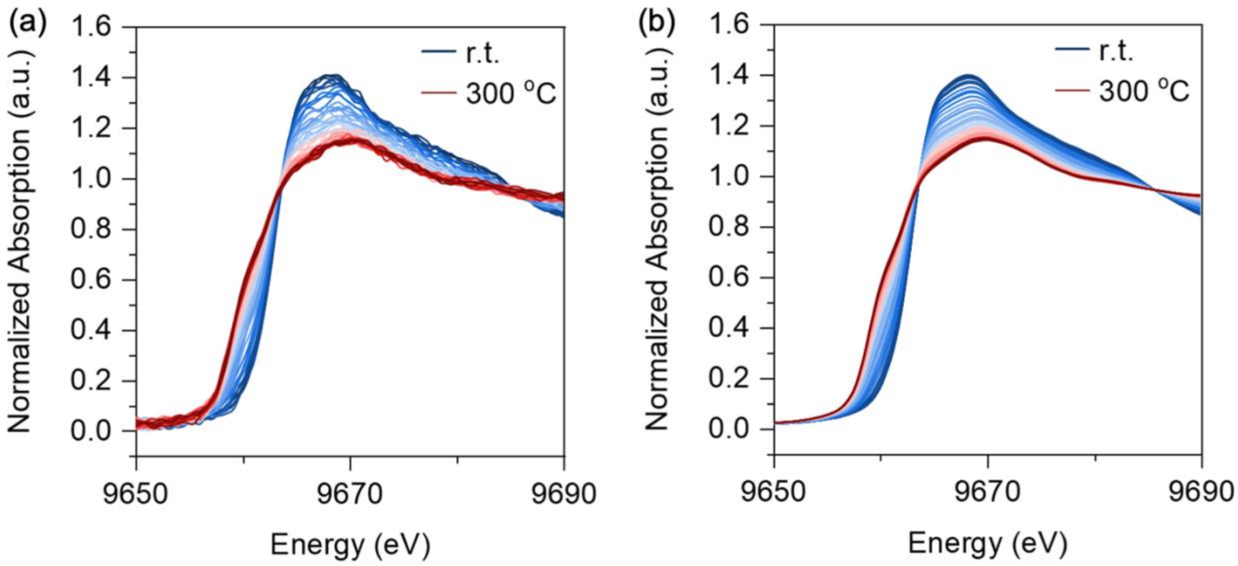

Figure S57. (a) Zn K edge XANES during TPR. (b) MCR modelled Zn K edge XANES spectra during TPR. Conditions: r.t. → 300 °C, H_2, 10 sccm, 1 bar, ramp: 5 °C min^{-1}.

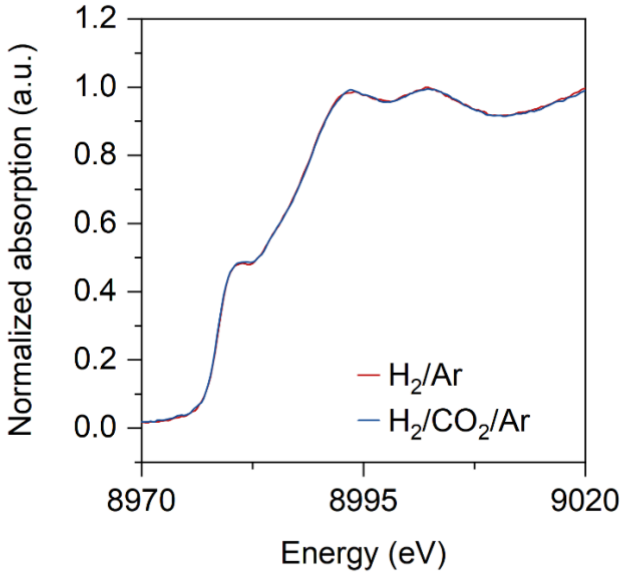

Figure S58. Cu K edge XANES before and after gas switching (i.e. introduction of CO_2). Conditions: 230 °C, 3:2 H_2/Ar → 3:1:1 H_2/CO_2/Ar, 10 sccm, 11 bar.

Differences between before and after the gas switch were not resolvable.

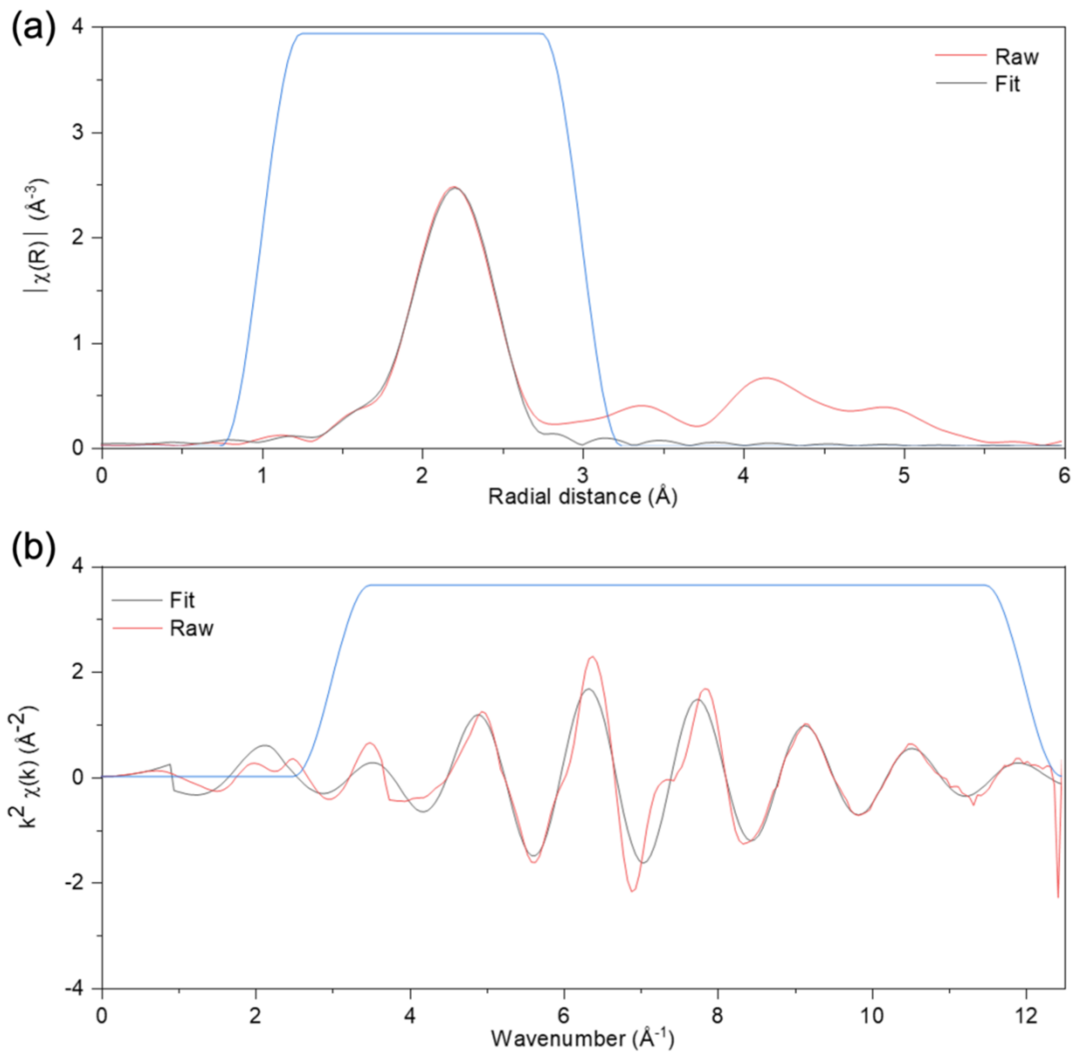

Figure S59. k^2-weighted Cu K edge EXAFS for Cu-Zn(5)/SiO_2 after the *in situ* gas switching experiment. (a) R-space (Window (blue) 1–3 Å, Hanning window, dk = 1). (b) K-space (Window (blue) 3.0–12.0 $Å^{-1}$, Hanning window, dk = 0.5).

Fitting results are summarized in Table S7.

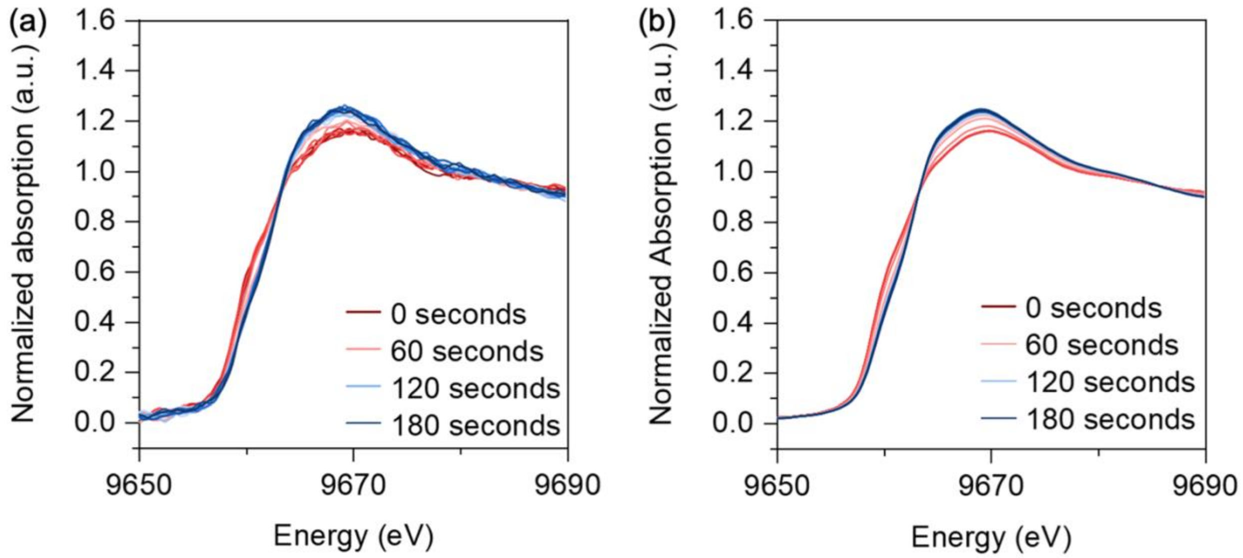

Figure S60. (a) Zn K edge XANES during gas switch (introduction of CO_2). (b) MCR modelled Zn K edge XANES spectra after the gas switch (introduction of CO_2). Conditions: 230 °C, 3:2 H_2/Ar → 3:1:1 H_2/CO_2/Ar, 10 sccm, 11 bar). The point of zero sec is defined as the time when CO_2 was introduced into the H_2 containing gas feed.

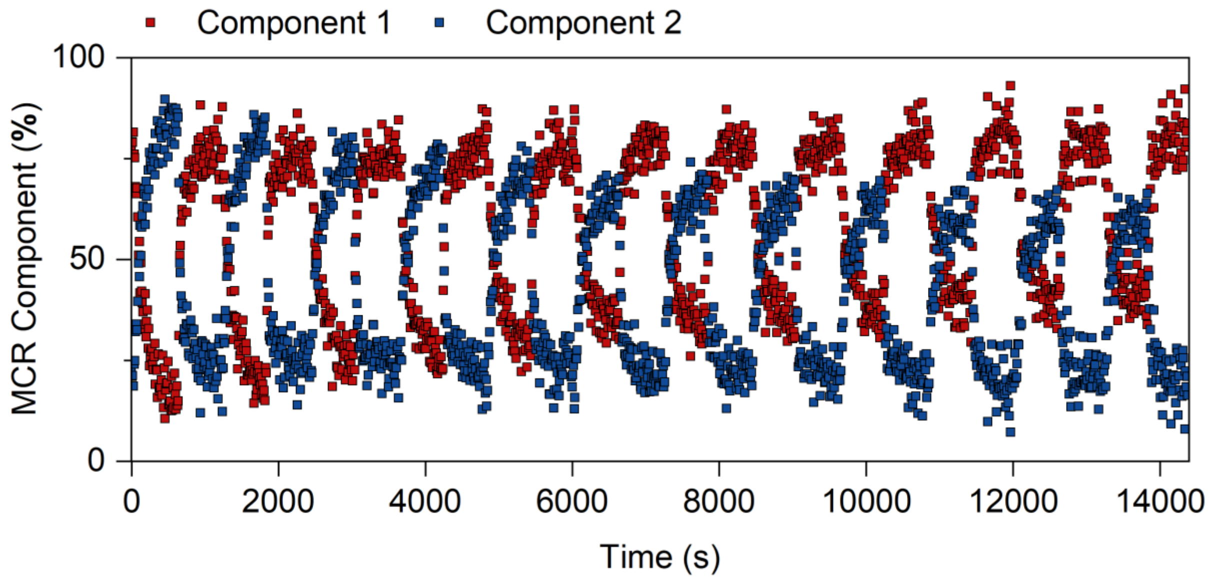

Figure S61. MCR-ALS analysis of the Zn K edge XANES during a multiple-cycle gas switching experiments between H_2/Ar (3:2) and H_2/CO_2/Ar (3:1:1) at 230 °C and 11 bar. Cycle length: 1200 seconds (600 sec + 600 sec), 20 mg Cu-Zn(5)/SiO_2 catalyst, 10 sccm.

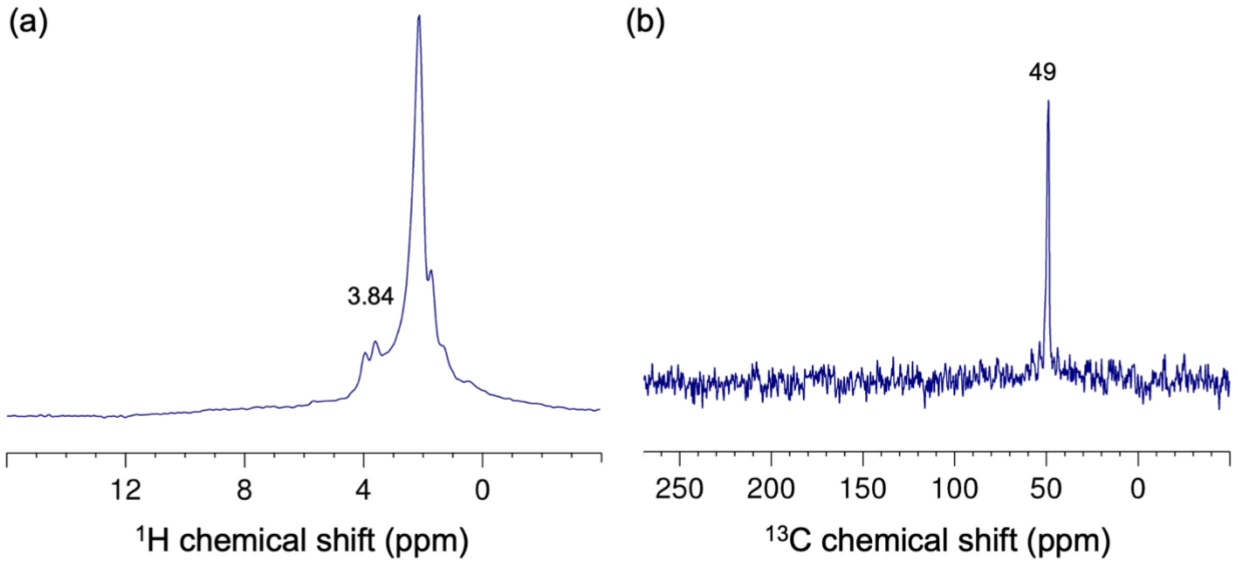

Figure S62. Solid-state MAS NMR of Cu-Zn(5)/SiO_2 after exposure to H_2 and $^{13}CO_2$ (H_2/$^{13}CO_2$ = 3/1, total pressure 5 bar, 230 °C, 12 h). (a) ^1H spectrum (B_0 = 9.4 T, 298 K, 4 mm probe, $\nu_L(^1H)$ = 400 MHz, NS = 64). (b) ^{13}C CP-MAS spectrum (B_0 = 9.4 T, 298 K, 4 mm probe, $\nu_L(^1H)$ = 400 MHz, $\nu_L(^{13}C)$ = 100.6 MHz, NS = 3688).

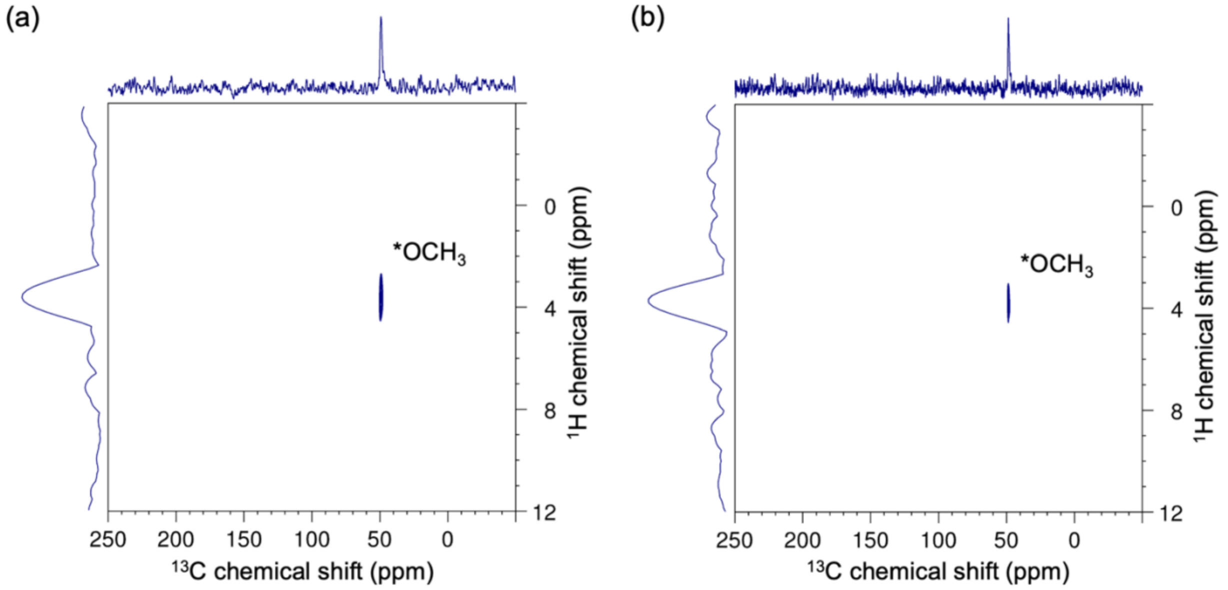

Figure S63. ^1H–^{13}C heteronuclear correlation (HETCOR) spectrum of (a) Cu/SiO_2 and (b) Cu-Zn(5)/SiO_2 after exposure to H_2 and $^{13}CO_2$ (H_2/$^{13}CO_2$ = 3:1, total pressure 5 bar, 230 °C, 12 h). 2D HETCOR NMR spectra were recorded with 128 scans in the direct and 64 increments in the indirect dimensions. For homonuclear decoupling, e-DUMBO scheme was used.

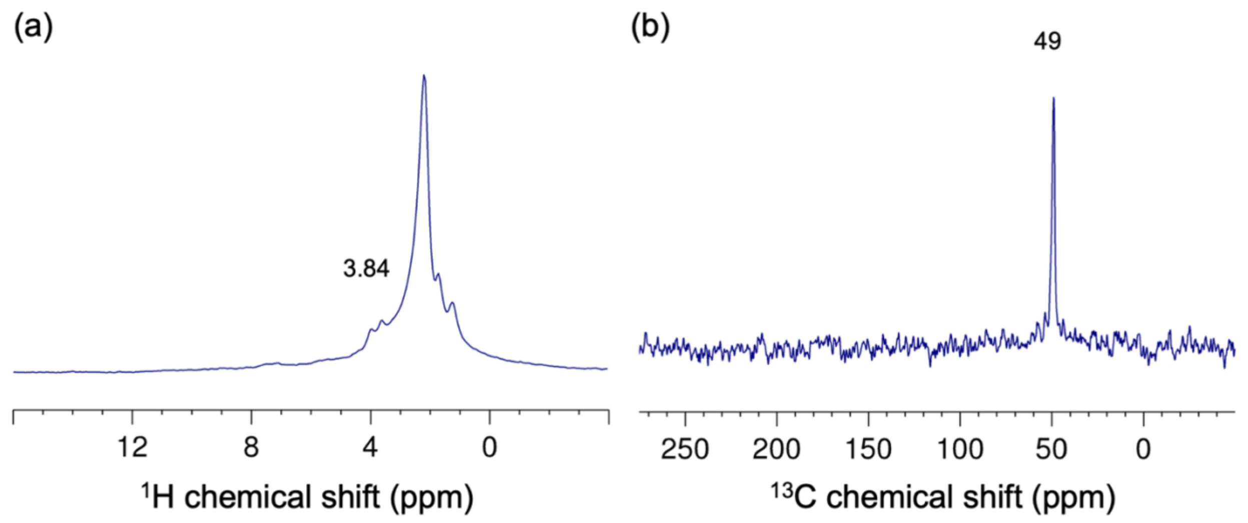

Figure S64. Solid-state MAS NMR of Cu/SiO_2 after exposure to H_2 and $^{13}CO_2$ ($H_2/^{13}CO_2$ = 3/1, total pressure 5 bar, 230 °C, 12 h). (a) ^1H spectrum (B_0 = 9.4 T, 298 K, 4 mm probe, $\nu_L(^1H)$ = 400 MHz, NS = 64). (b) ^{13}C CP-MAS spectrum (B_0 = 9.4 T, 298 K, 4 mm probe, $\nu_L(^1H)$ = 400 MHz, $\nu_L(^{13}C)$ = 100.6 MHz, NS = 12272).

Note that a related Cu/SiO_2 material reported earlier did provide a formate peak at 168 ppm in the ^{13}C CP-MAS spectrum and no methoxy peak.[15] The difference to the result of the experiment in this work likely stems from the different experimental conditions to prepare the labelled surface intermediates, i.e., the reaction vessel was cooled to –196 ºC and then evacuated at 10^{-5} mbar in this work (see experimental section for details).

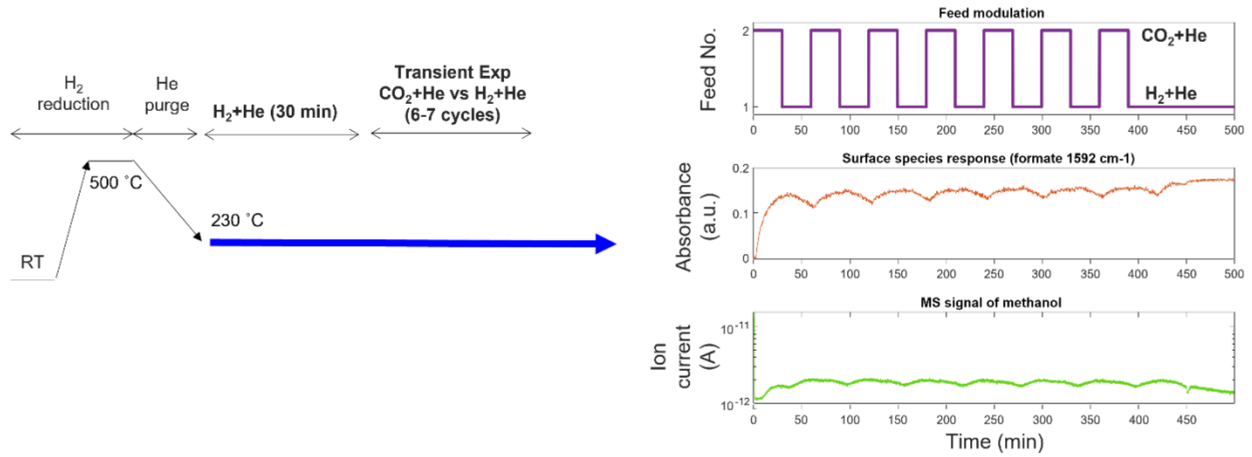

Figure S65. Experimental procedures for transient *operando* DRIFTS for modulated He/CO_2 and H_2/He feeds. Reduction condition: 500 °C under 20 ml min^{-1} of H_2 for 2 h. Reaction conditions: ca. 30 mg catalyst, 230 °C, 20 bar, He/CO_2 and H_2/He = 3:1, total flow rate 20 ml min^{-1}.

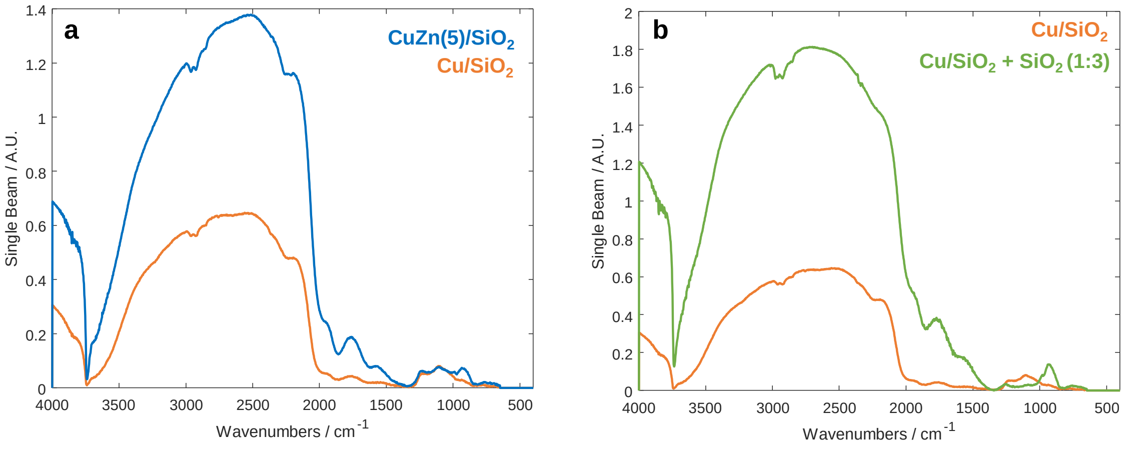

Figure S66. Single beam spectra (background) collected under He prior to the introduction of the CO_2 + H_2 feed: (a) without dilution and (b) with dilution with SiO_2. Reduction condition: 500 °C under 20 ml min^{-1} of H_2 for 2 h. Reaction conditions: ca. 30 mg catalyst, 230 °C, 20 bar, H_2/CO_2 = 3/1, total flow rate 20 ml min^{-1}.

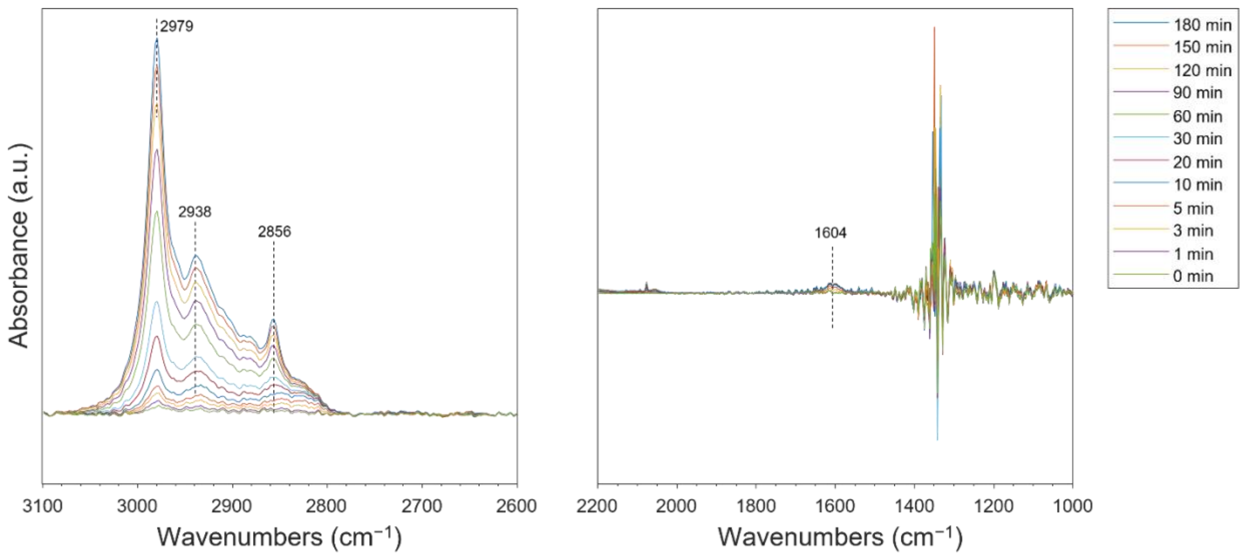

Figure S67. Time-resolved *operando* DRIFT spectra of surface species formed during CO_2 hydrogenation over Cu/SiO_2 diluted with SiO_2 (1:3). Reduction condition: 500 °C under 20 ml min^{-1} of H_2 for 2 h. Reaction conditions: ca. 30 mg catalyst, 230 °C, 20 bar, H_2/CO_2 = 3:1, total flow rate 20 ml min^{-1}.

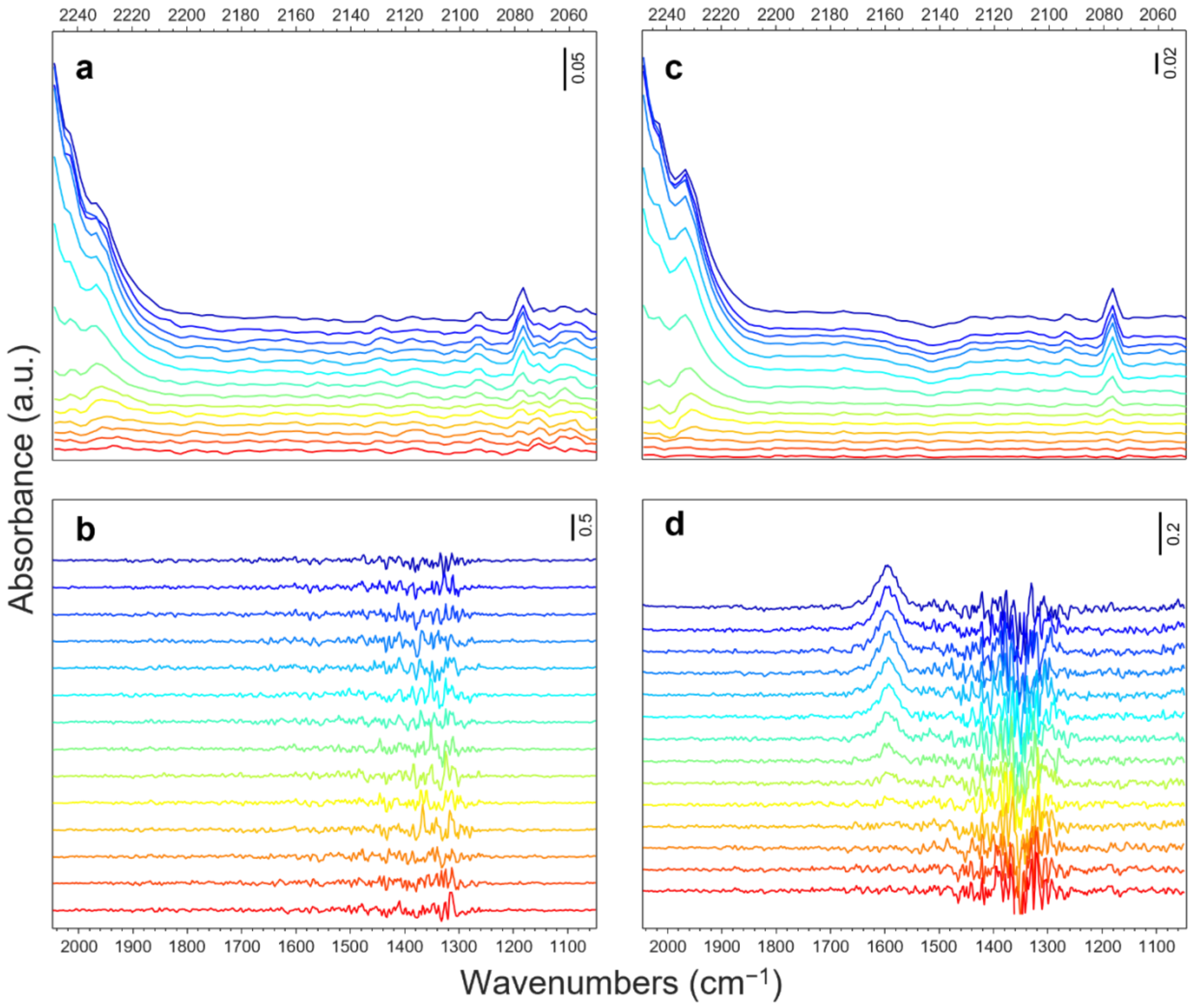

Figure S68. Time-resolved *operando* DRIFT spectra of surface species formed during CO_2 hydrogenation over (a–b) Cu/SiO_2 and (c–d) $Cu\text{-}Zn/SiO_2$ catalysts. Reduction condition: 500 °C under 20 ml min^{-1} of H_2 for 2 h. Reaction conditions: ca. 30 mg catalyst, 230 °C, 20 bar, H_2/CO_2 = 3:1, total flow rate 20 ml min^{-1}.

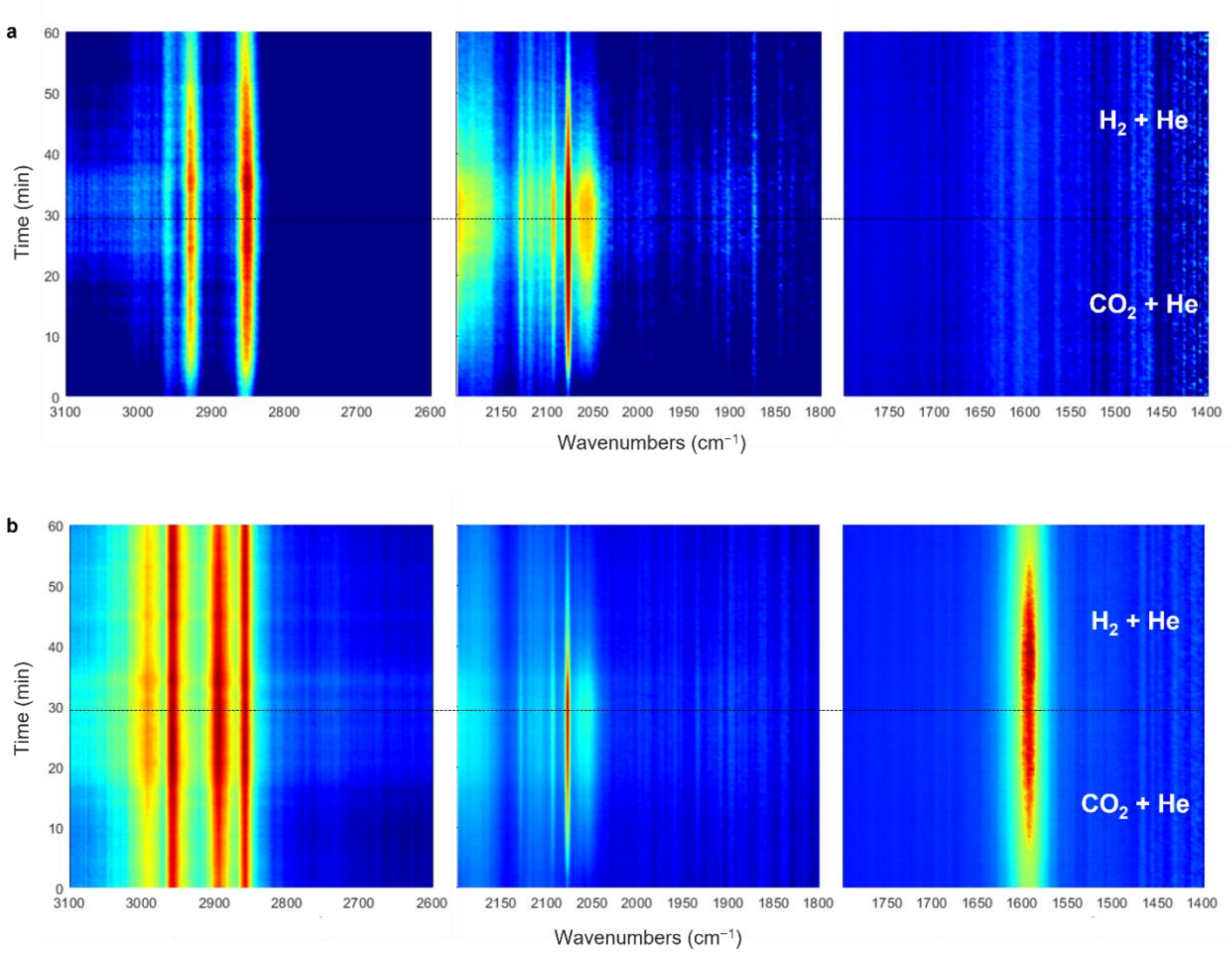

Figure S69. O*perando* DRIFTS during the switching between He+CO_2 and H_2+He feeds over (a) Cu/SiO_2 and (b) Cu-Zn(5)/SiO_2. Reduction condition: 500 °C under 20 ml min^{-1} of H_2 for 2 h. Reaction conditions: ca. 30 mg catalyst, 230 °C, 20 bar, He/CO_2 and H_2/He = 3:1, total flow rate 20 ml min^{-1}.

Supplementary Tables

Table S1. Cu and Zn loading of Cu-Zn/SiO_2 materials.

Material	BET surface area (m^2 g^{-1})	Pore volume (cm^3 g^{-1})	Average pore size (nm)
SiO_2	296	1.6	26.5
Cu/SiO_2	268	1.4	26.6
Zn(5)/SiO_2	291	1.5	26.6
Cu-Zn(5)/SiO_2	247	1.4	26.6

Table S2. Cu and Zn loading and Cu particle size of the materials prepared.

Material	Cu loading (wt%)[a]	Zn loading before H_2 treatment (wt%)[a]	Zn loading after H_2 treatment (wt%)[a]	Cu particle size (nm)[b]
Cu/SiO_2	2.0	–	–	2.9 ± 0.4
Cu-Zn(5)/SiO_2	2.1	0.5	0.4	1.8 ± 0.4
CuMes-Et_2Zn(5)/SiO_2	2.1	0.5	–	1.8 ± 0.3
Cu-Zn(5)/$SiO_{2\text{-TOS100h}}$	2.1	–	0.4	2.4 ± 0.6
Zn(5)/SiO_2	–	0.8	0.2	–
Zn(5)-Cu/SiO_2	2.1	0.6	0.4	3.4 ± 1.0
Cu-Zn(5)/$SiO_{2\text{-H2-300}}$	2.1	0.5	0.5	1.8 ± 0.4
Cu_{red}-Zn(5)/SiO_2	2.1	0.7	0.4	3.1 ± 0.6
Cu-Zn(5)/$SiO_{2\text{-air}}$	2.1	0.5	0.3	2.7 ± 0.4

[a] From ICP-OES. [b] From TEM.

Table S3. Cu-Cu shell parameters obtained by Cu K-edge EXAFS fitting of the different catalysts.

Material	CN	σ^2 (Å^2)	ΔE_0 (eV)	R (Å)	R-factor
Cu foil	12	0.010(1)	6(1)	2.553(7)	0.0029
Cu/SiO_2	9.3(9)	0.011(1)	5(1)	2.538(6)	0.0019
Cu-Zn(5)/SiO_2	5.8(9)	0.010(1)	2(2)	2.52(1)	0.0056

For quantitative EXAFS analysis, least square fittings implemented in the ARTEMIS software were applied.[11] Theoretical phases and amplitudes were obtained through self-consistent *ab initio* calculations with the FEFF6 code using the Cu f.c.c structure. The fitted variables were: coordination number (CN),

interatomic distance *R*, bond length disorder factors (Debye Waller factors, DW), and energy shift for the first Cu–Cu coordination shell. The photoelectron reference energy ΔE_0 was fitted for each spectrum independently. The amplitude reduction factor S_0^2= 0.77 was obtained from fitting of the corresponding Cu foil, by fixing the CN to 12. Data fitting was carried out in the range of 1.7–2.8 Å and a window dr of 0.5, and the Fourier transform was carried out for k = 3.0–11.3 Å^{-1}.

Table S4. Quantity of surface Cu^0 sites determined through N_2O titration.

Material	Surface Cu^0 sites (μmol g_{cat}^{-1})	Surface Cu^0 sites/Cu loading (%)
Cu/SiO_2	124	39
Cu-Zn(2)/SiO_2	133	40
Cu-Zn(5)/SiO_2	126	40
Cu-Zn(10)/SiO_2	101	29
Zn(5)/SiO_2	ND[a]	ND
Cu-Zn(5)/SiO_{2-300}	169	51
Cu_{red}-Zn(5)/SiO_2	94	28
Cu-Zn(5)/SiO_{2-air}	42	13
Cu-ZnO-Al_2O_3[b]	735	8

[a] Not detected. [b] Commercial catalyst.

Table S5. Total H_2 consumption calculated from bulk TPR (Cu was pre-oxidized to CuO by O_2).

Material	Peak temperature (°C)	Total H_2 consumption (μmol g_{cat}^{-1})	Total H_2 consumption /theoretical H_2 consumption (%)[a]
Cu/SiO_2	167	303	96
Cu-Zn(5)/SiO_2	161	311	98
Zn(5)/SiO_2	–	ND[b]	ND
Cu_{red}-Zn(5)/SiO_2	169	319	97
Cu-Zn(5)/SiO_{2-air}	154	314	95
Cu-ZnO-Al_2O_3[c]	158	7293	78

[a] Theoretical H_2 consumption was calculated from Cu loading with $CuO + H_2 \rightarrow Cu + H_2O$.

[b] Not detected. [c] Commercial catalyst.

Table S6. Comparison of the catalysts in this study with benchmark catalysts in the literature.

Catalyst	Synthesis method	Reduction temp. (°C)	CH_3OH formation rate ($g\ h^{-1}\ g_{Cu}^{-1}$)	CH_3OH sel. (%)	Ref.
Cu-Zn(2)/SiO_2	SOMC+ALD[a]	500	2.75	79	This study[b]
Cu-Zn(5)/SiO_2	SOMC+ALD	500	4.25	83	This study
Cu-Zn(10)/SiO_2	SOMC+ALD	500	3.88	86	This study
Cu-Zn(20)/SiO_2	SOMC+ALD	500	3.55	87	This study
Cu/SiO_2	SOMC	500	0.41	45	This study
Cu-ZnO-Al_2O_3[c]	CPT[d]	250	0.70	55	This study
Cu-Zn/SiO_2[e]	SOMC	500	1.60	86	Ref[18]
Cu-ZnO-Al_2O_3[f]	CPT	250	0.62	55	Ref[19]
Cu-ZnO-Al_2O_3[g]	CPT	350	0.11	23	Ref[20]
Cu-ZnO-Al_2O_3[h]	NP[i]	250	0.41	NP	Ref[21]
Cu-ZnO-Al_2O_3[j]	CPT	400	0.73	54	Ref[22]

[a] SOMC: surface organometallic chemistry, ALD: atomic layer deposition. [b] Reaction conditions in this study: 230 °C, 25 bar, $H_2/CO_2/N_2$ = 3/1/1. Intrinsic formation rates/selectivities were used in this study. [c] Commercial Cu-ZnO-Al_2O_3 catalyst (Alfa Aesar). [d] CPT: co-precipitation. [e] Reaction conditions: 230 °C, 25 bar, $H_2/CO_2/N_2$ = 3/1/1. Intrinsic formation rate and selectivity. [f] Reaction conditions: 230 °C, 30 bar, H_2/CO_2=3/1. [g] Reaction conditions: 230 °C, 20 bar, H_2/CO_2=3/1. [h] Reaction conditions: 200 °C, 30 bar, H_2/CO_2=3/1. [i] NP: not provided. [j] Reaction conditions: 260 °C, 15 bar, H_2/CO_2/Ar=72/24/4.

Table S7. Summary of Cu K-edge EXAFS fitting results obtained from the *in situ* XAS experiment.

Material	Path	ΔE_0 (eV)	CN	R (Å)	σ^2 (Å^2)
Cu Foil	Cu-Cu	4.5 ± 0.5	12[a]	2.55 ± 0.002	0.009 ± 0.0004
Passivated	Cu-O	7.9 ± 2	2.1 ± 0.3	1.87 ± 0.014	0.005 ± 0.002
	Cu-M		0.6 ± 0.2	2.52 ± 0.03	0.010[b]
Pre-reaction	Cu-M	3.5 ± 0.7	9.9 ± 0.7	2.55 ± 0.004	0.009 ± 0.0005
Post-reaction	Cu-M	3.4 ± 0.6	9.6 ± 0.6	2.55 ± 0.004	0.010 ± 0.0005

[a] Coordination number was fixed for this fit. Amplitude reduction factor was determined as 0.876, and this value was carried forward for subsequent fittings.

[b] To reduce the number of variables, this parameter was fixed in this fit.

Table S8. Assignment of surface species in DRIFTS experiments.

Wavenumber (cm^{-1})	Vibrational mode	Band assignment	Ref.
2996	v_{as}(C–H)	Adsorbed methanol (CH_3OH^*)	[23,24]
2958	v_{as}(C–H)	Methoxy (CH_3O^*) on SiO_2	[23,24]
2842	v_{as}(C–H)	Methoxy (CH_3O^*) on ZnO	[24]
2936	*v(C–H)*	Bidentate formate (μ-$HCOO^*$) on Cu	[25–27]
2926	v_{as}(C–H)	Methoxy (CH_3O^*) on Cu	[23]
2921	v_s (C–H)	Adsorbed methanol (CH_3OH^*)	[23,24]
2893	*v*(C–H)	Bidentate Formate (μ-$HCOO^*$) on ZnO	[24]
2858	v_s (C–H)	Methoxy (CH_3O^*) on SiO_2	[23]
2853	*v(C–O) + δ(C–H)*	Bidentate formate (μ-$HCOO^*$) on Cu	[25–27]
2852	v_s (C–H)	Adsorbed methanol (CH_3OH^*)	[23,24]
2830	v_s (C–H)	Methoxy (CH_3O^*) on ZnO	[24]
2815	v_s (C–H)	Methoxy (CH_3O^*) on Cu	[23]
2129		Combination band of CO_2	[28]
2094		Combination band of CO_2	[28]
2077		Combination band of CO_2	[28]
2056		Combination band of CO_2	[28]
1604	*v (C–O)*	Bidentate formate (μ-$HCOO^*$) on Cu	[29]
1590	*v* (C–O)	Bidentate formate (μ-$HCOO^*$) on ZnO	[29]

References

(1) Copéret, C.; Comas-Vives, A.; Conley, M. P.; Estes, D. P.; Fedorov, A.; Mougel, V.; Nagae, H.; Núñez-Zarur, F.; Zhizhko, P. A. Surface Organometallic and Coordination Chemistry toward Single-Site Heterogeneous Catalysts: Strategies, Methods, Structures, and Activities. *Chem. Rev.* **2016**, *116*, 323–421.

(2) Lam, E.; Larmier, K.; Wolf, P.; Tada, S.; Safonova, O. V.; Copéret, C. Isolated Zr Surface Sites on Silica Promote Hydrogenation of CO_2 to CH_3OH in Supported Cu Catalysts. *J. Am. Chem. Soc.* **2018**, *140*, 10530–10535.

(3) Zhou, H.; Chen, Z.; López, A. V.; López, E. D.; Lam, E.; Tsoukalou, A.; Willinger, E.; Kuznetsov, D. A.; Mance, D.; Kierzkowska, A.; Donat, F.; Abdala, P. M.; Comas-Vives, A.; Copéret, C.; Fedorov, A.; Müller, C. R. Engineering the Cu/Mo_2CT_x (MXene) Interface to Drive CO_2 Hydrogenation to Methanol. *Nat. Catal.* **2021**, *4*, 860–871.

(4) Behrens, M.; Studt, F.; Kasatkin, I.; Kühl, S.; Hävecker, M.; Abild-Pedersen, F.; Zander, S.; Girgsdies, F.; Kurr, P.; Kniep, B.-L.; Tovar, M.; Fischer, R. W.; Nørskov, J. K.; Schlögl, R. The Active Site of Methanol Synthesis over Cu/ZnO/Al_2O_3 Industrial Catalysts. *Science* **2012**, *336*, 893–897.

(5) Lee, J. S.; Lee, K. H.; Lee, S. Y.; Kim, Y. G. A Comparative Study of Methanol Synthesis from CO_2/H_2 and CO/H_2 over a Cu/ZnO/Al_2O_3 Catalyst. *J. Catal.* **1993**, *144*, 414–424.

(6) Brunauer, S.; Emmett, P. H.; Teller, E. Adsorption of Gases in Multimolecular Layers. *J. Am. Chem. Soc.* **1938**, *60*, 309–319.

(7) Barrett, E. P.; Joyner, L. G.; Halenda, P. P. The Determination of Pore Volume and Area Distributions in Porous Substances. I. Computations from Nitrogen Isotherms. *J. Am. Chem. Soc.* **1951**, *73*, 373–380.

(8) Scofield, J. H. Hartree-Slater Subshell Photoionization Cross-Sections at 1254 and 1487 EV. *J. Electron Spectrosc. Relat. Phenom.* **1976**, *8*, 129–137.

(9) Lomachenko, K. A.; Molokova, A. Yu.; Atzori, C.; Mathon, O. Quantification of Adsorbates by X-Ray Absorption Spectroscopy: Getting TGA-like Information for Free. *J. Phys. Chem. C* **2022**, *126*, 5175–5179.

(10) Clark, A. H.; Imbao, J.; Frahm, R.; Nachtegaal, M. ProQEXAFS: A Highly Optimized Parallelized Rapid Processing Software for QEXAFS Data. *J. Synchrotron Radiat.* **2020**, *27*, 551–557.

(11) Ravel, B.; Newville, M. ATHENA, ARTEMIS, HEPHAESTUS: Data Analysis for X-Ray Absorption Spectroscopy Using IFEFFIT. *J. Synchrotron Radiat.* **2005**, *12*, 537–541.

(12) Tsoukalou, A.; Abdala, P. M.; Stoian, D.; Huang, X.; Willinger, M.-G.; Fedorov, A.; Müller, C. R. Structural Evolution and Dynamics of an In_2O_3 Catalyst for CO_2 Hydrogenation to Methanol: An Operando XAS-XRD and In Situ TEM Study. *J. Am. Chem. Soc.* **2019**, *141*, 13497–13505.

(13) Urakawa, A.; Bürgi, T.; Baiker, A. Sensitivity Enhancement and Dynamic Behavior Analysis by Modulation Excitation Spectroscopy: Principle and Application in Heterogeneous Catalysis. *Chem. Eng. Sci.* **2008**, *63*, 4902–4909.

(14) Jaumot, J.; de Juan, A.; Tauler, R. MCR-ALS GUI 2.0: New Features and Applications. *Chemom. Intell. Lab. Syst.* **2015**, *140*, 1–12.

(15) Larmier, K.; Liao, W.-C.; Tada, S.; Lam, E.; Verel, R.; Bansode, A.; Urakawa, A.; Comas-Vives, A.; Copéret, C. CO_2-to-Methanol Hydrogenation on Zirconia-Supported Copper Nanoparticles: Reaction Intermediates and the Role of the Metal–Support Interface. *Angew. Chem. Int. Ed.* **2017**, *56*, 2318–2323.

(16) Alfke, J. L.; Müller, A.; Clark, A. H.; Cervellino, A.; Plodinec, M.; Comas-Vives, A.; Copéret, C.; Safonova, O. V. BCC-Cu Nanoparticles: From a Transient to a Stable Allotrope by Tuning Size and Reaction Conditions. *Phys. Chem. Chem. Phys.* **2022**, *24*, 24429–24438.

(17) Oyanagi, H.; Sun, Z. H.; Jiang, Y.; Uehara, M.; Nakamura, H.; Yamashita, K.; Orimoto, Y.; Zhang, L.; Lee, C.; Fukano, A.; Maeda, H. Small Copper Clusters Studied by X-Ray Absorption near-Edge Structure. *J. Appl. Phys.* **2012**, *111*, 084315.

(18) Lam, E.; Noh, G.; Larmier, K.; Safonova, O. V.; Copéret, C. CO_2 Hydrogenation on Cu-Catalysts Generated from ZnII Single-Sites: Enhanced CH_3OH Selectivity Compared to $Cu/ZnO/Al_2O_3$. *J. Catal.* **2021**, *394*, 266–272.

(19) Jun, K.-W.; Shen, W.-J.; Rama Rao, K. S.; Lee, K.-W. Residual Sodium Effect on the Catalytic Activity of $Cu/ZnO/Al_2O_3$ in Methanol Synthesis from CO_2 Hydrogenation. *Appl. Catal. Gen.* **1998**, *174*, 231–238.

(20) Allam, D.; Cheknoun, S.; Hocine, S. Operating Conditions and Composition Effect on the Hydrogenation of Carbon Dioxide Performed over $CuO/ZnO/Al_2O_3$ Catalysts. *Bull. Chem. React. Eng. Catal.* **2019**, *14*, 604.

(21) Liang, B.; Ma, J.; Su, X.; Yang, C.; Duan, H.; Zhou, H.; Deng, S.; Li, L.; Huang, Y. Investigation on Deactivation of $Cu/ZnO/Al_2O_3$ Catalyst for CO_2 Hydrogenation to Methanol. *Ind. Eng. Chem. Res.* **2019**, *58*, 9030–9037.

(22) Zabilskiy, M.; Sushkevich, V. L.; Palagin, D.; Newton, M. A.; Krumeich, F.; van Bokhoven, J. A. The Unique Interplay between Copper and Zinc during Catalytic Carbon Dioxide Hydrogenation to Methanol. *Nat. Commun.* **2020**, *11*, 2409.

(23) Millar, G. J.; Rochester, C. H.; Waugh, K. C. Infrared Study of the Adsorption of Methanol on Oxidised and Reduced Cu/SiO_2 Catalysts. *J. Chem. Soc. Faraday Trans.* **1991**, *87*, 2795–2804.

(24) Millar, G. J.; Rochester, C. H.; Waugh, K. C. Evidence for the Adsorption of Molecules at Special Sites Located at Copper/Zinc Oxide Interfaces. Part 2.—A Fourier-Transform Infrared Spectroscopy Study of Methanol Adsorption on Reduced and Oxidised $Cu/ZnO/SiO_2$ Catalysts. *J. Chem. Soc. Faraday Trans.* **1992**, *88*, 2257–2261.

(25) Millar, G. J.; Rochester, C. H.; Waugh, K. C. An FTIR Study of the Adsorption of Formic Acid and Formaldehyde on Potassium-Promoted Cu/SiO_2 Catalysts. *J. Catal.* **1995**, *155*, 52–58.

(26) Millar, G. J.; Rochester, C. H.; Waugh, K. C. Infrared Study of Methyl Formate and Formaldehyde Adsorption on Reduced and Oxidised Silica-Supported Copper Catalysts. *J. Chem. Soc. Faraday Trans.* **1991**, *87*, 2785–2793.

(27) Millar, G. J.; Newton, D.; Bowmaker, G. A.; Cooney, R. P. In Situ FT-IR Investigation of Formic Acid Adsorption on Reduced and Reoxidized Copper Catalysts. *Appl. Spectrosc.* **1994**, *48*, 827–832.

(28) Fehr, S. M.; Krossing, I. Spectroscopic Signatures of Pressurized Carbon Dioxide in Diffuse Reflectance Infrared Spectroscopy of Heterogeneous Catalysts. *ChemCatChem* **2020**, *12*, 2622–2629.

(29) Neophytides, S. G.; Marchi, A. J.; Froment, G. F. Methanol Synthesis by Means of Diffuse Reflectance Infrared Fourier Transform and Temperature-Programmed Reaction Spectroscopy. *Appl. Catal. Gen.* **1992**, *86*, 45–64.
